# Identification of novel amides and alkaloids as putative inhibitors of dopamine transporter for schizophrenia using computer-aided virtual screening

**DOI:** 10.3389/fphar.2025.1509263

**Published:** 2025-04-08

**Authors:** Iqra Ahmad, Anam Tassawer, Muhammad Umer Khan, Muhammad Shehroz, Umar Nishan, Sheheryar Sheheryar, Hanna Dib, Mohamed A. O. Abdelfattah, Mohibullah Shah

**Affiliations:** ^1^ Department of Biochemistry, Bahauddin Zakariya University, Multan, Pakistan; ^2^ Institute of Molecular Biology and Biotechnology, The University of Lahore, Lahore, Pakistan; ^3^ Department of Bioinformatics, Kohsar University Murree, Murree, Pakistan; ^4^ Hainan International Joint Research Center of Marine Advanced Photoelectric Functional Materials, College of Chemistry and Chemical Engineering, Hainan Normal University, Haikou, China; ^5^ Department of Chemistry, Kohat University of Science and Technology, Kohat, Pakistan; ^6^ Department of Animal Science, Federal University of Ceara, Fortaleza, Brazil; ^7^ College of Engineering and Technology, American University of the Middle East, Egaila 54200, Kuwait

**Keywords:** medicinal plants, antipsychotic treatment, phytochemicals, drug designing, in-silico

## Abstract

Schizophrenia is a complex psychiatric disorder marked by delusions, memory impairments, hallucinations, disorganized behavior, and severe cognitive deficits. Targeting the dopamine transporter (DAT) protein is promising for treating cognitive symptoms, especially in patients resistant to antipsychotic treatments. In this study, phytochemicals from six medicinal plants underwent virtual screening, and molecular dynamics simulation to identify potential agents targeting DAT. Key drug-like properties, safety, and biological activity were evaluated for identified hits. Pharmacokinetic simulation and pharmacophoric analysis were also performed. Among 990 screened phytochemicals, three alkaloids and six amides, predominantly from *Piper retrofractum,* and one diterpene were identified as potential antischizophrenic agents based on their stronger binding affinities and favorable docking scores compared to the standard (Lumateperone). Amides showed more potential for DAT than alkaloids. The dynamic behavior and stability of the top three amides, namely, Chenoalbicin, Dipiperamide G, and Lyciumamide C, were evaluated using various molecular dynamics analyses. RMSD (Root Mean Square Deviation), RMSF (Root Mean Square Fluctuation), Rg (Radius of Gyration), and SASA (Solvent Accessible Surface Area) analyses demonstrated favorable characteristics for all three ligands. However, binding free energy, cross-correlation, PCA (Principal Component Analysis) and FEL (Free Energy Landscape) analyses indicated that Lyciumamide C exhibited the highest stability and binding affinity in dynamic environments, Pharmacophoric features highlighted the distinct interacting components for the top three amides. Pharmacokinetic simulations revealed significant peak concentrations and sustained levels can be indicated as Lyciumamide C > Chenoalbicin > Dipiperamide G. The higher and more sustained brain concentrations of Lyciumamide C suggest its most promising pharmacokinetic profile for targeting DAT. Overall, our screened metabolites followed drug-ability criteria and require further experimental validation.

## Introduction

Schizophrenia is a chronic and complicated brain disorder that is characterized by a wide range of symptoms, such as hallucinations, delusions, disordered behavior or speech, and most importantly, impairments in cognitive abilities (Olasupo et al., 2020). The pathophysiology of different brain disorders is based on dysfunction in dopamine levels or in dopamine neurotransmission, such as ADHD (attention deficit hyperactivity disorder), bipolar disorder, depression, and Parkinson’s disease (Li et al., 2016). Psychosis and schizophrenia are similar disorders concerning symptoms (Đorđević et al., 2022). There are around 24 million people with schizophrenia worldwide (Abi-Dargham, 2014).

The cornerstone of schizophrenia therapy is antipsychotic medication. Such drugs have a broad spectrum of mechanisms and interact with a variety of naturally occurring dopamine receptors (DAT) for neurotransmission (Zipursky et al., 2007). Dysfunction of DAT involved in the pathogenesis of different neuropsychiatric disorders hallucination such as mood depressive disorder, depression, anxiety, and schizophrenia (Nikolaus et al., 2019).

DAT is a membrane protein that helps transport dopamine from the synaptic junction, collects it in nearby cells, and disconnects the neurotransmission (Olasupo et al., 2021). Dopamine system dysregulation is central to the pathophysiology of schizophrenia, particularly in the manifestation of psychotic symptoms such as hallucinations and delusions (Sonnenschein et al., 2020). Dopamine levels in the cortical region are important for cognitive functions, and the level of DAT is higher in the frontal lobes in patients with schizophrenic and mood-depressive disorders (Sekiguchi et al., 2023), which causes an imbalance in dopamine levels. Clinical imaging studies have shown increased dopamine release in response to low-dose amphetamine in patients with schizophrenia, correlating with symptom exacerbation and elevated striatal dopamine synthesis capacity. Conversely, reduced dopamine release in mesocortical pathways, especially to the dorsolateral prefrontal cortex, is linked to cognitive impairments observed in the disorder (Sonnenschein et al., 2020). The inhibition of DAT protein helps increase dopamine in the synaptic cleft of neurons, which further increases phasic dopamine and causes autoinhibition in the presynaptic part of the neuron. The D2S receptor would be activated by phasic dopamine, which lowers the excitability of neurons (Amato et al., 2019). By the inhibition of DAT protein, cognitive impairments in schizophrenic patients and other neuropsychiatric illnesses can be treated (Gong et al., 2017). Currently, it is considered a promising target for the treatment of cognitive effects, and at the same time, inhibition of the DAT is important for patients who develop resistance to antipsychotic drugs such as Clozapine, Risperidone, Cariprazine, etc. The therapeutic benefits of DAT inhibitors such as amphetamine and methylphenidate are well-documented. However, they have limitations, including appetite suppression, the potential for abuse, variability in individual responses due to genetic factors, and alterations in neurotransmitter dynamics (Faraone, 2018). These drawbacks highlight the need for tailored treatment strategies and further research into alternative options for DAT inhibitors.

At present, several medications are made from traditional medicinal plants. They are crucial for meeting basic healthcare requirements in economically developing countries and have established themselves as a standard for protecting health. Plant-derived compounds offer several advantages over synthetic alternatives in drug discovery. They have lower toxicity, better safety profiles, and are a rich source of structural diversity (Martins and Brijesh, 2018; Muhammad et al., 2021). Medicinal plants have numerous phytochemicals in different parts that, as herbal medicines, can help cure neuropsychiatric disorders like schizophrenia and psychosis (Elkins et al., 2005). Most of the active metabolites used as medicines have natural sources, while thirty percent of novel chemical metabolites utilized as medications are entirely synthetic (Uddin and Zidorn, 2020). Aftimoon, Halayla, Brahmi, Bisfyij (Waseem et al., 2020), and Cannabis (Stasiłowicz et al., 2021) are secondary metabolites that are extracted from different medicinal plants and have been used for the treatment of schizophrenia.

In this study, inhibitors of DAT were searched out from the phytochemicals of six medicinal plants: Piper *retrofractum Vahl, Euphorbia neriifolia* L.*, Thevetia peruviana (*Pers) K. *Schum, Ficus hirta Vahl*, *Vitex negundo* L., and *Datura metel* L. (Ahmed and Kabidul Azam, 2014). These plants have been used by traditional medical practitioners for the treatment of schizophrenia-like psychotic episodes (Ahmed and Kabidul Azam, 2014). The selected inhibitors were screened through molecular docking and mode of binding interactions and were further evaluated for their physiological bioactivity, drug-likeness, oral bioavailability, and pharmacokinetic properties. The potently selected metabolites were studied to indicate their stability with the target protein binding pocket by molecular dynamic simulation analysis, pharmacophore analysis, and pharmacokinetic simulations. These parameters are essential in medicinal chemistry and drug design as they help assess the therapeutic potential of natural products by predicting their efficacy, safety, and suitability for oral administration. Moreover, such techniques accelerate the identification of promising compounds and reduce the need for extensive *in vitro* and *in vivo* experimentation. The novel inhibitors of the DAT reported in this study are potential candidates to inhibit DAT, target dopamine dysregulation, and can be experimentally validated as antipsychotic drug candidates for schizophrenia.

## Material and methods

### Selection of drug target

The three-dimensional structure of the dopamine transporter protein (DAT) (PDB ID: 4M48) was retrieved from the Protein Data Bank. The structure of the target protein has been resolved using X-ray diffraction to a resolution of 2.96 Å. The specific structure used in our study was selected due to its high resolution and suitability for studying ligand interactions. It consists of 543 amino acids and is associated with ligands including cholesterol, nortriptyline, chloride, and sodium ions.

### Selection of library

To construct a library of phytochemicals for screening potential antischizophrenic agents, six distinct medicinal plants, including *Piper retrofractum Vahl (* Supplementary Table S1 *), E. neriifolia* L. (Supplementary Table S2), *T. peruviana* (Pers) K. Schum (Supplementary Table S3), *F. hirta* Vahl (Supplementary Table S4), *V. negundo* L. (Supplementary Table S5), and *D. metel* L. (Supplementary Table S6), previously reported for their potential against neurological disorders were selected. Whereas, 107 phytochemicals were obtained from *T. peruviana (Pers) K. Schum*, 99 from *F. hirta Vahl*, 148 from *E. neriifolia L*., 200 from *V. negundo L*., 197 from *D. metel L*., and 239 from *Piper retrofractum Vahl*. A total of 990 metabolites were obtained from these selected medicinal plants and subjected to library preparation against the receptor protein.

### Target preparation and active site prediction

As performed in our previous studies (Shah et al., 2021; 2024), in Molecular Operating Environment (MOE) software, the protein structure was prepared for computational analysis by first removing all water molecules and detaching all attached ligands, ensuring a clear representation of the protein’s active sites. This was followed by energy minimization, a crucial step to alleviate any structural strains and achieve a more stable conformation. The protein was protonated by default parameters, adjusting hydrogen atoms per its amino acids. Protonation is essential for accurately modeling the protein’s electrostatic properties and interactions in biological systems. The MOE site finder tool was used to predict the active site of the target protein (PDB ID: 4M48) based on the amino acids’ size, number, and location in the protein binding site. The site finder tool helped predict a binding pocket that is important for the interaction between ligand and protein. During active site determination, dummy atoms were applied to the active site residue at the alpha center of the protein sphere (Shah et al., 2021).

### Preparation of ligand molecules

ChemDraw Ultra 12 software was used to generate 2D conformations of the phytochemicals that were retrieved as SMILES (simplified molecular input line entry system) from the PubChem database or collected from a meticulous literature survey and saved in Mol format. These metabolites were imported into MOE to create a database, followed by the generation of 3D conformers. To get the stable configuration of all 990 metabolites, their energy was minimized and partial charges were added to them as per previous practice.

Lumateperone was selected as the standard drug for comparison due to its unique pharmacological profile, which includes simultaneous modulation of serotonin, dopamine, and glutamate pathways. It is an FDA-approved treatment for schizophrenia (Maini et al., 2021). Lumateperone was also prepared along with the ligands.

### Validation of docking protocol

To ensure the accuracy and reliability of the docking protocol, a systematic validation procedure was employed, comprising docking and superimposition methods. Using MOE software, the reference drug Nortriptyline, co-crystallized with DAT (PDB ID: 4M48), was extracted from the complex and docked into the designated active site of DAT. The docking results were validated by superimposing the docked pose with the original crystallographic pose of Nortriptyline. The Root Mean Square Deviation (RMSD) of ≤2 Å or 0.2 nm indicates a reliable docking procedure.

### Molecular docking

Molecular docking was performed to identify lead metabolites with high binding affinity or scoring function, visualize them, and analyze their interactions with the receptor. The MOE (Molecular Operating Environment, 2022.02 Chemical Computing Group ULC, 1010 Sherbooke St. West, Suite #910, Montreal, QC, Canada, H3A 2R7, MOE2022. v11.18.1). MOE Dock tool was employed for molecular docking analyses (Khalid et al., 2024a). It helps to analyze the binding mechanism of the ligand to the receptor. Ligand binds with protein in different poses to get a stable configuration. The more stable the interaction of the ligand with the protein, the lower the value of the scoring function. Docking score values of ligand molecules were compared with the standard compound (Lumateperone).

### Visualization of molecular interactions

The ligand interaction tool of the MOE software was employed to elucidate the interactions between the ligand and DAT. This analysis provided detailed information about the bond types, distances, and energies, as captured in the ligand interaction reports. Complementing this, PyMOL was utilized for an in-depth visualization of these ligand-protein interactions. As reported (Khalid et al., 2024b), the 3D visualizations performed in PyMOL particularly focus on the protein-ligand binding pockets, offering a comprehensive analysis of their structural and interactional dynamics.

### Molecular dynamics simulations

MD simulations were conducted to explore the dynamic interactions of three amides, namely, chenoalbicin, dipiperamide G (a cinnamic acid amide alkaloid), and lyciumamide C (a phenolic amide), with the DAT protein. Each amide-protein complex was simulated for 100 ns to evaluate stability and interaction patterns.

Partial atomic charges for the ligands were assigned using the antechamber module of AMBER 20, (Arantes et al., 2021), as per previous practice (Khalid et al., 2024b). The Leap module was employed to add missing hydrogens, neutralize the system, solvate the complexes, and generate parameter and coordinate files for the simulation system. The ff14SB force field was applied to the protein, while the generalized Amber force field (GAFF) was used for the ligands. Protonated proteins were neutralized using counterions (Cl⁻ or Na⁺), and complexes were solvated in an octahedral TIP3P water box with a margin of 10.0 Å. The solvated complexes were saved in PDB format, and the parameter and coordinate files were prepared using the Leap module.

Before running the MD, the minimization process was carried out in three steps to resolve steric clashes. Initially, ions and the solvated water system were optimized, followed by the optimization of pocket residues, including backbone amino acids. Finally, the entire system was optimized to ensure the relaxation of the protein-ligand complexes. Each minimization step comprised 2500 steps of the steepest descent method followed by 5000 steps of the conjugate gradient method. After minimization, the system was gradually heated from 0 to 300 K, and equilibration steps were performed at 300 K using Langevin dynamics with a collision frequency of 1 ps⁻^1^ and a force constant of 10 kcal mol⁻^1^ Å⁻^2^. The MD production phase was executed under the NPT (constant number of particles, pressure, and temperature) ensemble at 300 K and 1 atm pressure for 100 ns.

The trajectories generated during the simulations were analyzed to evaluate RMSD, RMSF (Root Mean Square Fluctuation), radius of gyration (Rg), Solvent Accessible Surface Area (SASA), cross-correlation, principal component analysis (PCA), and free energy landscape (FEL) (Kitao, 2022; Palma and Pierdominici-Sottile, 2023). The detailed analysis of hydrogen bonding throughout the simulation was also performed. These analyses provided insights into the dynamic stability, conformational flexibility, and binding behavior of the complexes.

### Binding free energy calculations

Binding free energy calculations were performed using MMPBSA (Molecular Mechanics Poisson-Boltzmann Surface Area)/MMGBSA (Molecular Mechanics Generalized Born Surface Area) approaches implemented in AMBER 20 (Khalid et al., 2024b). Snapshots were extracted from the last 20 ns of the MD simulations, totaling 1000 snapshots for each complex. The calculations included molecular mechanics energy (ΔEmm), solvation-free energy (ΔGsol), and entropy (TΔS). Molecular mechanics energy components were further dissected into van der Waals (ΔEvdW), non-bonded electrostatic (ΔEele), and solvation energy contributions, including polar (ΔGele, sol) and nonpolar (ΔGnonpolar, sol) components.

### Pharmacophore features analysis

After performing docking and interaction analysis of the top three amides and their metabolites, pharmacophoric analysis was conducted to identify the key features responsible for binding affinity and interaction profiles (Ahmad et al., 2024). The analysis focused on the pharmacophoric features observed in the interaction profiles of the compounds, highlighting those that formed consistent bonding interactions with the active site of the target protein. Using the MOE Pharmacophore Query Editor, pharmacophoric features such as hydrogen bond donors, hydrogen bond acceptors, hydrophobic features, aromatic features, and exclusion volumes were identified and validated.

### Assessment of drug-likeness properties

The physicochemical properties and drug-likeness properties of metabolites were studied using SwissADME. This web server helps to evaluate the metabolites based on different properties like molecular weight, logP, number of rotatable bonds, number of acceptor atoms, number of H-bond donors, number of violations, and total polar surface area. To predict the drug-likeness properties of metabolites, different rules like Lipinski’s rule (Lipinski et al., 1997), Veber’s rule (Veber et al., 2002), Egan’s rule (Egan et al., 2000), and Muegge’s rule (Muegge et al., 2001) were studied. According to Lipinski’s rule, the number of H-bond acceptors in a ligand should be equal to or less than 10. The molecular weight of the ligand should be less than five hundred Daltons, and the number of H-bond donors in the ligand should be equal to or less than 5. According to the Veber filter, ligands should have a total polar surface area equal to or greater than 140, and the number of rotatable bonds should be equal to or greater than 10 (Sharma et al., 2020). According to Egan’s rule, ligands should have a topological polar surface area (TPSA) of 140 Å^2^ or less and a log P value (lipophilicity) between −1 and 6 to ensure oral bioavailability (Egan et al., 2000). Muegge’s rule evaluates drug-likeness by considering molecular properties, including a molecular weight of 200–600 Da, a logP value between −2 and 5, 1–15 rotatable bonds, and a total polar surface area less than 150 Å^2^. These parameters help predict the potential of a compound to exhibit favorable pharmacokinetic profiles and bioavailability (Muegge et al., 2001).

### Bioactivity analysis

The bioactivity score encapsulates a drug candidate’s likelihood of being an effective pharmaceutical agent (Yamin et al., 2024). It serves as a critical indicator, quantifying the potential success of the drug in interacting with biological targets, which is essential in the drug development process. Molinspiration online web server was used to evaluate different properties like bioactivity score, nuclear receptor ligand, enzyme inhibitor, G protein-coupled receptors (GPCR) ligand, and ion channel modulator that helped identify the leading anti-schizophrenic agent.

### Pharmacokinetic profiling

Before being refined and developed as therapeutic candidates, top metabolites must pass through essential characteristics and phases known as pharmacokinetic or ADMET (absorption, distribution, metabolism, excretion, and toxicity) qualities (Yamin et al., 2024). ADMET properties of metabolites were analyzed by different tools. Swiss ADME (http://www.swissadme.ch/index.php), ADMETlab 3.0, pkCSM (http://biosig.unimelb.edu.au/pkcsm/prediction), and AdmetSAR 2.0 (http://lmmd.ecust.edu.cn/admetsar2) are free online web tools that are used for the study of the properties of metabolites.

### Pharmacokinetic simulation

The focus was on predicting the plasma concentration-time profile of shortlisted metabolites, namely, Chenoalbicin, Dipiperamide G, and lyciumamide C in humans combining their potential to inhibit the DAT (SLC6A3) and their ADMET profile. Physiologically-based pharmacokinetic (PBPK) modeling was performed using PK-Sim software to predict the brain interstitial unbound concentration-time profiles of the metabolites.

### Target and population

The brain was selected as the target organ, with a focus on DAT (SLC6A3) as the primary target. DAT was selected as the target due to its promising potential in treating the cognitive symptoms of schizophrenia. A virtual population of n = 100 individuals (50% female) aged 25–35 years was simulated by Pk-Sim, representing the target demographic for schizophrenia treatment, as this age group is most commonly affected by schizophrenia (Holm et al., 2021). 100 individuals are commonly used in similar PBPK modeling studies to capture a meaningful range of inter-individual variability (Zhuang & Lu, 2016).

### Administration protocol

The administration protocol was based on the FDA-approved Standard drug Lumateperone, used to treat schizophrenia. Oral administration of 42 mg single dose over 7 days. It has been used as an antihypertensive and antipsychotic drug and is relevant for treating schizophrenia (Maini et al., 2021).

### Simulation setup

**FIGURE 1:**
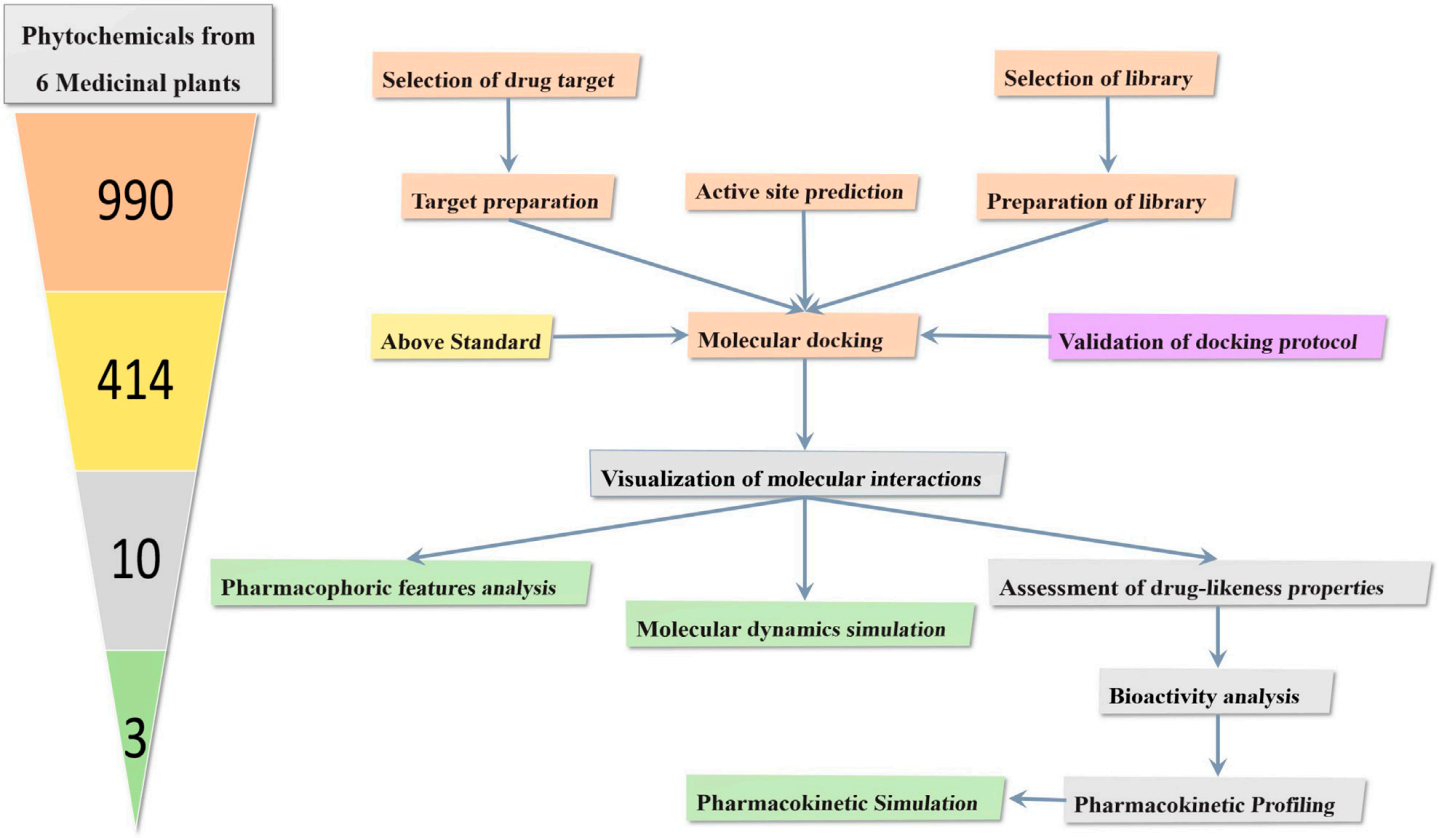
The workflow shows the screening of dopamine transporter inhibitors from six medicinal plants through molecular docking and evaluation for bioactivity, drug-likeness, and pharmacokinetic properties. Potent metabolites were further analyzed using molecular dynamics and pharmacophore analysis and pharmacokinetic simulations. The numbers in the cone, shaded in different colors, correspond to the flowchart, indicating how metabolites were shortlisted and studied at each stage.

The PBPK modeling was performed using a standard model for small molecules in the Brain (unbound). Simulation Parameters: The physicochemical properties of metabolites including molecular weight, lipophilicity, plasma protein binding, pKa, and solubility, were taken from ChemAxon’s Chemicalize software, and used as input into PK-Sim. CYP3A4 was set as the primary metabolizing enzyme for all compounds (Willmann et al., 2012; Frechen et al., 2021). The visual summary of the overall steps involved in the study is presented in Figure 1.

## Results and discussion

### Finding and analyzing the active site

**FIGURE 2:**
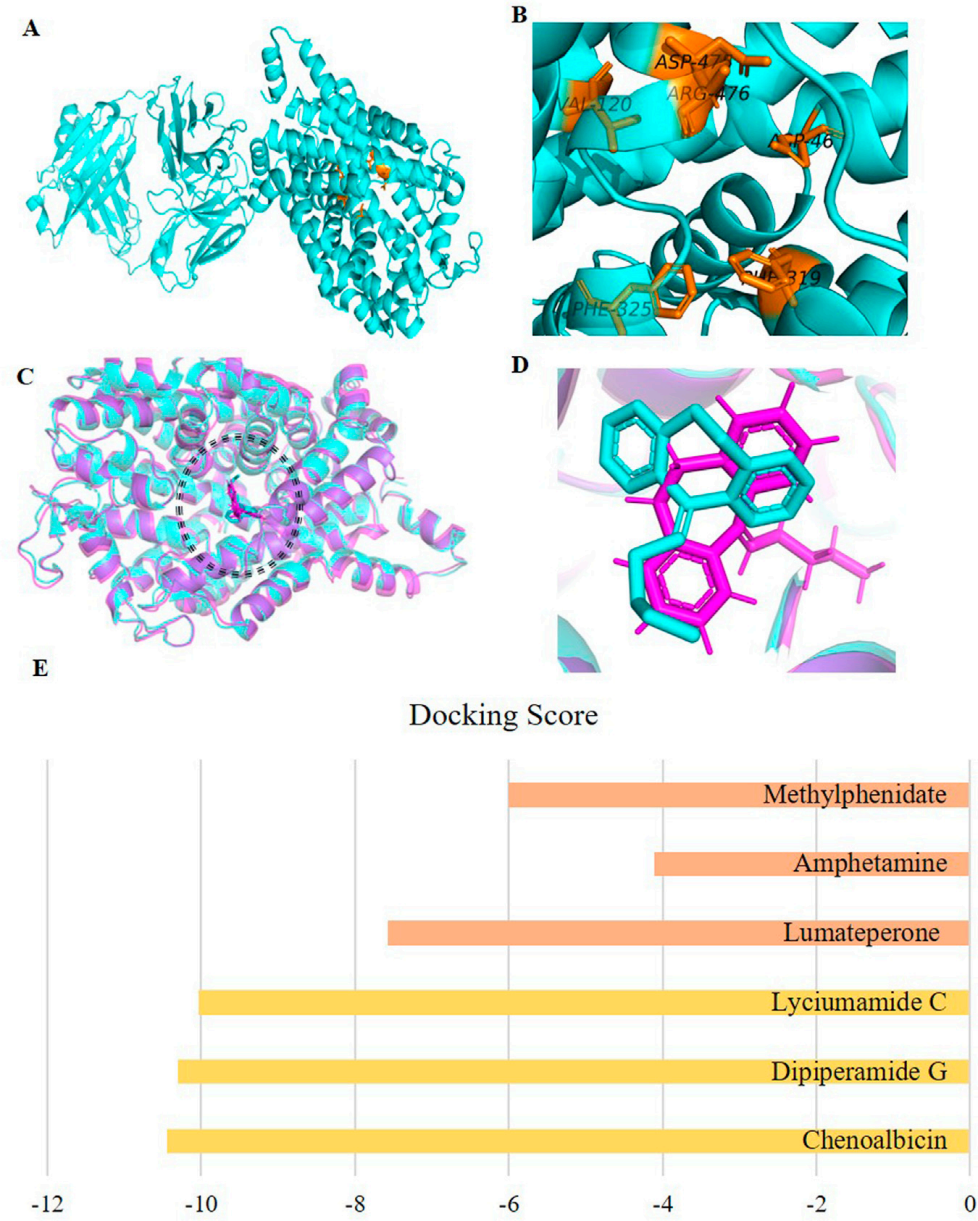
**(A)** 3D view of the Dopamine transporter protein (cyan) with the active site region in chain 1 (alpha chain) highlighted in orange, **(B)** Labeled active site residues **(C)** Superimposed docked (cyan) and crystallographic (purple) complexes of the co-crystalized ligand (Nortriptyline) in the active site of protein (showing only alpha chain which has active site), visualized in PyMOL, to validate the docking protocol. **(D)** Zoomed-in view of the docked (cyan) and crystallographic (purple) Nortriptyline molecules. **(E)** Comparison of the docking scores of the top three hits (Yellow) from our study with those of existing drugs for schizophrenia, including amphetamine, methylphenidate, and lumateperone (Orange).

According to the site finder tool of MOE, the potential active site of DAT had the following amino acid residues: PHE43, ASP46, LEU47, ALA48, TRP51, ARG52, LEU56, VAL120, TYR123, TYR124, ILE127, ASP312, THR315, GLN316, PHE319, SER320, LEU321, GLY322, PHE325, GLU384, GLY385, PRO386, SER421, PHE468, PHE471, HIS472, ASP475, ARG476, TYR477, ALA479, GLY480, TYR481, LEU538, GLY541, TYR542, GLU543. Moreover, this active site was selected based on amino acid residues documented in the literature and recognized for their involvement in various interactions. These residues were validated as key participants in the protein’s binding activity, highlighting their functional importance. The significant and frequently reported active site residues of DAT are presented in Figures 2A, B. Interactions with Ser320 and Ser421 have been observed in the case of dopamine binding to the DAT (Reith et al., 2015). Furthermore, Residues such as Asp475 and Phe319, are consistent with key binding interactions reported in the literature for DAT inhibitors (Loland, 2015; Nepal et al., 2023).

In analyzing the amino acid composition, it’s notable that while there is a balance of both hydrophobic and hydrophilic amino acids, the hydrophobic ones, such as phenylalanine (Phe) with 5 repetitions, tyrosine (Tyr) with 5 repetitions, and leucine (Leu) with 4, is repeated more frequently. This suggests a significant presence of hydrophobic regions within the protein, which could be involved in interactions with hydrophobic molecules or within membrane environments (Payandeh and Volgraf, 2021). Conversely, hydrophilic amino acids, though present in a variety, are less frequent in comparison. These include aspartic acid (Asp) with 3 repetitions, glutamic acid (Glu) and arginine (Arg) each with 2, and serine (Ser) with 2, along with a single occurrence of histidine (His) (Payandeh and Volgraf, 2021). This diverse but less frequent presence of hydrophilic residues suggests potential sites for various protein-protein or protein-ligand interactions in aqueous environments, but these interactions might be less predominant than the hydrophobic interactions suggested by the more frequent hydrophobic residues.

### Validation of docking protocol

Two complexes were prepared, one reference complex downloaded from PDB ID: 4M48, which included the co-crystallized ligand Nortriptyline, and the other, our docked complex with the same ligand. Both complexes were aligned using PyMOL, with almost all atoms from each complex aligned with each other (Figures 2C, D), indicating perfect alignment. This RMSD score of 0.8 Å, being in the ideal range (≤2 Å), confirms the validity of our docking procedure (Ahmad et al., 2024).

### Binding affinity of the ligands

Docking results of the database with the DAT showed that out of 990, 414 phytochemicals showed better binding affinity than the standard; lumateperone (−7.57 kcal/mol) and the existing DAT inhibitors; amphetamine (−4.11 kcal/mol) and methylphenidate (−6.00 kcal/mol) (Supplementary Tables S1–S6; Figure 2E). This suggests that a significant portion of the screened metabolites have the potential to inhibit the DAT, an essential target in treating schizophrenia. Such strong binding affinities indicate the feasibility of natural compounds as therapeutic agents.

The top ten metabolites were screened for further analysis based on their stronger binding affinities and favorable docking values. The top ten metabolites include chenoalbicin, dipiperamide G, nigramide R, chabamide G, dipiperamide F, 3,12-di-O-acetyl-8-O-tigloylingol, 2,4-imidazolidinedione, and 2,4-Imidazolidinedione,5-[3,4-bis[(trimethylsily)oxy]phenyl]-3-methyl-5-phenyl-1-(trimethylsilyl), Lyciumamide C, Chabamide, and Dipiperamide E. The range of docking values of these phytochemicals was from −10.45 kcal/mol to −9.79 kcal/mol (Supplementary Table S7), and they had less binding energy as compared to standard compound. Importantly, these values reflect not only favorable interactions with DAT but also suggest that these metabolites may provide greater efficacy than the standard drug. The binding energy values of these metabolites also suggest their potential ability to stabilize the active site of DAT through critical hydrogen bonding and hydrophobic interactions, as detailed later.

These selected metabolites were advanced to further stages of analysis. This included a thorough assessment of their drug-like properties and pharmacokinetic profiles, which reflect their potential behavior and efficacy within a biological system when used as therapeutic agents.

### Deciphering protein-ligand interaction patterns

**FIGURE 3:**
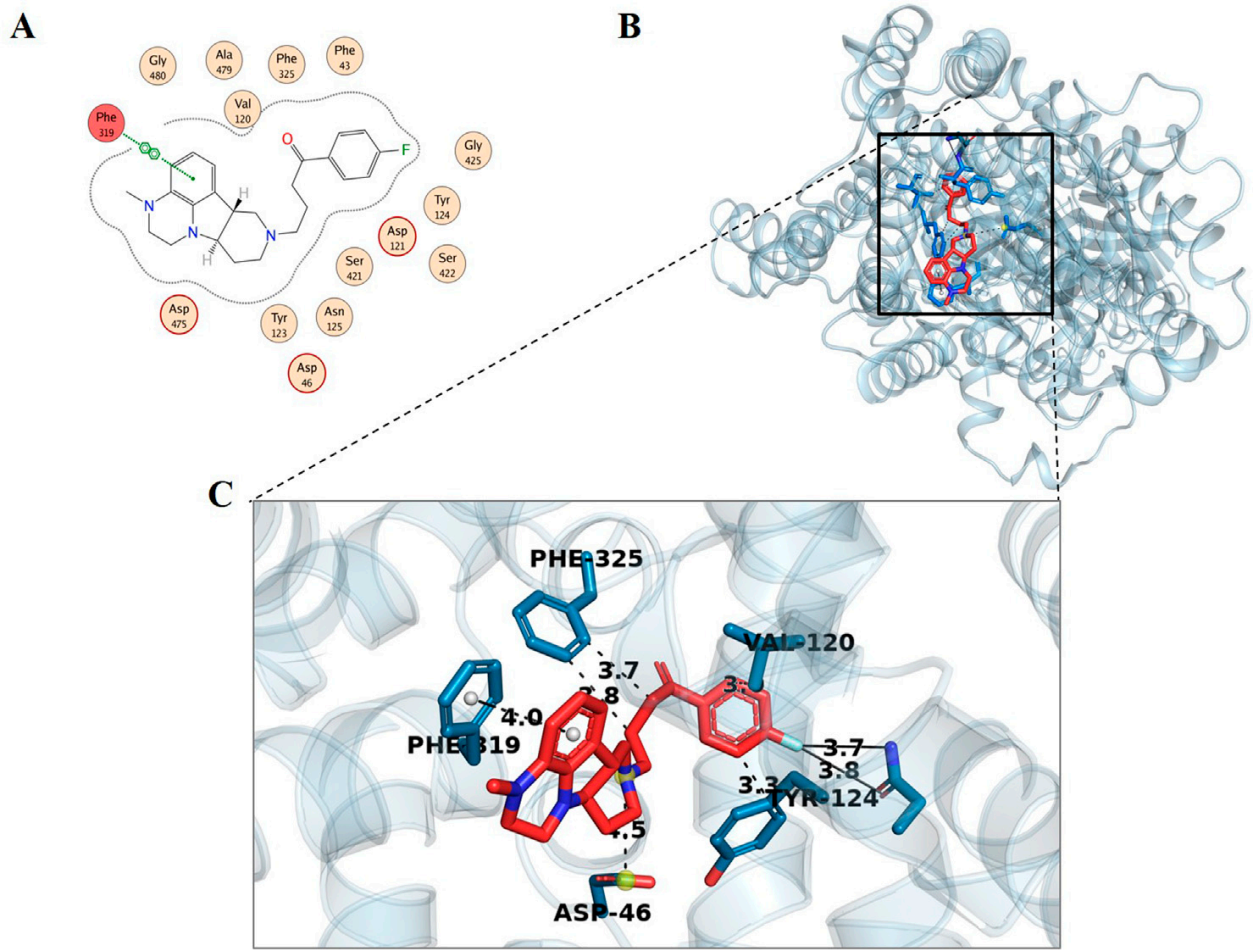
Docking interaction analysis of standard compound (Lumateperone); 2D interaction map **(A)** with red indicating hydrogen-bonded residues, and 3D conformation **(B, C)** in the binding pocket of dopamine transporter protein. In the 3D conformation, hydrophobic interactions are observed with Val120 (2.99 Å), Tyr124 (3.32 Å), and Phe325 (3.85 Å and 3.67 Å). π-Stacking interactions occur with Phe319 (4.00 Å), while halogen bonds are formed with Asn125 (3.85 Å and 3.70 Å). Additionally, a salt bridge is identified with Asp46 (4.53 Å).

For the analysis of binding modes of interactions between DAT and the identified top ten metabolites, 2D and 3D interactions of the metabolites were studied with MOE and PyMOL, respectively. Although the discussion is more focused on the results obtained from MOE only several types of bonding, i.e., hydrogen bonding, hydrophobic interactions, bond length, and bond energy of phytochemicals were analyzed (Supplementary Table S7). It should be noted that DAT contains three chains, one alpha-helix, and two beta sheets. The active site was located on the alpha helix therefore only the first chain is shown in Figures 2A, B. The interaction of the standard compound was also observed (Figure 3).

**FIGURE 4:**
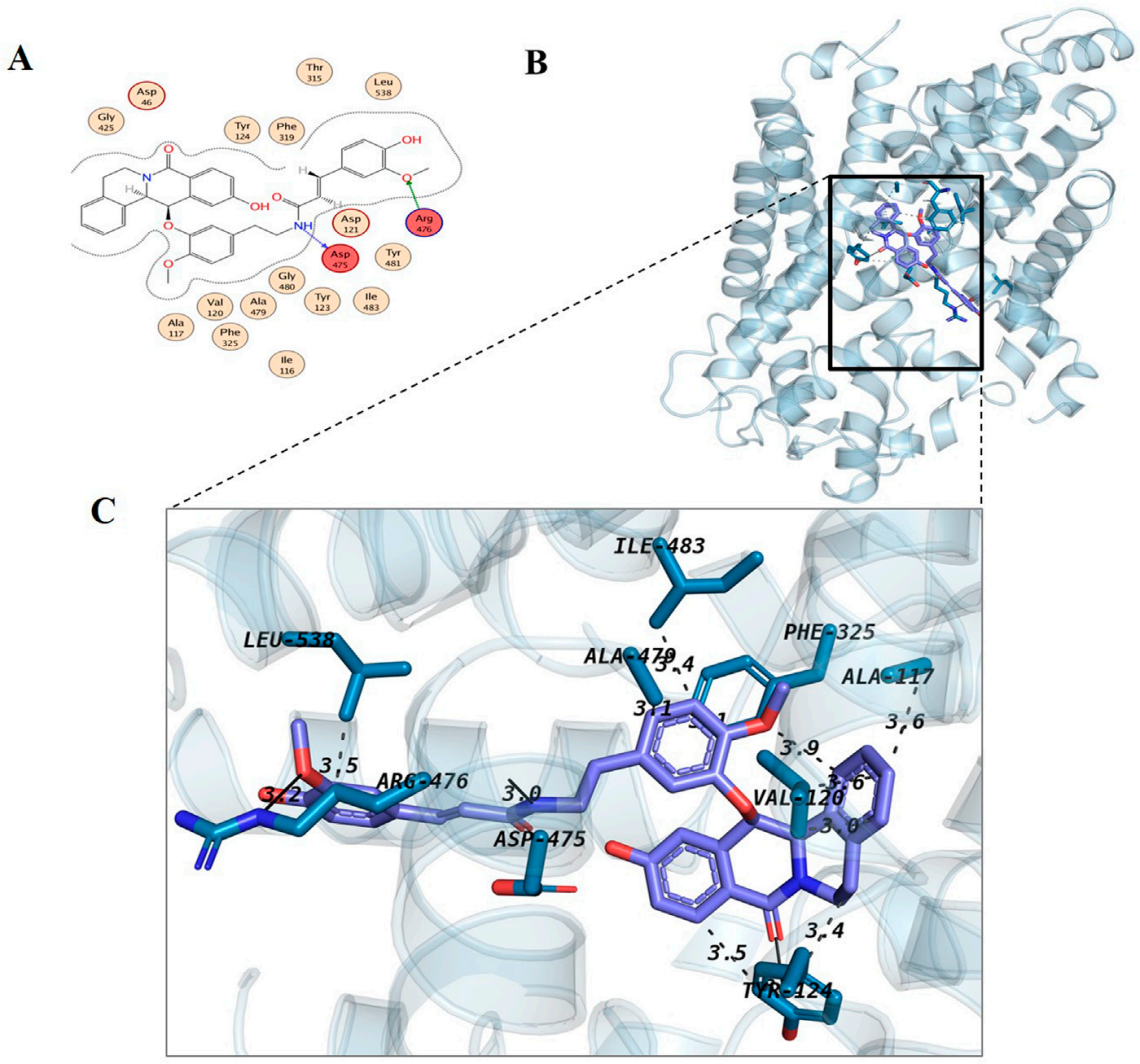
Docking interaction analysis of metabolite 1 (Chenoalbicin); 2D interaction map **(A)** with red indicating hydrogen-bonded residues and 3D conformation **(B, C)** in the binding pocket of dopamine transporter protein. The 3D interaction diagram of Chenoalbicin with the DAT revealed several key interactions. Hydrophobic interactions are formed with Ala117 (3.60 Å), Val120 (2.98 Å and 3.62 Å), Tyr124 (3.55 Å and 3.41 Å), Phe325 (3.95 Å and 3.09 Å), Ala479 (3.11 Å), Ile483 (3.41 Å), and Leu538 (3.46 Å). Hydrogen bonds are observed with Tyr124 (2.82 Å), Asp475 (2.05 Å), Arg476 (2.29 Å), and Asn213 (3.20 Å).

Metabolite 1, Chenoalbicin formed two hydrogen bonds (Asp475-H donor, Arg476-H acceptor) and most of the hydrophobic interactions (Tyr124, Phe319, Asp46) with DAT (Figure 4). The details of the bonding distances from the MOE results are provided in Supplementary Table S7. Chenoalbicin is a cinnamic acid amide alkaloid from the roots of *Chenopodium album* (Cutillo et al., 2004) and such alkaloids attached to cinnamic acid amides are reported as allelopathic chemicals (Cutillo et al., 2003). Metabolite 2, Dipiperamide G showed hydrogen bonding with Asp475 and Phe325 that act as H-donor and pi-pi interactions, respectively (Supplementary Figure S1). It showed hydrophobic interactions with Tyr123, Val120, Ile483, and Ala117. Among our top metabolites, Dipiperamide E, Dipiperamide F, and Dipiperamide G are amides and were isolated from the fruits of *Piper retrofractum.* Several biological activities have been explored from this fruit extract, such as antibacterial, antifungal, cytotoxic, insecticidal, anti-obesity, and antidiabetic (Muharini et al., 2015).

Metabolite 8, Lyciumamide C exhibited two hydrogen bonds with Asp46 (H-donor) and Phe319 (H-pi) and hydrophobic interactions with Tyr123, Val120, Ser320, and Ala48 (Supplementary Figure S2). The interactions with Phe319 and Ser320 have been observed in the case of dopamine binding to DAT or the binding of DAT inhibitors. Blocking these crucial residues reduces the likelihood of dopamine binding to DAT, which serves as the primary objective of this research (Reith et al., 2015).

Lyciumamide C is a phenolic amide from the stem of *Lycium barbarum*. It is considered a therapeutic agent and was studied for its anticancer potential (Zhu et al., 2017). Metabolite 3, Nigramide R with DAT by forming two bonds with ARG476 and PHE325. These were pi-H and pi-pi interactions with bond distances of 4.17 and 3.99 Å, respectively (Supplementary Figure S3). It also presented variable hydrophobic interactions (Supplementary Table S7). Nigramide R was isolated from *Piper retrofractum* and reported as one of its most active constituents, potentially for its cytotoxicity among the amides of the fruit extract (Muharini et al., 2015).

Chabamide G (Metabolite 4), Dipiperamide F (Metabolite 5), and 2, 4-Imidazolidinedione, 5-[3,4-bis[(trimethylsily)oxy]phenyl]-3-methyl-5-phenyl-1-(trimethylsilyl) (Metabolite 7) showed hydrogen as well as hydrophobic interactions with the receptor protein (Supplementary Figures S4, S5, S7). Among them, chabamide G is isolated from the roots of *piper chaba hunter* (Rao et al., 2009) and is a potent anticancer dimeric alkaloid (Rao et al., 2011). Whereas 2, 4-Imidazolidinedione, 5-[3,4-bis[(trimethylsily)oxy]phenyl]-3-methyl-5-phenyl-1-(trimethylsilyl) an alkaloid, was isolated from the ethanolic leaf extract of *D. metel* (Chukwudoruo et al., 2022). Three metabolites, namely, 3,12-di-O-acetyl-8-O-tigloylingol (Metabolite 6), chabamide (Metabolite 9), and dipiperamide E (metabolite 10), interacted through one hydrogen bond with Asp46 and hydrophobic interactions with Phe319 and Val120, respectively (Supplementary Figures S6, S8, S9). 3,12-di-O-acetyl-8-O-tigloylingol is an ingol-type diterpene isolated from *Euphorbia antiquorum* L. (GEWALI et al., 1989). This metabolite has been previously reported for HIV-1 latency reactivating activity (Vasas and Hohmann, 2014). Similar to its structural analog, Chabamide G, Chabamide is a dimeric alkaloid and was first isolated from the stems of *Piper Chaba Hunter* and showed antimalarial potential (Rukachaisirikul et al., 2002).

The range of bond energy for these ten hits was from −2.3 to −0.0 kcal/mol. The range of their bond lengths was from 4.32 to 3.02 A^0^. Our standard interaction with one bond only resulted in Phe319 forming a weaker pi-pi stacking (Figure 3), whereas Lyciumamide C and Chabamide also interacted with the same residue but with stronger H-pi interactions. Residues such as Asp475 and Phe319, identified in this study, are consistent with key binding interactions reported in the literature for DAT inhibitors. (Loland, 2015; Nepal et al., 2023). Interactions with Ser320 and Ser421 have been observed in the case of dopamine binding to the DAT (Reith et al., 2015). This reinforces the biological significance of the identified metabolites. Moreover, all the hits presented a stronger interaction profile than the standard compound, and they have been reported before for various biological activities. The identified hits have a strong affinity with the active site of DAT by forming various hydrophobic and hydrophilic interactions, which suggests their potential role in the treatment of schizophrenia.

### Molecular dynamic mapping

For molecular dynamic simulations, AMBER 20 was employed, and the three amide complexes the chenoalbicin-DAT, the dipiperamide G-DAT, and the lyciumamide C-DAT complexes were subjected to simulation.

### Root mean square deviation (RMSD)

**FIGURE 5:**
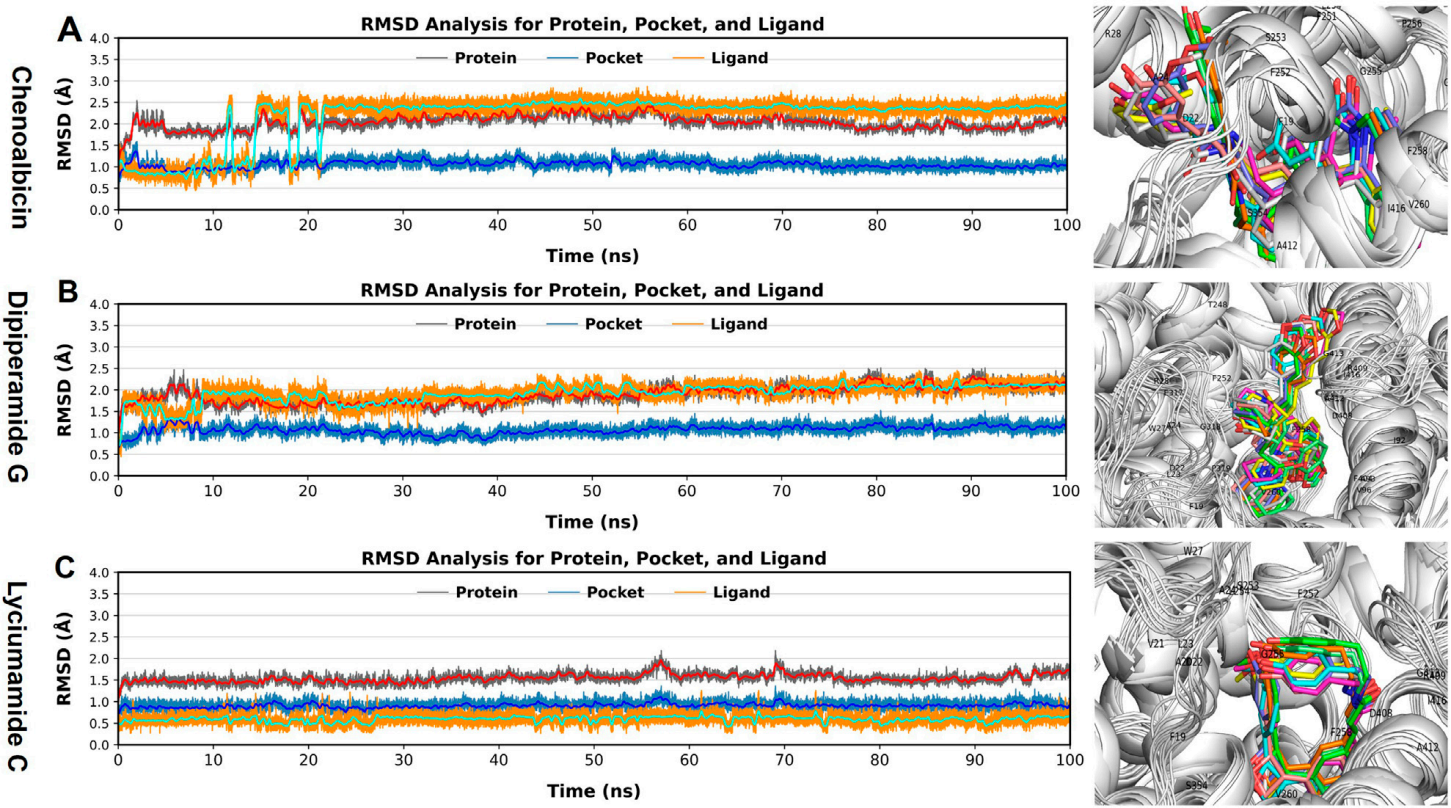
Root Mean Square Deviation (RMSD) analysis for **(A)** Chenoalbicin-DAT, **(B)** Dipiperamide G-DAT, and **(C)** Lyciumamide C-DAT complexes. RMSD values for protein (grey), pocket (blue), and ligand (orange) were monitored to assess structural stability across 100 ns of MD simulation. 3D representations of the ligand-DAT complexes, with snapshots at various intervels highlight different conformations of the ligands bound in the DAT pocket.

The reference frame backbone was aligned to each protein frame before the atom selection to compute the RMSD values. The RMSD in the bound and unbound states of the amides and protein was calculated and plotted against the protein’s Cα atoms as a histogram to assess the conformational stability and dynamic features from the initial configuration to the final state (Figure 5). The docked complex may be stable if there are slight deviations from the RMSD curve, and vice versa. In this case, the circumstances are the binding of the amides, namely, chenoalbicin, dipiperamide G, and lyciumamide C, with DAT.

In the results of the chenoalbicin-DAT complex, the RMSD for the DAT pocket remained stable at 1 Å during 100 ns simulation. The DAT backbone initially showed a deviation of 0.5 Å till 15 ns, reached at 2.5 Å then remained stable. For chenoalbicin, RMSD calculated started at 1.0, and after the minor deviations till 15 ns, it stabilized at 2.5 Å ± 0.5 Å. There were no major variations during 100 ns (Figure 5A). The RMSD plot of chenoalbicin was similar to the corresponding complex; no major variations were observed, and both ligand and protein complexes were stabilized after 15 ns. For the dipiperamide G-DAT complex, the RMSD for the DAT pocket remained stable at 1 Å ± 0.5 Å. The DAT backbone remained stable at 2 Å ± 0.5. The RMSD of the dipiperamide G showed a minor deviation of 2 Å before 10 ns then remained stable at 2 Å ± 0.5 and stayed aligned with the pocket. The structure of the DAT remains stable throughout the simulation, and the dipiperamide G showed no major deviation during 100 ns of the simulation (Figure 5B). The RMSD of the lyciumamide C-protein complexes and the corresponding ligand were fully aligned with no major variations; therefore, it showed the most stability among the other two. The lyciumamide C and DAT pocket remained stable at 0.5 ± 0.5 Å and 1.0 Å ± 0.5 Å, respectively. However, the DAT backbone showed minor deviations of 1.0 Å at 60 and 70ns (Figure 5C). These results can be observed from the snapshots taken during the simulations that show that all three ligands remained in the active site and both ligands and the DAT did not show major structural changes during the 100 ns simulation (Figure 5).

The RMSD results of all three amide complexes indicated their stability and further supported the strong and stable interactions observed in the docking analysis.

### Root mean square fluctuations

**FIGURE 6:**
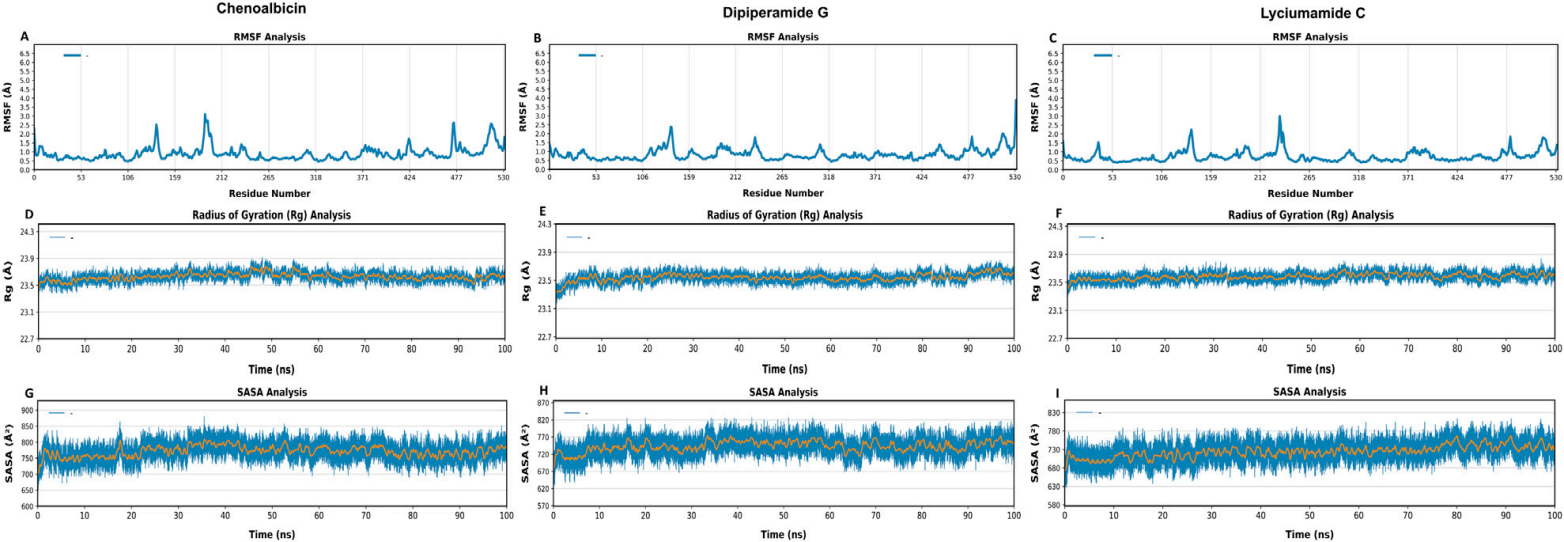
Root Mean-Square Fluctuation (RMSF) **(A–C)**, Radius of gyration (Rg) **(D–F)** and Solvent Accessible Surface Area (SASA) **(G–I)** analyses of DAT protein bound with Chenoalbicin, Dipiperamide G, and Lyciumamide C over the 100 ns simulation period. The x-axis represents residue numbers/time in ns, while the y-axis indicates the RMSF, Rg, (in Å) or SASA values (in Å^2^).

RMSF values represent the local energy variations in the protein chain. Peaks in the RMSF plots represent residues of the protein that fluctuates the most during the simulations. Additionally, proteins with a greater number of RMSF peaks have more flexible domains (Ghahremanian et al., 2022). To gain a better understanding of the stability of the complexes that were created, we conducted a residual flexibility study. The results highlighted that the three amides and protein complexes had lower values of flexibility (Figures 6A–C). Notably, while specific residue identities cannot be directly determined from these plots, the lower RMSF values observed in regions corresponding to the binding pocket suggest a stable interaction of the ligands with the protein. Conversely, regions with optimal fluctuations in RMSF values, such as peaks near residue indices ∼200 and ∼500, correspond to flexible loop regions or terminal residues. These areas likely do not directly influence ligand binding stability but reflect natural flexibility in non-binding regions of the protein. This pattern is consistent with the expected behavior of a structurally stable protein-ligand complex.

### Radius of gyration (Rg)

Rg was tracked for the three ligands-bound DAT systems during 100 ns of simulation to assess the effect of ligand binding on the structural compactness of DAT (Figure 6). Throughout the simulation, the Rg values for each system stayed stable, suggesting that the overall DAT structure remained compact with all three ligands present, namely, Chenoalbicin, dipiperamide G, and lyciumamide C (Figures 6D–F). The Rg values for the Chenoalbicin-DAT complex remained constant at 23.5 ± 0.5 Å (Figure 6D). A similar pattern was shown by the dipiperamide G, and lyciumamide C-DAT complexes, with Rg values falling at 23.5 ± 0.5 Å throughout the simulation (Figures 6E, F). All of these variants showed stable structural integrity and stayed within reasonable bounds. To preserve the structure necessary for a successful ligand interaction, DAT must remain in its initial folded state, which is implied by its stability.

### Solvent accessible surface area (SASA)

SASA was monitored for 100 ns of simulation for the three ligand-protein systems to investigate the effect of ligand binding on the solvent accessibility of DAT protein (Figures 6G–I). SASA offers information on the extent of solvent exposure to the protein surface, indicating potential structural alterations or differences in compactness brought on by ligands.

The range of SASA variation for the Chenoalbicin-DAT complex was 700 Å^2^ to 800 Å^2^. These numbers show consistent solvent accessibility over the course of the simulation and fall within the allowed range (Figure 6G). The dipiperamide G-DAT complex also displayed SASA values within a similar range, suggesting minimal variations in the structure of DAT protein (between 670 Å^2^ and 770 Å^2^) brought on by the solvent’s action (Figure 6H). The stable SASA values, which varied between 680 Å^2^ and 780 Å^2^, were likewise maintained by the lyciumamide C-DAT complex, indicating that the system’s surface area exposed to the solvent was comparable (Figure 6I). The three complexes’ similar SASA patterns show that neither the overall folding nor the surface exposure of the protein was significantly altered by ligand binding.

### Binding free energy

**FIGURE 7:**
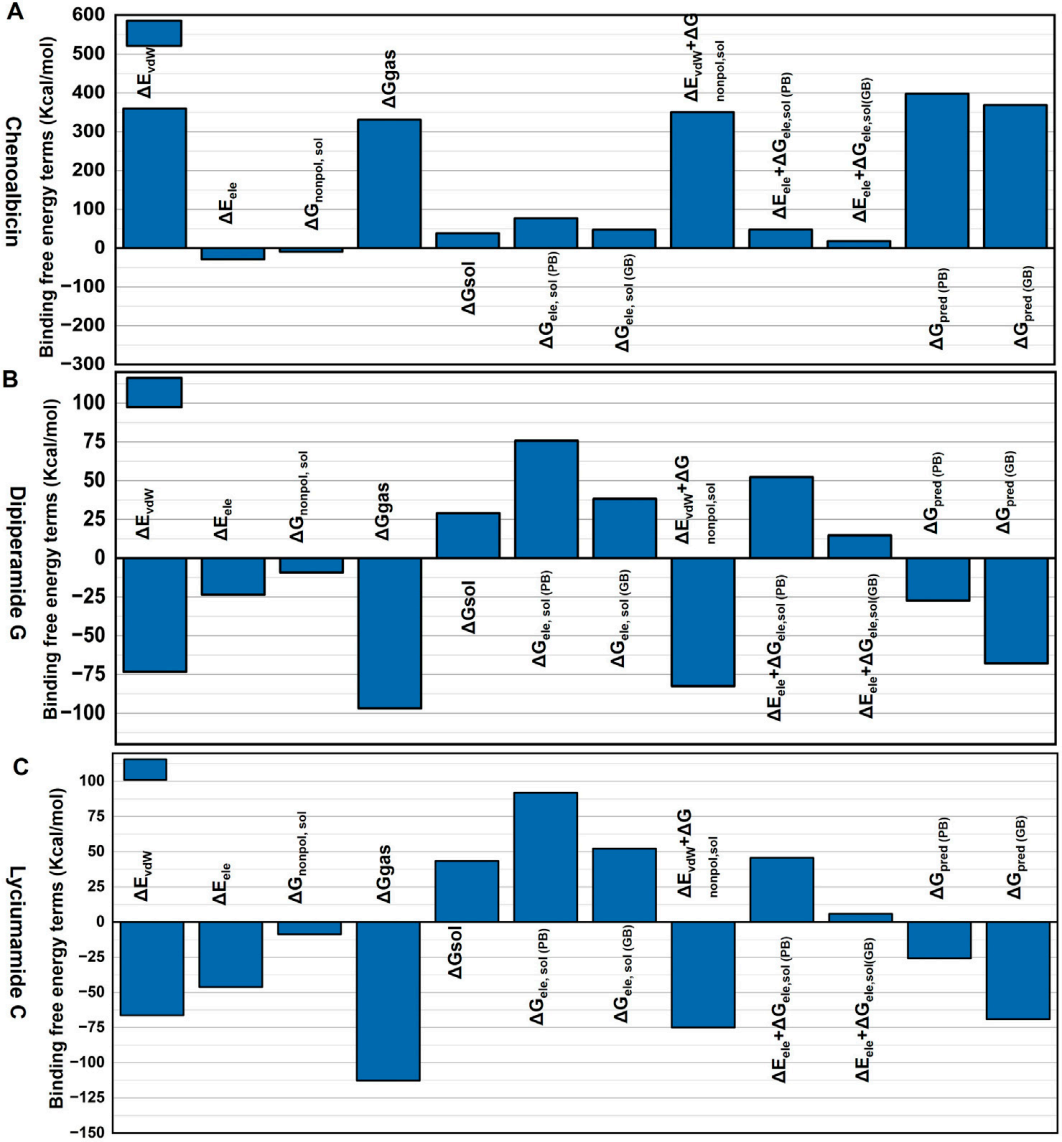
Binding free energy decomposition analysis for the **(A)** Chenoalbicin-DAT, **(B)** Dipiperamide G-DAT, and **(C)** Lyciumamide C-DAT complexes. The energy terms analyzed include van der Waals energy (ΔEvdw), electrostatic energy (ΔEele), nonpolar solvation free energy (ΔGnonpolar, sol), gas-phase binding energy (ΔGgas), solvation free energy (ΔGsol), and electrostatic solvation free energy calculated using the Poisson-Boltzmann (ΔGele, sol (PB)) and Generalized Born (ΔGele, sol (GB)) models. Additionally, combined energy contributions such as van der Waals energy with solvation energy (ΔEvdw + ΔG) and predicted binding free energies (ΔGpred (PB) and ΔGpred (GB)) are presented.

The binding free energy results of the Chenoalbicin-DAT complex (Figure 7A) showed high gas-phase energy (ΔEvdw and ΔGgas), indicating strong hydrophobic (nonpolar) interactions. Moreover, the solvation energy (ΔGsol) is unfavorable, especially in polar solvents, but contributes slightly to binding stability. ΔGpred for both PB and GB suggested moderate binding affinity. Collectively, Chenoalbicin-DAT (Figure 7A) shows the highest nonpolar interaction but it might need optimization due to the unfavorable electrostatic and solvation energies. The Dipiperamide G-DAT complex displayed almost balanced ΔEvdw and ΔGgas, indicating moderate nonpolar interactions. In contrast, the contribution of ΔEele was higher than the Chenoalbicin-DAT complex, indicating more contribution of electrostatic interactions for the stabilization of Dipiperamide G in the binding pocket of DAT protein. ΔGsol is more contributing in PB compared to GB, indicating improved binding. ΔGpred (PB) indicates good binding with GB, although significantly lower in PB prediction, supported stability. Overall, the Dipiperamide G-DAT (Figure 7B) balances the energy. The Lyciumamide C-DAT complex (Figure 7C) displayed the highest value of ΔGgas compared to the other two, which suggested strong nonpolar binding. Though less than ΔGgas, its ΔEvdw and ΔEele values also significantly contributed. ΔGpred (PB and GB) values are comparable with the results of the Dipiperamide G-DAT complex; these values were favorable, suggesting this complex has the overall binding affinity. Collectively, Lyciumamide C-DAT demonstrates the best binding potential due to favorable energy terms in both gas and solvation phases.

The results indicate that the Lyciumamide C-DAT complex exhibits favorable binding characteristics, with strong interactions and improved performance in solvation environments. In contrast, the Chenoalbicin-DAT and Dipiperamide G-DAT complexes demonstrate comparatively weaker binding affinities, likely due to less favorable energy contributions in solvation and gas-phase interactions.

### Hydrogen bond dynamics over time

**FIGURE 8:**
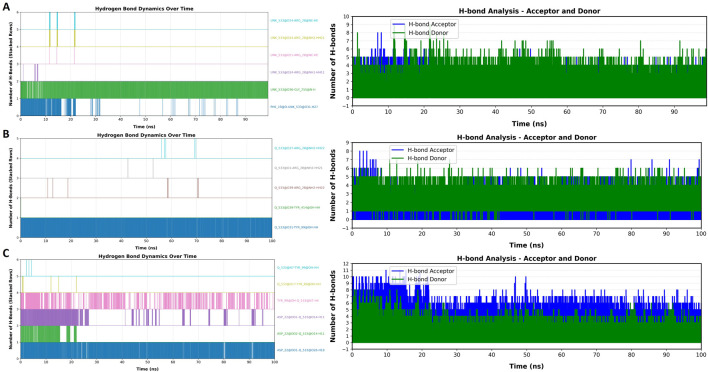
Hydrogen bond dynamics over time for the three compounds **(A)** Chenoalbicin, **(B)** Dipiperamide G, and **(C)** Lyciumamide C in complex with the DAT protein. Left panels indicate the total number of hydrogen bond during the course of 100 ns simulation and right panel indicate the nature of hydrogen bond as hydrogen bond (H-bond) donors (green) and acceptors (blue).

The analysis of hydrogen bond dynamics over the 100 ns simulation period revealed important information about the stability and interactions of the three compounds with the DAT protein (Figure 8). Throughout the MD simulation, the total number of hydrogen bonds fluctuated in all three ligands, reflecting the dynamic nature of the interactions. Notably, the highest number of hydrogen bonds was observed with Lyciumamide C before 30 ns of simulation. This time indicates a stable interaction phase between Lyciumamide C and DAT protein. After this peak, the number of hydrogen bonds was observed to decrease for Lyciumamide C and Chenoalbicin, suggesting some loss of interaction strength as the simulation progressed. Despite these fluctuations, key hydrogen bonds remained consistently formed, contributing to the overall stability of the ligand-protein complex. This behavior highlights the importance of specific hydrogen bonds in maintaining the structural integrity of the complex during the simulation.

The dynamics of hydrogen bond donors and acceptors also provided valuable perspectives about the interactions between the top three compounds and the DAT protein. Throughout the 100 ns simulation, all three ligands consistently acted as hydrogen bond donors, forming stable interactions with several acceptor residues in the DAT protein (Figures 8A–C). The analysis indicated that certain residues in the DAT acted as persistent hydrogen bond acceptors, facilitating strong and reliable interactions with the ligand. It was observed that Chenoalbicin and Dipiperamide G showed dominance of hydrogen bond donors while Lyciumamide C showed a mix of both types of bonding but the number of hydrogen bond acceptors remained exceeded. The overall results demonstrate that both hydrogen bond donors and acceptors play crucial roles in the stability and functionality of the ligand-protein complex, emphasizing their significance in the binding affinity of the ligands studied.

### Cross-correlation mapping

**FIGURE 9:**
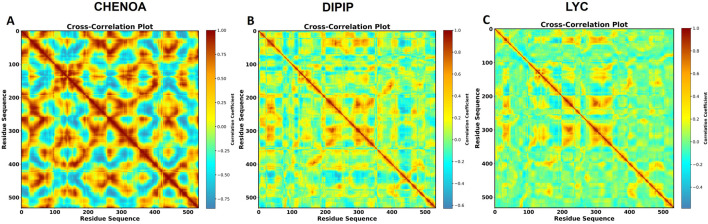
Cross-correlation plots indicating residue motions in the DAT protein complexes with **(A)** Chenoalbicin, **(B)** Dipiperamide G, and **(C)** Lyciumamide C. The residue sequence is shown by the x- and y-axes, and the correlation coefficients are shown on the right by the color scale, which goes from −1 (anti-correlated, blue) to +1 (positively correlated, red). The degree of correlated and anti-correlated motions between residues throughout the 100 ns MD simulation is displayed.

Cross-correlation maps were calculated for the three ligand-protein systems that were chosen to examine the effect of ligand binding on the internal dynamics of the protein (Figure 9). Intra-residue motions, or the movement of individual residues concerning one another, are represented by the diagonal elements of these maps. The off-diagonal areas, on the other hand, display the relative movements between various residues by reflecting inter-residue motions.

The level of association between residue pairs is indicated by the color coding. Blue areas show strong anti-correlations, which indicate that residues move in opposite directions, while red regions show highly positive correlations, which indicate coordinated motion between residues. By altering the correlated and anti-correlated motions of particular areas, ligand binding affects the internal dynamics of the protein, The cross-correlation map of the Chenoalbicin-DAT complex shows extensive positive correlations (red regions) along the diagonal, indicating strong intra-residue coordinated motions and overall rigidity (Figure 9A). This rigidity likely limits the adaptability of the protein to ligand binding, which aligns with the results of the binding free energy analyses. This pattern may restrict the DAT’s ability to adapt to dynamic environmental changes and can impact its functional performance. The Dipiperamide G and Lyciumamide C-DAT complexes exhibited similar patterns with fewer red and blue regions overall (Figures 9B, C). This lack of strong coordinated motions suggests enhanced flexibility and dynamic adaptability of the protein upon ligand binding. Such flexibility may facilitate efficient conformational changes necessary for functional activity. These observed patterns imply that Dipiperamide G and Lyciumamide C binding enhances the protein’s ability to adapt to external stimuli. These results are also supported by and aligned with the binding energy results of these two compounds, supporting the functional conformation of DAT complexes in terms of dynamic adaptability.

### Principal component analysis and free energy landscape

**FIGURE 10:**
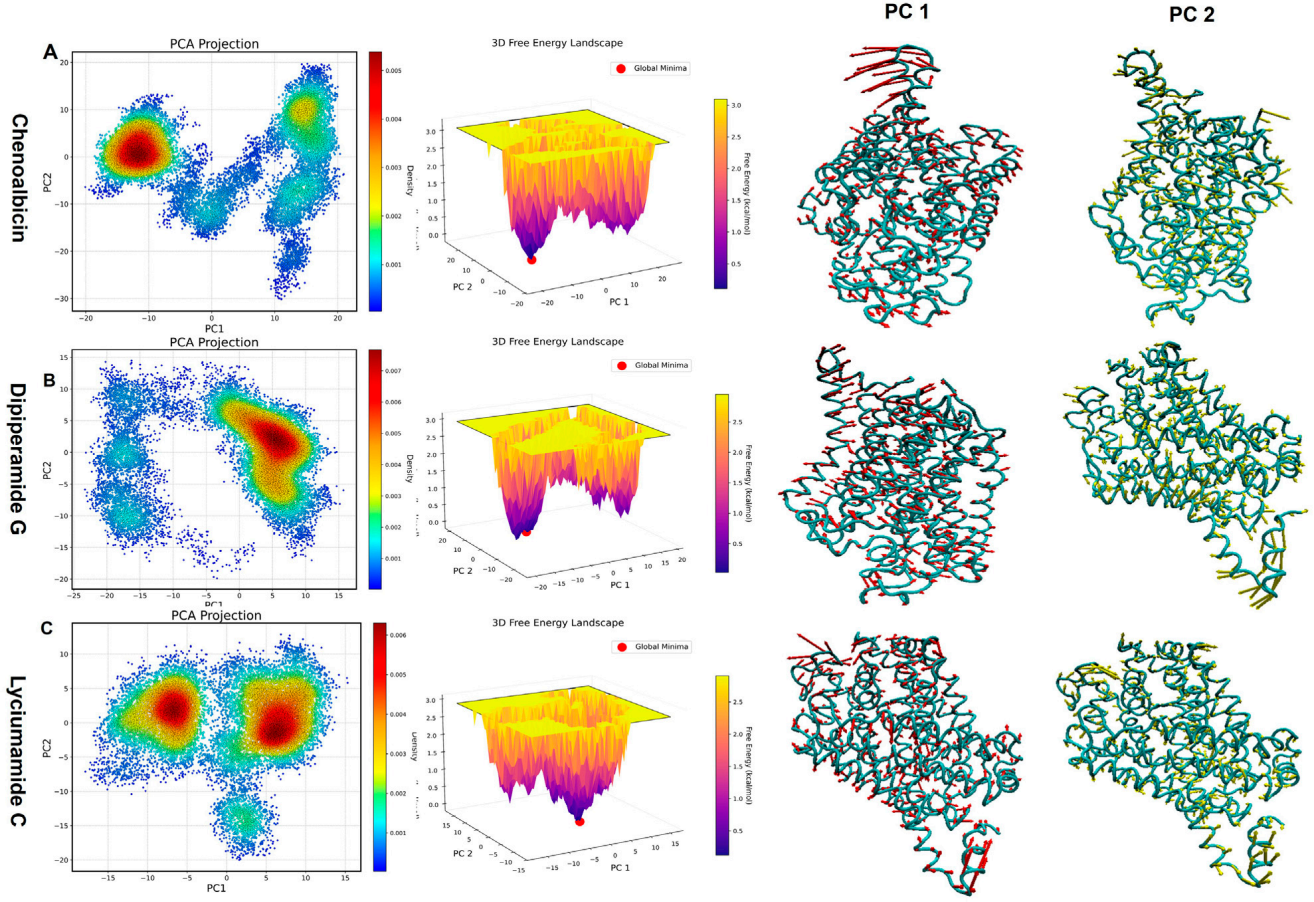
Principal Component Analysis (PCA) and Free Energy Landscape (FEL)representations for the dynamics of the DAT-ligand complexes with **(A)** Chenoalbicin, **(B)** Dipiperamide G, and **(C)** Lyciumamide C. Left panels: The movement trajectories along the first two principal components (PC1 and PC2) are displayed by PCA projections. Middle panels: Purple denotes high energy states and yellow denotes low energy (stable) states. FELs show energy fluctuations across various structural states. Panels on the right: For every compound, conformational changes during the course of the simulation, both PC1 and PC2. Greater mobility is shown by thicker ribbons, whereas greater rigidity is indicated by thinner ribbons.

Key motion patterns in the DAT-ligand systems were found using Principal Component Analysis (PCA) (Figure 10). Larger conformational shifts are correlated with higher eigenvalues, which represent the variance in movement. Different clustering patterns are revealed by the PCA projections, suggesting unique dynamics for every complex.

The PCA projection of Chenoalbicin-DAT showed a tightly packed cluster positioned at the negative PC1 and positive PC2 axes (Figure 10A). This positioning reflects less conformational flexibility with minimal variability. The free energy landscape (FEL) reveals deep, well-defined low-energy basins, indicating high stability. Chenoalbicin binding may help maintain its functional conformation but could limit adaptability in dynamic environments, as noted in the results of the binding free energy and cross, correlation analyses.

The PCA plot for Dipiperamide G-DAT showed clusters shifted toward the positive PC1 and positive PC2 regions (Figure 10B). This indicates favorable conformational shifts in both principal components. This dense cluster reflects a high amount of stability and flexibility. The FEL highlights broader low-energy basins. This suggests that Dipiperamide G binding allows adaptability while maintaining structural stability, making it suitable for dynamic conditions.

Lyciumamide C-DAT displayed two clusters along the positive PC2, indicating a preference for these conformations (Figure 10C). However, the clustering was observed for both the positive and negative values of PC1, suggesting that the first principal component motion was not always present. The clusters are more dispersed, showing greater variability compared to the other complexes. The FEL features multiple interconnected low-energy basins, indicating stable transitions between conformations, supported by the PCA results. Overall, the results of PCA and FEL indicate that Lyciumamide C promotes adaptability and dynamic behavior among the three systems.

### Structural analysis of the top metabolites

**FIGURE 11:**
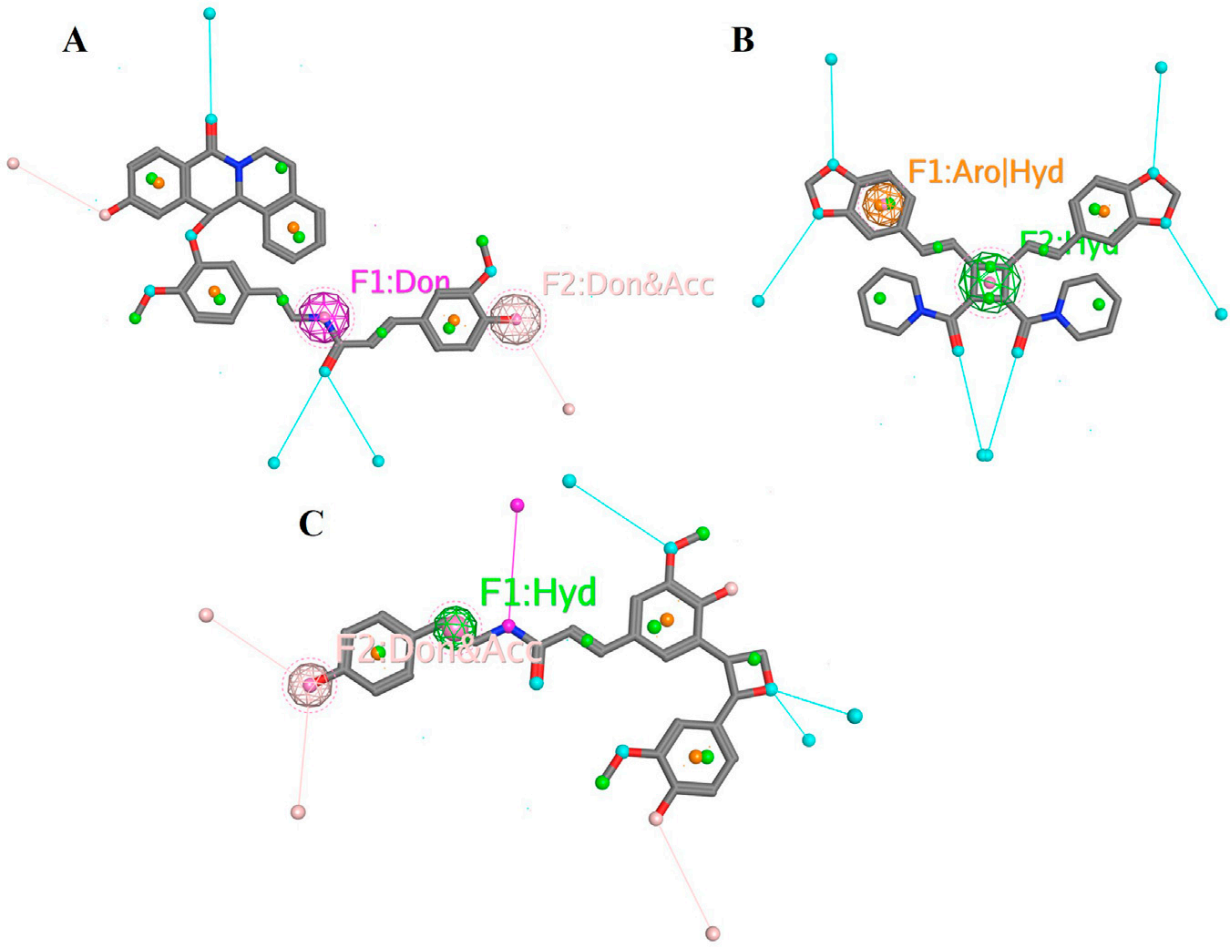
Pharmacophoric features of selected metabolites. **(A)** Pharmacophoric features of Chenoalbicin whereas F1 represents a hydrogen bond donor site and F2 represents a site that can act both as a hydrogen bond donor and acceptor. **(B)** Pharmacophoric features of Dipiperamide G whereas F1 represents an aromatic or hydrophobic interaction site and F2 represents a hydrophobic interaction site. **(C)** Pharmacophoric features of Lyciumamide C whereas F1 represents a hydrophobic interaction site and F2 indicates a site that can act both as a hydrogen bond donor and acceptor.

The pharmacophoric features of these metabolites are highlighted based on their interaction patterns aforementioned in the study. The molecular structure of Chenoalbicin shows distinct donor and donor-acceptor pharmacophoric features (Figure 11A). The presence of hydrogen bond donor (F1) and dual donor-acceptor (F2) sites suggests potential interactions with multiple biological targets through hydrogen bonding. Such features are particularly relevant for DAT inhibition, as they enable stable interactions with critical residues within the active site of the DAT, such as Asp475 and Arg476, as observed in docking and interaction analyses. Moreover, Dipiperamide G features both aromatic/hydrophobic (F1) and purely hydrophobic (F2) interaction sites (Figure 11B). The presence of aromatic/hydrophobic sites suggests strong interactions with hydrophobic regions of target proteins (as discussed in the active site portion of the receptor), which can contribute to the stabilization of ligand-receptor complexes. Hydrophobic interactions are crucial for the bioavailability and membrane permeability of the molecule. Structure Analysis of Lyciumamide C exhibits a combination of hydrophobic (F1) and dual donor-acceptor (F2) pharmacophoric features (Figure 11C). These features correlate with its strong binding affinity (−10.03 kcal/mol) and eventual stabilization after 15 ns of simulation (RMSD: 2.3 Å ± 1 Å). The hydrophobic feature (F1) facilitates interactions with hydrophobic pockets such as Tyr123 and Val120. The dual donor-acceptor site (F2) allows for versatile hydrogen bonding interactions, such as Asp46 (H-donor) and Phe319 (H-pi), enhancing binding strength and specificity for DAT inhibition.

The analyzed compounds exhibit a diverse range of pharmacophoric features that are critical for their interaction with DAT. These interactions are essential for the biological activity of the compounds, suggesting their potential as therapeutic agents for the treatment of schizophrenia. Understanding the pharmacophoric features of top metabolites can guide the design and optimization of new molecules with improved efficacy and selectivity for DAT.

### Evaluation of physicochemical properties

The SwissADME server was used to study the physicochemical properties of ten phytochemicals. All phytochemicals exhibited optimal physicochemical properties. The LogP values of ten metabolites ranged from 3.07 to 5.5 (Supplementary Table S8), demonstrating better solubility and membrane permeability than the standard compound (Lipinski et al., 1997). This suggests that the metabolites will be soluble enough for transport in bodily fluids and lipophilic enough to cross cell membranes. The number of hydrogen bond donors (HBD), acceptors (HBA), and rotatable bonds (Rot. B) in these metabolites were comparable to those in the standard compound, indicating a balance between structural flexibility and rigidity. Furthermore, their potential for effective hydrogen bonding is supported by interaction analysis results. The molecular weights (MW) of these metabolites were lower than those of the standard compound, implying superior cell membrane permeability, which is crucial for oral bioavailability. Additionally, the optimal topological polar surface area (TPSA) values suggest that these metabolites possess the favorable polar surface required for interactions with aqueous environments in the body.

**FIGURE 12:**
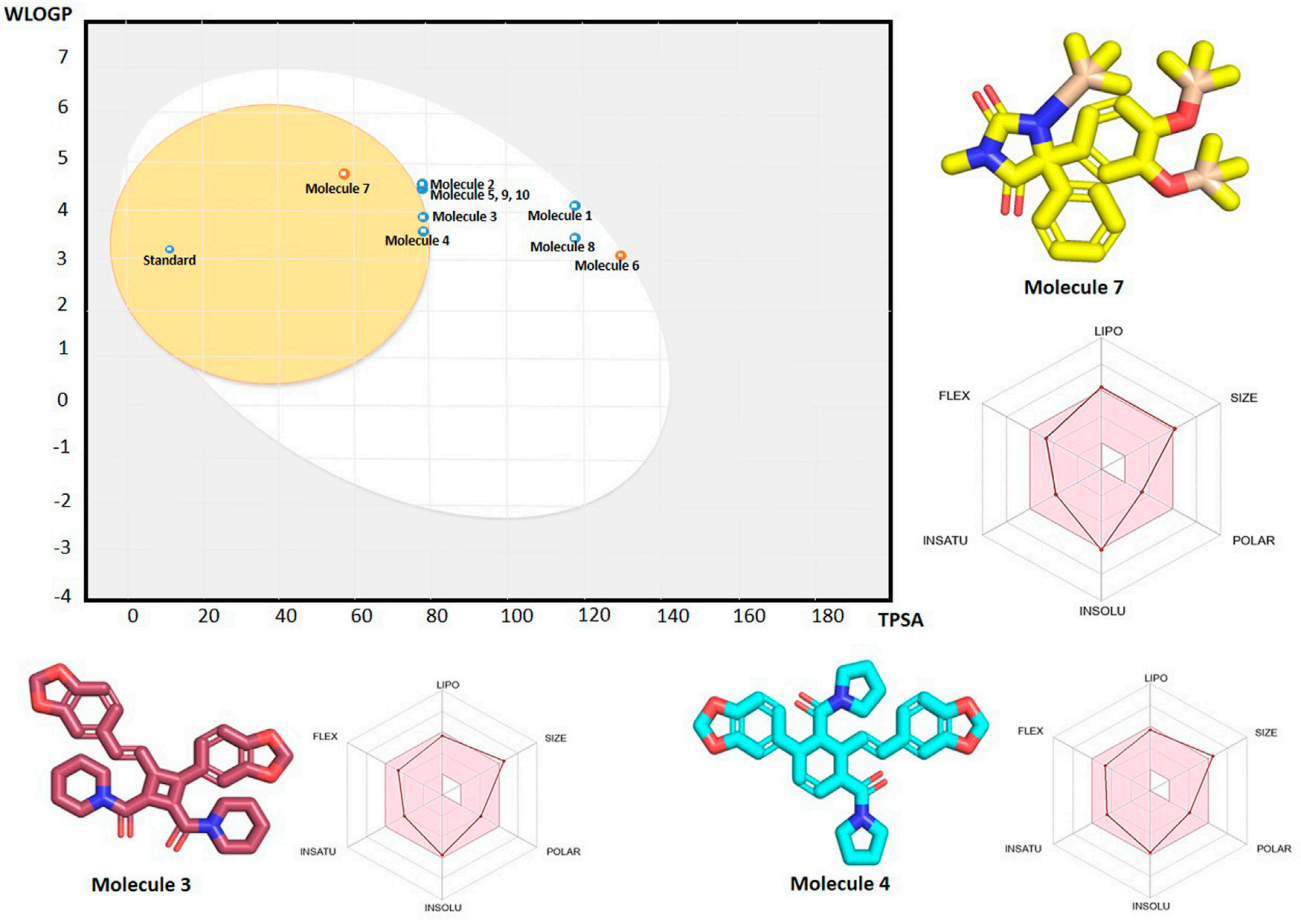
The plot on the left represents the WLOGP versus TPSA values, illustrating the boiled egg diagram of top 10 metabolites from SwissADME where the Molecule 7, 3, 4 and standard fall within the BBB permeability range (Egg Yolk), while others are outside this range (Egg White). The 3D structure of three blood brain barrier penetrating metabolites namely Molecule 7 (yellow), molecule 3 (pink) and molecule 4 (cyan) are displayed along with their radar plots. The radar plot illustrates the molecular properties, including lipophilicity (LIPO), size, polarity (POLAR), solubility (INSOLU), and flexibility (FLEX).

Among the ten metabolites, Metabolite 7 (3,12-di-O-acetyl-8-O-tigloylingol) and Metabolite 8 (Lyciumamide C) exhibited the best physicochemical properties (Supplementary Table S8). This can be indicated by the radar plot of the SwissADME for metabolite 7 (Figure 12) The colored zone represents the optimal physicochemical space for oral bioavailability. The parameters include lipophilicity, where the XLOGP3 value ranges from −0.7 to +5.0. The molecular size should fall between 150 g/mol and 500 g/mol. Polarity is measured with a Topological Polar Surface Area (TPSA) ranging from 20 Å^2^ to 130 Å^2^. Insolubility is defined by a Log S (ESOL) value between −6 and 0. The degree of saturation is indicated by the Fraction Csp3, which should be between 0.25 and 1. Finally, flexibility is measured by the number of rotatable bonds, which should be between 0 and 9.

### Blood-brain barrier permeability

BBB permeability is a crucial property for drugs targeting schizophrenia, as it determines the ability of compounds to cross the BBB. The BBB permeability profile of the top 10 metabolites, along with the standard lumataperone, was assessed using the BOILED-Egg diagram from SwissADME (Figure 12). The results indicated that only metabolites 7, 3, and 4, along with the standard, fell within the BBB range. The oral bioavailability profile of three BBB permeable metabolites is shown in Figure 12. Notably, metabolite 7 is not a P-glycoprotein (P-gp) substrate, which is advantageous as it suggests lower efflux and better brain penetration. Additionally, metabolites 2, 5, 9, and 10 were observed to be near the BBB range. It suggests that some modifications to their structure could enhance their permeability.

When integrated with molecular docking and pharmacokinetics results, the BOILED-Egg predictions reinforce the potential of metabolites 7, 3, and 4 as strong candidates for further experimental validation. Their ability to penetrate the BBB, combined with favorable binding affinities and stability profiles observed in molecular dynamics, highlights their therapeutic promise. Experimental studies, such as *in vitro* BBB models or *in vivo* CNS penetration assays, will be necessary to confirm these predictions and further validate their potential for clinical development.

### Analysis of biological activity

A bioactivity score quantitatively assesses a metabolite’s biological activity and predicts its interaction with biological targets like receptors, enzymes, and ion channels. These targets are crucial for new drug development due to their role in physiological processes (Khalid et al., 2024b). Metabolites are categorized based on this particular score: scores above 0 indicate high biological activity; scores between −0.50 and 0.0 suggest moderate activity; and scores below −0.50 classify metabolites as inactive. This method provides valuable information for research and drug development by prioritizing metabolites based on their potential effectiveness (Khalid et al., 2024b).

The molinspiration online web server was used to analyze the bioactivity score of the ten top metabolites along with the standard compound. Metabolite 7, namely, 3,12-di-O-acetyl-8-O-tigloylingol, 2,4-Imidazolidinedione,5-[3,4-bis[(trimethylsily)oxy] phenyl], -3-methyl-5-phenyl-1-(trimethylsilyl) and metabolite 8, namely, lyciumamide C, showed a good bioactivity score among ten metabolites, while all remaining metabolites exhibited an intermediate bioactivity score (Supplementary Table S9). Their bioactivity values suggest their potent role in the physiological process when administered as drugs, thereby making them strong candidates for drug development.

### Analysis of drug-like properties

The drug-likeness properties of selected phytochemicals were predicted by different rules, including Lipinski’s rule (Lipinski et al., 1997), Veber’s rule (Veber et al., 2002), Egan’s rule (Egan et al., 2000) and Muegge’s rule (Muegge et al., 2001), using the SwissADME online server (Table 1). Among ten metabolites, metabolite 8 (lyciumamide C) followed all the rules with no violation and showed a good bioavailability score (0.55) similar to the standard compound (Lumateperone), and the existing DAT inhibitors, amphetamine, and methylphenidate (Table 1), suggesting its high potential to be used as a novel antiseptic agent. This consistency of Lyciumamide C across multiple drug-like property rules highlights its strong potential for clinical development. Other metabolites, including Metabolite 1 (Chenoalbicin) and Metabolite 2 (Dipiperamides G), followed Lipinski’s rule, with no or one violation having an optimal bioavailability score of 0.55. These results predicted that all the selected phytochemicals strongly adhered to key drug-like properties.

**TABLE 1:** Drug-likeness Analysis of the potential hits.

Metabolite no.	Lipinski Rule	Egan rule	Veber rule	Muegge rule	Bioavailability score	QED
Lumateperone	Yes (0 violation)	Yes	Yes	Yes	0.55	0.72
Amphetamine	Yes (0 violation)	Yes	Yes	No	0.55	0.65
Methylphenidate	Yes (0 violation)	Yes	Yes	Yes	0.55	0.81
1	Yes (1 violation)	Yes	Yes	No	0.55	0.22
2	Yes (1 violation)	Yes	Yes	No	0.55	0.46
3	Yes (1 violation)	Yes	Yes	Yes	0.55	0.53
4	Yes (1 violation)	Yes	Yes	Yes	0.55	0.51
5	Yes (1 violation)	Yes	Yes	Yes	0.55	0.43
6	Yes (1 violation)	Yes	Yes	Yes	0.55	0.19
7	Yes (1 violation)	Yes	Yes	No	0.55	0.35
8	Yes (0 violation)	Yes	Yes	Yes	0.55	0.33
9	Yes (1 violation)	Yes	Yes	No	0.55	0.44
10	Yes (1 violation)	Yes	Yes	Yes	0.55	0.43

1: Chenoalbicin, 2: Dipiperamide G, 3: Nigramide R, 4: Chabamide G, 5: Dipiperamide F, 6: 3,12-di-O-acetyl-8-O-tigloylingol, 7: 2,4-Imidazolidinedione,5-[3,4-bis[(trimethylsily)oxy] phenyl]-3-methyl-5-phenyl-1-(trimethylsilyl), 8: Lyciumamide C 9: Chabamide, 10: Dipiperamide E, and QED: Quantitative Estimate of Drug-likeness.

A quantitative estimate of drug-likeness (QED) analysis was performed using the ADMETlab 3.0 server to assess the drug-likeness of the selected metabolites. According to the classification criteria, QED values greater than 0.67 are considered “attractive,” values between 0.49 and 0.67 are classified as “unattractive,” and values below 0.34 are categorized as “too complex.” The standard compound achieved a QED score of 0.72, categorizing it as “attractive.” Among the screened metabolites, Metabolites 4 (0.53) and 5 (0.51) demonstrated QED values within the “unattractive” range, indicating potential for further refinement. Metabolites 3 (0.46), 6 (0.43), and 10 (0.44) scored slightly lower but still show promise as lead compounds for optimization. However, Metabolites 2 (0.22), 7 (0.19), 8 (0.35), and 9 (0.33) were classified as “too complex”. These findings underscore the potential of the screened metabolites for drug discovery.

### Analysis of ADMET/pharmacokinetics properties

The online web server pkCSM was used to evaluate the top ten metabolites that generated the complete profile of the pharmacokinetic properties of selected phytochemicals (Table 2). This evaluation included intestinal absorption, skin permeability, water solubility, BBB permeability, and different toxicity properties. Most metabolites acted as P-glycoprotein II inhibitors, except Metabolite 7 (3,12-di-O-acetyl-8-O-tigloylingol), which is advantageous for brain-targeted therapies as it reduces the likelihood of efflux from the CNS. In the CYP450 metabolism, all metabolites were inhibitors of CYP3A4, a critical enzyme for drug metabolism. Additionally, Metabolites 1, 3, 4, 8, and 9 were inhibitors of CYP2C19. All selected phytochemicals, including the standard compound, were not inhibitors of CYP1A2. All metabolites exhibited no toxicity to AMES and no skin sensitization. Metabolites 4, 6, and the standard showed no hepatotoxicity. These findings highlight the favorable safety and pharmacokinetic profiles of the studied metabolites. When integrating ADMET properties with earlier findings, most of these metabolites exhibited high intestinal absorption, low toxicity, favorable BBB permeability, and strong adherence to docking and bioactivity predictions. Such consistency across computational assessments highlights their potential for successful translation into drugs.

**TABLE 2:** ADMET profiling of the potential hits.

Absorption Parameters					
Metabolite No.	Water solubility	CaCo2 permeability	Intestinal Absorption (%)	Skin Permeability	P glycoprotein II inhibitor
*	−4.71	1.59	96	−2.95	Yes
1	−3.41	1.432	100	−2.74	Yes
2	−5.02	1.15	95	−2.75	Yes
3	−4.52	1.06	97	−2.78	Yes
4	−4.51	1.08	99	−2.81	Yes
5	−4.88	1.11	97	−2.76	Yes
6	−4.64	1.02	81	−2.79	Yes
7	−5.99	1.19	90	−3.29	No
8	−4.63	0.68	91	−2.73	Yes
9	−4.32	1.17	97	−2.77	Yes
10	−4.88	1.10	97	−2.75	Yes

Distribution Parameters				
Metabolite No.	VDss (Human)	CNS Permeability	BBB Permeability (LogBB)	Fraction Unbound (Human)
*	1.26	−1.15	0.50	0.02
1	−0.83	−2.96	−0.94	0.204
2	−0.34	−2.75	−0.82	0.015
3	−0.18	−2.86	−0.51	0
4	−0.09	−2.92	−0.50	0
5	−0.32	−2.83	−0.81	0.01
6	0.58	−2.79	−0.95	0.19
7	0.16	−2.08	−0.19	0
8	−0.42	−3.22	−0.93	0.04
9	0.04	−2.79	−0.04	0
10	−0.31	−2.82	−0.81	0.01

Metabolic Parameters					
Metabolite No.	CYP1A2 Inhibitor	CYP3A4 Inhibitor	CYP2C9 Inhibitor	CYP2C19 Inhibitor	CYP2D6 Inhibitor
*	No	Yes	No	No	Yes
1	No	Yes	Yes	Yes	No
2	No	Yes	Yes	No	No
3	No	Yes	Yes	Yes	No
4	No	Yes	Yes	Yes	No
5	No	Yes	Yes	No	No
6	No	Yes	No	No	No
7	No	Yes	No	No	No
8	No	Yes	Yes	Yes	No
9	No	Yes	Yes	Yes	No
10	No	Yes	Yes	No	No

Excretion and Toxicity Parameters						
Metabolite No.	Total Clearance	Renal OTC2 Substrate	AMES Toxicity	Skin Sensitization	hERG I Inhibitor	Hepatotoxicity
*	1.11	No	No	No	No	No
1	0.397	No	No	No	No	Yes
2	0.076	No	No	No	No	Yes
3	−0.04	No	No	No	No	Yes
4	0.27	No	No	No	No	No
5	−0.08	No	No	No	No	Yes
6	0.50	No	No	No	No	No
7	0.41	No	No	No	No	Yes
8	0.37	No	No	No	No	Yes
9	−0.05	No	No	No	No	Yes
10	−0.07	No	No	No	No	Yes

1: Chenoalbicin, 2: Dipiperamide G, 3: Nigramide R, 4: Chabamide G, 5: Dipiperamide F, 6: 3,12-di-O-acetyl-8-O-tigloylingol, 7: 2,4-Imidazolidinedione,5-[3,4-bis[(trimethylsily) oxy]phenyl]-3-methyl-5-phenyl-1-(trimethylsilyl), 8: Lyciumamide C 9: Chabamide, 10: Dipiperamide E, and Asterisk (*): Standard.

### Concentration time profile of selected metabolites

The PBPK simulations generated concentration-time profiles for the unbound fraction of three shortlisted metabolites, namely, chenoalbicin, dipiperamide G (cinnamic acid amide alkaloid), and lyciumamide C (phenolic amide), in the brain interstitial fluid over 24 h. These profiles represented their potential efficacy and duration of action in the target tissue.

**FIGURE 13:**
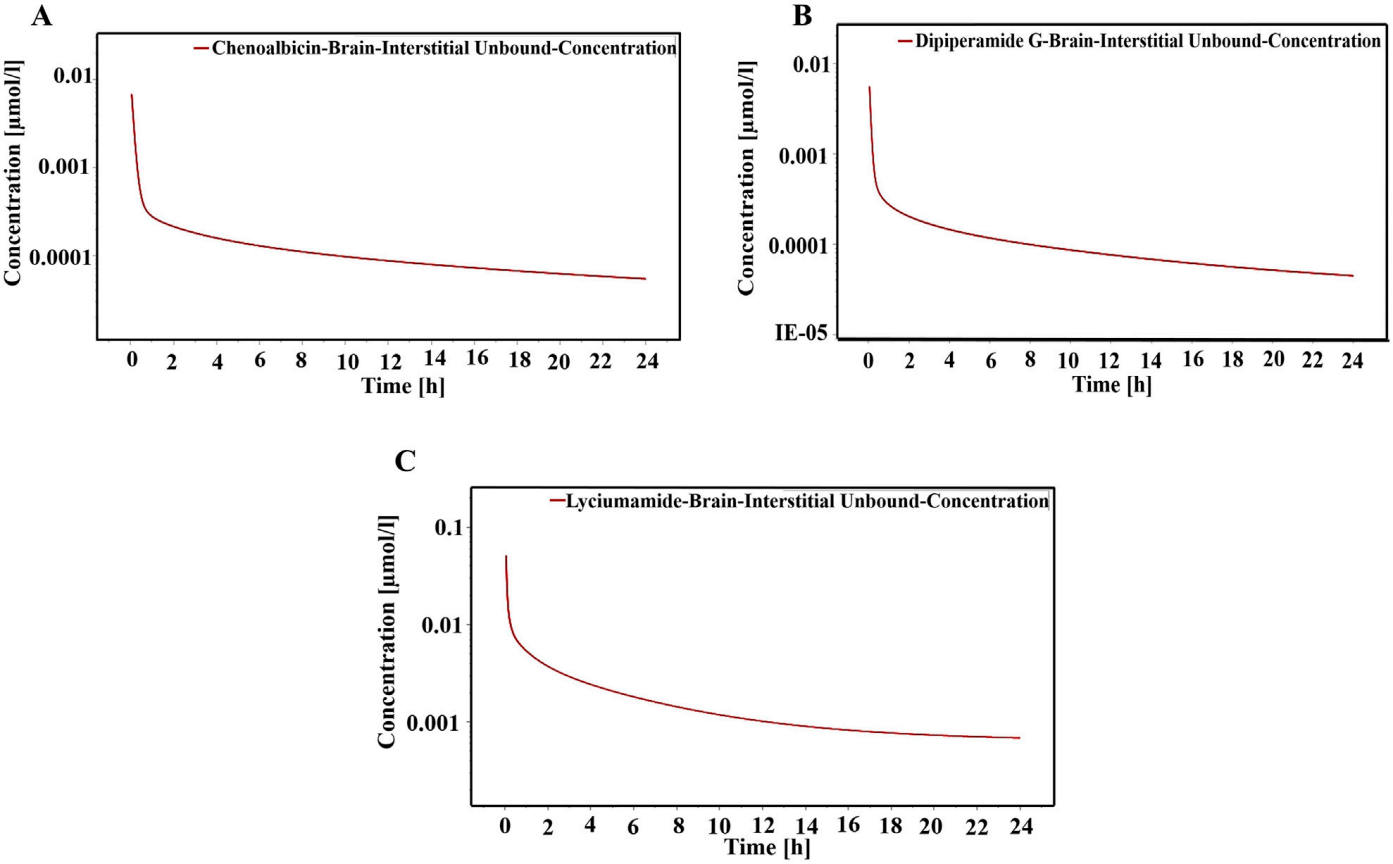
Concentration time profile of **(A)** Chenoalbicin, **(B)** Dipiperamide G and **(C)** Lyciumamide C. The red solid line represents various concentrations of these metabolties in the brain interstitial fluid over a 24-h period.

The brain interstitial unbound concentration of Chenoalbicin peaked rapidly at approximately 0.01 μmol/L within the first hour post-administration (Figure 13A). The concentration then declined following a biphasic pattern, with a rapid initial decline followed by a slower elimination phase. At 24 h, the concentration remained above 0.00005 μmol/L, suggesting potential for once-daily dosing. Dipiperamide G showed a similar profile to Chenoalbicin, with a rapid peak concentration of about 0.008 μmol/L (Figure 13B). However, its elimination appeared to be slightly faster, with concentrations dropping below 0.00005 μmol/L by 24 h. This might necessitate more frequent dosing compared to Chenoalbicin. Lyciumamide C demonstrated the highest peak concentration among the three metabolites, reaching above 0.05 μmol/L (Figure 13C). Its elimination profile was more gradual, maintaining higher concentrations throughout the 24 h. At 24 h, the concentration remained above 0.0005 μmol/L, indicating a potentially longer duration of action.

Comparatively, the peak concentrations and sustained levels can be indicated as Lyciumamide C > Chenoalbicin > Dipiperamide G. The higher and more sustained brain concentrations of Lyciumamide C suggest it may have the most promising pharmacokinetic profile for targeting the DAT in schizophrenia treatment. Its lower molecular weight and moderate lipophilicity likely contribute to its superior brain penetration and retention. Chenoalbicin shows a balanced profile with good initial concentrations and sustained levels, potentially suitable for once-daily dosing. Dipiperamide G, while reaching therapeutic concentrations, may require more frequent dosing due to its faster elimination. Therefore, all three metabolites demonstrate the ability to cross the BBB and maintain potentially therapeutic concentrations in the brain interstitial fluid. These findings align with their strong binding affinities and pharmacokinetic properties, reinforcing their potential as therapeutic agents for schizophrenia treatment. It must be noted here that the BBB permeability results from the SwissADME webserver contradict these results where SwissADME did not predict the BBB permeable nature of these three metabolites. SwissADME predictions rely on static cheminformatics methods based on molecular characteristics, whereas the PBPK simulations predict the behavior of substances in a physiological system and dynamically model drug distribution in tissues and organs. PBPK results offer a more thorough and accurate evaluation (Daina et al., 2017; Jones et al., 2011; Zhuang & Lu, 2016).

The difference in these results might be due to the variable algorithms that form the basis of these two software. Experimental studies are demanded to the confirm permeability of these metabolites. Further, their efficacy would depend on their binding affinity and inhibitory potency against the DAT, which would also require further *in vitro* and *in vivo* studies.

### Summarized findings, limitations, and future directions

Chenoalbicin showed the strongest docking affinity (−10.45 kcal/mol), followed by Dipiperamide G (−10.31 kcal/mol) and Lyciumamide C (−10.03 kcal/mol), all outperforming the standard drug lumateperone (−7.57 kcal/mol). Chenoalbicin formed two hydrogen bonds with Asp475 and Arg476, while Dipiperamide G interacted with Asp475 and Phe325. Lyciumamide C bonded with Phe319 and Ser320, showing strong hydrophobic and hydrogen bonding interactions. All three showed stable RMSD values over 100 ns of simulation. Chenoalbicin exhibited donor-acceptor features supporting strong hydrogen bonding, Dipiperamide G featured hydrophobic and aromatic interaction sites, and Lyciumamide C combined dual donor-acceptor and hydrophobic features. All three metabolites demonstrated optimal physicochemical properties, with LogP values supporting solubility and membrane permeability, and TPSA values indicating good oral bioavailability. All three showed high intestinal absorption and low toxicity. Lyciumamide C demonstrated the highest brain concentration and sustained action, followed by Chenoalbicin, which showed balanced pharmacokinetics, while Dipiperamide G exhibited faster elimination, suggesting frequent dosing requirements. All three showed BBB penetration in PK simulation, while only Dipiperamide G was near the BBB range in the BOILED-Egg diagram. Moreover, all three indicated stable results in MD analysis with Lyciumamide C being more potent. These findings collectively highlight Chenoalbicin, Dipiperamide G, and Lyciumamide C as promising lead candidates for the development of brain-targeted drugs to treat schizophrenia.

Potential synergy can be observed between the identified hits due to their complementary pharmacophoric features and binding profiles. For instance, while Lyciumamide C exhibits strong hydrophobic and donor-acceptor interactions that stabilize its binding, Chenoalbicin’s hydrogen bond donors may complement these effects by targeting additional residues in DAT. From a pharmacokinetic perspective, combining compounds with varying ADME profiles might enable sustained therapeutic levels and reduce dosing frequency. However, potential limitations, such as the risk of drug-drug interactions and challenges in achieving optimal dosing ratios, should be carefully evaluated.

The study has limitations, including the reliance on computational predictions that require experimental validation for the identified phytochemicals. The analysis of drug-likeness and ADMET profiles may not capture all potential interactions in biological systems. The docking analyses were performed using the DAT structure (PDB ID: 4M48), derived from *Drosophila melanogaster* and *Mus musculus*, and expressed in *Homo sapiens*. Although this structure is highly relevant, future studies incorporating human DAT structures would provide additional validation of our findings. Binding assays, such as radioligand displacement studies, should be performed to evaluate the affinity of the identified metabolites for DAT. Additionally, PET imaging with radiolabeled derivatives, cell-based assays, and animal models could validate *in vivo* interactions (Volkow et al., 1995).

## Conclusion

This research aimed to identify compounds for improving the cognitive disabilities in schizophrenia. While antipsychotic drugs are widely used, their efficacy declines over time due to resistance and side effects. Our studied drug target, DAT, helps treat resistance to antipsychotic treatment. We screened 990 secondary metabolites from medicinal plants to identify potential anti-schizophrenic agents. The top ten metabolites showed strong binding affinities promising drug-like properties and favorable toxicity profiles compared to the standard, Lumateperone. Moreover, the potential hits were stable in the active site of DAT with very low deviations. The MD analyses of Chenoalbicin, Dipiperamide G, and Lyciumamide C revealed that while all three ligands displayed favorable dynamics, Lyciumamide C was the most stable and energetically favorable for binding in dynamic environments. Pharmacophoric analysis revealed critical features for their biological activity. The pharmacokinetic simulation showed their sustained binding with DAT over 24 h. Clinical trials are essential to validate these findings and determine optimal dosing strategies for all three metabolites (Chenoalbicin, Dipiperamide G, and Lyciumamide C). Amides and alkaloids from members of the family of the Piper retrofractum show potential as anti-schizophrenic agents. These findings highlight the potential of the identified compounds to serve as novel DAT-targeted therapeutics for managing cognitive symptoms in schizophrenia. Future experimental validation and clinical studies will be crucial to confirm their efficacy, safety, and translational applicability in therapeutic settings.

## Data Availability

The original contributions presented in the study are included in the article/Supplementary Material, further inquiries can be directed to the corresponding authors.

## Supplementary material

SUPPLEMENTARY FIGURE S1 Docking interaction analysis of metabolite 2 (Dipiperamide G); 2D interaction map **(A)** with red indicating hydrogen-bonded residues, and 3D conformation **(B, C)** in the binding pocket of dopamine transporter protein.

SUPPLEMENTARY FIGURE S2 Docking interaction analysis of metabolite 8 (Lyciumamide C); 2D interaction map **(A)** with red indicating hydrogen-bonded residues, and 3D conformation **(B, C)** in the binding pocket of dopamine transporter protein.

SUPPLEMENTARY FIGURE S3 Docking interaction analysis of metabolite 3 (Nigramide R); 2D interaction map **(A)** with red indicating hydrogen-bonded residues, and 3D conformation **(B, C)** in the binding pocket of dopamine transporter protein.

SUPPLEMENTARY FIGURE S4 Docking interaction analysis of metabolite 4 (Chabamide G); 2D interaction map **(A)** with red indicating hydrogen-bonded residues, and 3D conformation **(B, C)** in the binding pocket of dopamine transporter protein.

SUPPLEMENTARY FIGURE S5 Docking interaction analysis of metabolite 5 (Dipiperamide F); 2D interaction map **(A)** with red indicating hydrogen-bonded residues, and 3D conformation **(B, C)** in the binding pocket of dopamine transporter protein.

SUPPLEMENTARY FIGURE S6 Docking interaction analysis of metabolite 6 (3,12-di-O-acetyl-8-O-tigloylingol); 2D interaction map **(A)** with red indicating hydrogen-bonded residues, and 3D conformation **(B, C)** in the binding pocket of dopamine transporter protein.

SUPPLEMENTARY FIGURE S7 Docking interaction analysis of metabolite 7 (2,4-Imidazolidinedione,5-[3,4-bis[(trimethylsily)oxy]phenyl]-3-methyl-5-phenyl-1-(trimethylsilyl); 2D interaction map **(A)** with red indicating hydrogen-bonded residues, and 3D conformation **(B, C)** in the binding pocket of dopamine transporter protein.

SUPPLEMENTARY FIGURE S8 Docking interaction analysis of metabolite 9 (Chabamide); 2D interaction map **(A)** with red indicating hydrogen-bonded residues, and 3D conformation **(B, C)** in the binding pocket of dopamine transporter protein.

SUPPLEMENTARY FIGURE S9 Docking interaction analysis of metabolite 10 (Dipiperamide E); 2D interaction map **(A)** with red indicating hydrogen-bonded residues, and 3D conformation **(B, C)** in the binding pocket of dopamine transporter protein.
