# Supplementary material for: Identification of novel amides and alkaloids as putative inhibitors of dopamine transporter for schizophrenia using computer-aided virtual screening
Source: Front Pharmacol. 2025 Apr 8;16:1509263. doi: 10.3389/fphar.2025.1509263 (PMC12039762; doi:10.3389/fphar.2025.1509263)
Supplement: Supplementary file 2 [file Table1.docx]

**Table S1.** Library of the secondary metabolites of *Piper retrofractum.*

| **Sr.**  **No.** | **Compound** | **Structure** | **Docking value**  **(Kcal/mol)** | **References** |
| --- | --- | --- | --- | --- |
|  | Retrofractamide A |  | -7.61 | (Farediah AHMAD, 2020) |
|  | Retrofractamide B |  | -7.80 | (Farediah AHMAD, 2020) |
|  | Retrofractamide C |  | -7.59 | (Farediah AHMAD, 2020) |
|  | Retrofractamide D |  | -8.42 | (Farediah AHMAD, 2020) |
|  | Methyl piperate |  | -5.99 | (Farediah AHMAD, 2020) |
|  | Piperlonguminine |  | -6.53 | (Farediah AHMAD, 2020) |
|  | Dihydropiperlonguminine |  | -7.33 | (Farediah AHMAD, 2020) |
|  | Guineensine |  | -9.13 | (Farediah AHMAD, 2020) |
|  | Sylvatine |  | -8.19 | (Farediah AHMAD, 2020) |
|  | Pellitorine |  | -6.58 | (Farediah AHMAD, 2020) |
|  | Piplartine |  | -7.49 | (Farediah AHMAD, 2020) |
|  | Piperodione |  | -7.36 | (Farediah AHMAD, 2020) |
|  | (*2E,12E*)-pipertridecadienamide |  | -9.22 | (Farediah AHMAD, 2020) |
|  | N-isobutyl-(*2E,4E,10Z*)-hexadeca-2,4,10-trienamide |  | -6.19 | (Farediah AHMAD, 2020) |
|  | Isopiperine |  | -6.20 | (Farediah AHMAD, 2020) |
|  | Isochavicine |  | -6.62 | (Farediah AHMAD, 2020) |
|  | 5,6-Dihydro-1*H*-pyridin-2-one |  | -4.53 | (Farediah AHMAD, 2020) |
|  | *N*-isobutyl-(2E,4E)- hexadeca-2,4-dienamide |  | -8.05 | (Farediah AHMAD, 2020) |
|  | *N*-Cinnamoylpiperidine |  | -5.82 | (Farediah AHMAD, 2020) |
|  | Piperoleine B |  | -8.05 | (Farediah AHMAD, 2020) |
|  | Piperchabamide B |  | -8.05 | (Farediah AHMAD, 2020) |
|  | Piperchabamide C |  | -7.87 | (Farediah AHMAD, 2020) |
|  | Piperchabamide H |  | -10.30 | (Farediah AHMAD, 2020) |
|  | Piperundecaline |  | -7.06 | (Farediah AHMAD, 2020) |
|  | Dehydropipernonaline |  | -8.02 | (Farediah AHMAD, 2020) |
|  | Pipernonaline |  | -7.83 | (Farediah AHMAD, 2020) |
|  | *N*-isobutyl-*2E,4E,12Z*-octadecatrienamide |  | -7.99 | (Farediah AHMAD, 2020) |
|  | *N*-isobutyl-*2E,4E,14Z*-eicosatrienamide |  | -9.21 | (Farediah AHMAD, 2020) |
|  | 1-(octadeca-*2E,4E,12Z*-trienoyl)piperidine |  | -6.47 | (Farediah AHMAD, 2020) |
|  | 1-(eicosa-*2E,4E,14Z*-trienoyl)piperidine |  | -6.07 | (Farediah AHMAD, 2020) |
|  | 1-(octadeca-*2E,4E*-dienoyl)piperidine |  | -5.88 | (Farediah AHMAD, 2020) |
|  | 1-(eicosa-*2E,4E*-dienoyl)piperidine |  | -5.99 | (Farediah AHMAD, 2020) |
|  | 1-(eicosa-*2E,14Z*dienoyl)piperidine |  | -6.31 | (Farediah AHMAD, 2020) |
|  | (*E*)-*N*-cinnamoyl-2-methoxypiperidine |  | -6.83 | (Farediah AHMAD, 2020) |
|  | (*R*)-1-(2-oxopyrrolidin-3-yl)-5,6-dihydropyridin-2(1*H*)-one |  | -5.90 | (Farediah AHMAD, 2020) |
|  | (*E*)-*N*-(Tetrahydro-*2H*-pyran-2-yl)cinnamamide |  | -5.85 | (Farediah AHMAD, 2020) |
|  | Piperanine |  | -6.32 | (Farediah AHMAD, 2020) |
|  | 3-Phenyl-1-(piperidin-1-yl)propan-1-one |  | -6.20 | (Farediah AHMAD, 2020) |
|  | 3-Chloro-4-hydroxy-2-piperidone |  | -4.56 | (Farediah AHMAD, 2020) |
|  | Alismoxide |  | -5.62 | (Farediah AHMAD, 2020) |
|  | (4*S*,4a*S*,6*S*,8a*S*)-octahydro-4-hydroxy-4,8a-dimethyl-6-(1-methylethenyl)-naphthalen-1(*2H*)-one |  | -5.69 | (Farediah AHMAD, 2020) |
|  | Piperchabaoside A |  | -8.79 | (Farediah AHMAD, 2020) |
|  | (6*S*,9*R*)-Roseoside |  | -7.67 | (Farediah AHMAD, 2020) |
|  | 2-*O*-Methyluridine |  | -6.07 | (Farediah AHMAD, 2020) |
|  | (2*E*,14*Z*)-*N*-isobutyleicosa-2,14-dienamide |  | -6.18 | (Farediah AHMAD, 2020) |
|  | Dipiperamides E |  | -9.79 | (Farediah AHMAD, 2020) |
|  | Dipiperamides F |  | -10.14 | (Farediah AHMAD, 2020) |
|  | Dipiperamides G |  | -10.31 | (Farediah AHMAD, 2020) |
|  | 3,4-Methylenedioxycinnamaldehyde |  | -5.22 | (Farediah AHMAD, 2020) |
|  | Scutifoliamide A |  | -7.49 | (Farediah AHMAD, 2020) |
|  | Pipericine |  | -8.75 | (Farediah AHMAD, 2020) |
|  | (2*E*,4*E*,12*Z*)-*N*-isobutyloctadec-2,4,12-trienamide |  | -6.84 | (Farediah AHMAD, 2020) |
|  | Pipereicosalidine |  | -9.02 | (Farediah AHMAD, 2020) |
|  | Brachystamide B |  | -8.48 | (Farediah AHMAD, 2020) |
|  | Chabamide |  | -10.02 | (Farediah AHMAD, 2020) |
|  | Nigramide F |  | -9.31 | (Farediah AHMAD, 2020) |
|  | Nigramide R |  | -10.18 | (Farediah AHMAD, 2020) |
|  | Piperoctadecalidine |  | -8.42 | (Farediah AHMAD, 2020) |
|  | 3-Methyl-5-decanoylpyridine |  | -6.75 | (Farediah AHMAD, 2020) |
|  | Retrofractosides A |  | -9.80 | (Farediah AHMAD, 2020) |
|  | Retrofractosides B |  | -9.55 | (Farediah AHMAD, 2020) |
|  | Retrofractosides C |  | -9.72 | (Farediah AHMAD, 2020) |
|  | Retrofractosides D |  | -8.89 | (Farediah AHMAD, 2020) |
|  | 3,4-Dihydroxyallylbenzene |  | -5.39 | (Farediah AHMAD, 2020) |
|  | Tachioside |  | -6.37 | (Farediah AHMAD, 2020) |
|  | Benzyl-O-β-D-glucopyranoside |  | -6.17 | (Farediah AHMAD, 2020) |
|  | Icariside F_2_ |  | -7.79 | (Farediah AHMAD, 2020) |
|  | Dihydrovomifoliol-*O-β*-D-glucopyranoside |  | -7.65 | (Farediah AHMAD, 2020) |
|  | Isopropyl *O-β*-D-glucopyranoside |  | -5.44 | (Farediah AHMAD, 2020) |
|  | Isopropyl primeveroside |  | -7.55 | (Farediah AHMAD, 2020) |
|  | *n*-Butyl *O-β*-D-glucopyranoside |  | -5.84 | (Farediah AHMAD, 2020) |
|  | Retrofractosides E |  | -10.51 | (Farediah AHMAD, 2020) |
|  | Retrofractosides F |  | -10.65 | (Farediah AHMAD, 2020) |
|  | Piperoside |  | -7.43 | (Farediah AHMAD, 2020) |
|  | Sesamin |  | -7.20 | (Farediah AHMAD, 2020) |
|  | 3,4,5-Trimethoxydihydrocinnamic acid |  | -5.96 | \| (Farediah AHMAD, 2020) \| \| --- \| |
|  | Benzenepropanoic acid, ethyl ester |  | -6.28 | (Amalia & Nurhariyati, 2020) |
|  | α-Humulene |  | -6.12 | (Amalia & Nurhariyati, 2020) |
|  | Benzenepropanoic acid |  | -5.56 | (Amalia & Nurhariyati, 2020) |
|  | Germacrene-D |  | -6.52 | (Amalia & Nurhariyati, 2020) |
|  | 2H-indene,3,3a,4,5,6,7-hexahydro- |  | -5.07 | (Amalia & Nurhariyati, 2020) |
|  | 2-Furanmethanol |  | -4.21 | (Amalia & Nurhariyati, 2020) |
|  | 1-Octadecene |  | -7.63 | (Amalia & Nurhariyati, 2020) |
|  | 2-oxo-4-phenyl-3-butenoic acid |  | -5.17 | (Amalia & Nurhariyati, 2020) |
|  | Benzyl 2,3,5-trianisoyl-α-d-arabinofuranoside |  | -9.86 | (Amalia & Nurhariyati, 2020) |
|  | Piperine |  | -6.64 | (Amalia & Nurhariyati, 2020) |
|  | D,α-Tocopherol |  | -8.73 | (Amalia & Nurhariyati, 2020) |
|  | Stigmasterol |  | -7.93 | (Amalia & Nurhariyati, 2020) |
|  | (23S)-ethylcholest-5-en-3β-ol |  | **-**8.22 | (Amalia & Nurhariyati, 2020) |
|  | beta-Elemene |  | -6.27 | (Amalia & Nurhariyati, 2020) |
|  | 3-n-Heptyl-7-methyl-9-(2,6,6-trimethylcyclohex-1-enyl)nona-2,4,6,8-tetraenal |  | -8.45 | (Amalia & Nurhariyati, 2020) |
|  | 13-acetyl-2,3,3a,5,6,7,8,13,13a,13b-decahydro-11,12-dimethoxy-2,6-dimethyl-4H-3,8a-Methanofuro[2’,3’:6,7]azonino[5,4-b]indol-14-one |  | Nil | (Amalia & Nurhariyati, 2020) |
|  | Handianol |  | -7.71 | (Amalia & Nurhariyati, 2020) |
|  | Neophytadiene |  | -8.04 | (Amalia & Nurhariyati, 2020) |
|  | 5,12-Naphthacenedione,7-(acetyloxy)8-ethyl-7,8,9,10-tetrahydro-1,4,6,8,11-pentahydroxy- |  | -7.74 | (Amalia & Nurhariyati, 2020) |
|  | 1-(1-Trimethylsiloxyethenyl)-4-trimethylsiloxy-benzene |  | -7.28 | (Amalia & Nurhariyati, 2020) |
|  | *N*-Isobutyl-2*E*,4*E*-dodecadienamide |  | -6.78 | (Sto et al., 2001) |
|  | Piperdardine |  | -6.90 | (Sto et al., 2001) |
|  | Sarmentine |  | -6.96 | (Sto et al., 2001) |
|  | Benzylbenzoate |  | -6.16 | (Sto et al., 2001) |
|  | Brachyamide B |  | -7.36 | (Sto et al., 2001) |
|  | Chingchengenamide |  | -7.12 | (Sto et al., 2001) |
|  | Futoamide |  | -6.61 | (Sto et al., 2001) |
|  | Piperolactam C |  | -7.12 | (Vlad et al., 2021) |
|  | Piperettines I |  | -7.63 | (Vlad et al., 2021) |
|  | Piperettines II |  | -6.46 | (Vlad et al., 2021) |
|  | Piperettines III |  | -6.32 | (Vlad et al., 2021) |
|  | Piperettine IV |  | -7.63 | (Vlad et al., 2021) |
|  | Pipercallosine |  | -7.43 | (Vlad et al., 2021) |
|  | Neopellitorine B |  | -7.01 | (Vlad et al., 2021) |
|  | Brachyamide A |  | -8.11 | (Vlad et al., 2021) |
|  | *N*-Isobutyl-2,4,10,12- octadecatetraenamide |  | -7.09 | (Vlad et al., 2021) |
|  | Safrole |  | -5.61 | (Wang et al., 2022) |
|  | Nerolidol |  | -6.07 | (Wang et al., 2022) |
|  | 3,3-Dimethyl-2-(3-methyl-1,3-  butadienyl)-cyclohexane-1-  methanol |  | -6.12 | (Wang et al., 2022) |
|  | Junenol |  | -6.14 | (Wang et al., 2022) |
|  | Selina-6-en-4-ol |  | -6.12 | (Wang et al., 2022) |
|  | Apitol |  | Nil | (Wang et al., 2022) |
|  | Ledol |  | -5.76 | (Wang et al., 2022) |
|  | alpha-Eudesmol |  | -6.12 | (Wang et al., 2022) |
|  | Methyl eugenol |  | -5.89 | (Wang et al., 2022) |
|  | 2,2-Methylenebis [6-(1,1-  dimethylethyl)-4-methylphenol] |  | -7.33 | (Wang et al., 2022) |
|  | 1H-1,2,3-Triazolo [4,5-c]  quinoline-1-hexanoic acid |  | -7.30 | (Wang et al., 2022) |
|  | 4-Ethyldecane |  | -6.70 | (Wang et al., 2022) |
|  | 8-Methylheptadecane |  | -7.60 | (Wang et al., 2022) |
|  | 5-Methyl-5-propylnonane |  | -6.44 | (Wang et al., 2022) |
|  | 2,3,6-Trimethyldecane |  | -6.24 | (Wang et al., 2022) |
|  | 1-Iodotetradecane |  | -6.85 | (Wang et al., 2022) |
|  | (1alpha,2beta,4beta)-1-Vinyl-1-methyl-2,4-  bis(1-methylvinyl)  cyclohexane |  | -6.24 | (Wang et al., 2022) |
|  | 1-Iodooctadecane |  | -8.18 | (Wang et al., 2022) |
|  | 2-Methylpentadecane |  | -6.97 | (Wang et al., 2022) |
|  | 2,6,10,14-  Tetramethylpentadecane |  | -7.47 | (Wang et al., 2022) |
|  | 9-Octylheptadecane |  | -9.33 | (Wang et al., 2022) |
|  | 1-Iodoeicosane |  | -8.70 | (Wang et al., 2022) |
|  | 2,6,10,14-Tetramethylhexadecane |  | -7.69 | (Wang et al., 2022) |
|  | 2-Methylhexacosane |  | -9.81 | (Wang et al., 2022) |
|  | 1,7,11-Trimethyl-4-(1-  methylethyl)  cyclotetradecane |  | -8.03 | (Wang et al., 2022) |
|  | 3-Methylheptadecane |  | -7.39 | (Wang et al., 2022) |
|  | 1-Iododocosane |  | -8.93 | (Wang et al., 2022) |
|  | Heptacosane |  | -9.28 | (Wang et al., 2022) |
|  | 2,6,10-Trimethyltetradecane |  | -7.10 | (Wang et al., 2022) |
|  | 7,9-Dimethylhexadecane |  | -7.27 | (Wang et al., 2022) |
|  | 3-Methyloctadecane |  | -7.98 | (Wang et al., 2022) |
|  | beta-Copaene |  | -5.99 | (Wang et al., 2022) |
|  | 4-[(1e)-1,5-Dimethyl-1,4-  hexadien-1-yl]-1-methyl cyclohexene |  | -6.67 | (Wang et al., 2022) |
|  | Copaene |  | -5.96 | (Wang et al., 2022) |
|  | beta-Guaiene |  | -6.63 | (Wang et al., 2022) |
|  | 1-Eicosene |  | -7.91 | (Wang et al., 2022) |
|  | 1-Hexacosene |  | -9.38 | (Wang et al., 2022) |
|  | Z-12-Pentacosene |  | -9.21 | (Wang et al., 2022) |
|  | 3-Heptadecene |  | -7.34 | (Wang et al., 2022) |
|  | Methyl benzoate |  | -5.19 | (Wang et al., 2022) |
|  | Triacontyl heptafluorobutyrate |  | -9.59 | (Wang et al., 2022) |
|  | Dibutyl phthalate |  | -7.06 | (Wang et al., 2022) |
|  | 11-Tetradecen-1-ol acetate |  | -7.04 | (Wang et al., 2022) |
|  | Caryophyllene |  | -6.13 | (Chanthasri et al., 2018) |
|  | 1,4,7,-Cycloundecatriene, 1,5,9,9-tetramethyl-,  Z,Z,Z- |  | -6.14 | (Chanthasri et al., 2018) |
|  | 8-Heptadecene |  | -7.46 | (Chanthasri et al., 2018) |
|  | Heptadecane |  | -7.51 | (Chanthasri et al., 2018) |
|  | Isobutyraldehyde |  | -3.97 | (Takahashi et al., 2020) |
|  | 3-Hexanal |  | -4.77 | (Takahashi et al., 2020) |
|  | 2-Hexenal |  | -4.69 | (Takahashi et al., 2020) |
|  | Methanol |  | Nil | (Takahashi et al., 2020) |
|  | 2-Undecanone |  | -6.39 | (Takahashi et al., 2020) |
|  | 2-Heptanol,acetate |  | -6.15 | (Takahashi et al., 2020) |
|  | Sabinene |  | -5.55 | (Takahashi et al., 2020) |
|  | 3-Carene |  | Nil | (Takahashi et al., 2020) |
|  | D-Limonene |  | -5.51 | (Takahashi et al., 2020) |
|  | beta-Phellandrene |  | -5.47 | (Takahashi et al., 2020) |
|  | 1,8-Cineol |  | -5.03 | (Takahashi et al., 2020) |
|  | gamma-Terpinene |  | -5.74 | (Takahashi et al., 2020) |
| 1. s | alpha-Terpinene |  | -5.63 | (Takahashi et al., 2020) |
|  | neo-Alloocimene |  | -5.78 | (Takahashi et al., 2020) |
|  | 1,3,8-Menthatriene |  | -5.35 | (Takahashi et al., 2020) |
|  | Cosmene |  | -5.45 | (Takahashi et al., 2020) |
|  | alpha-Cubebene |  | -6.35 | (Takahashi et al., 2020) |
|  | delta-Elemene |  | -6.29 | (Takahashi et al., 2020) |
|  | alpha-Ylangene |  | -5.96 | (Takahashi et al., 2020) |
|  | beta-Bourbonene |  | -5.76 | (Takahashi et al., 2020) |
|  | beta-Cubebene |  | -5.94 | (Takahashi et al., 2020) |
|  | Longicyclene |  | Nil | (Takahashi et al., 2020) |
|  | alpha-Bergamotene |  | -6.37 | (Takahashi et al., 2020) |
|  | Bicyclosesquiphellandrene |  | -6.33 | (Takahashi et al., 2020) |
|  | Alloaromadendrene |  | -5.76 | (Takahashi et al., 2020) |
|  | gamma-Selinene |  | -5.86 | (Takahashi et al., 2020) |
|  | gamma-Elemene |  | -6.65 | (Takahashi et al., 2020) |
|  | beta-Sesquiphellandrene |  | -6.71 | (Takahashi et al., 2020) |
|  | 1,4-Cadinadiene |  | -6.29 | (Takahashi et al., 2020) |
|  | (-)-Calamenene |  | -6.37 | (Takahashi et al., 2020) |
|  | Undecane |  | -6.25 | (Takahashi et al., 2020) |
|  | 1-Tridecene |  | -6.75 | (Takahashi et al., 2020) |
|  | 3-Methyl-1-butanal |  | -4.49 | (Takahashi et al., 2018) |
|  | Hexanal |  | -4.87 | (Takahashi et al., 2018) |
|  | Ethanol |  | -3.44 | (Takahashi et al., 2018) |
|  | Linalool |  | -6.00 | (Takahashi et al., 2018) |
|  | a-Pinene |  | Nil | (Takahashi et al., 2018) |
|  | Camphene |  | Nil | (Takahashi et al., 2018) |
|  | beta-Pinene |  | -4.61 | (Takahashi et al., 2018) |
|  | beta-Myrcene |  | -5.49 | (Takahashi et al., 2018) |
|  | alpha-Limonene |  | -5.51 | (Takahashi et al., 2018) |
|  | beta-Ocimene |  | -5.73 | (Takahashi et al., 2018) |
|  | alpha-Ocimene |  | -5.78 | (Takahashi et al., 2018) |
|  | Terpinolene |  | -5.45 | (Takahashi et al., 2018) |
|  | Tridecane |  | -6.64 | (Takahashi et al., 2018) |
|  | n-Pentadecane |  | -7.24 | (Takahashi et al., 2018) |
|  | alpha-Copaene |  | -5.96 | (Takahashi et al., 2018) |
|  | trans-beta-Farnesene |  | -6.98 | (Takahashi et al., 2018) |
|  | alpha-Caryophyllene |  | -6.36 | (Takahashi et al., 2018) |
|  | alpha-Cadinene |  | -5.85 | (Takahashi et al., 2018) |
|  | beta-Bisabolene |  | -6.20 | (Takahashi et al., 2018) |
|  | Epizonarene |  | -6.57 | (Takahashi et al., 2018) |
|  | gamma-Muurolene |  | -6.39 | (Takahashi et al., 2018) |
|  | alpha-Selinene |  | -5.98 | (Takahashi et al., 2018) |
|  | (+)-delta-Cadinene |  | -5.90 | (Takahashi et al., 2018) |
|  | gamma-Cadinene |  | -6.25 | (Takahashi et al., 2018) |
|  | alpha-Panasinsene |  | -5.69 | (Takahashi et al., 2018) |
|  | Acetic acid |  | -3.41 | (Takahashi et al., 2018) |
|  | 2-Nonanone |  | -5.88 | (Takahashi et al., 2018) |
|  | Camphor |  | -4.96 | (Takahashi et al., 2018) |
|  | Calarene |  | Nil | (Takahashi et al., 2018) |
|  | Methyl acetate |  | -3.86 | (Takahashi et al., 2018) |
|  | Methyl-3-phenylpropanoate |  | -5.78 | (Takahashi et al., 2018) |
|  | β-sitosterol |  | -8.57 | (Torequl et al., 2020) |
|  | [*SR*,*S’R*]-9-hydroxy,3,4-dimethoxy,3’,4’-methylene dioxy-9,9’ epoxy lignan |  | -7.86 | (Torequl et al., 2020) |
|  | Dihydrop ipercide |  | -7.98 | (Torequl et al., 2020) |
|  | chabamide F |  | -9.12 | (Torequl et al., 2020) |
|  | chabamide G |  | -10.17 | (Torequl et al., 2020) |
|  | Chabamide K |  | -9.03 | (Torequl et al., 2020) |
|  | Trichostachine |  | -6.51 | (Torequl et al., 2020) |
|  | Bornyl piperate |  | -7.45 | (Torequl et al., 2020) |
|  | Piperchabamide F |  | -7.61 | (Torequl et al., 2020) |
|  | piperchabaosides B |  | -9.42 | (Torequl et al., 2020) |
|  | 9-(3’,4’-methylenedioxyphenyl)-nona-2*E*,4*E*,8*E*-trienoic acid |  | -6.68 | (Torequl et al., 2020) |
|  | *n*-butyl amine |  | -4.04 | (Torequl et al., 2020) |
|  | *n*-pentyl amine |  | -4.37 | (Torequl et al., 2020) |
|  | *N*-isobutyl-(2*E*,4*E*)-decadienamide |  | -6.28 | (Torequl et al., 2020) |
|  | piperundecalidiene, |  | -7.71 | (Torequl et al., 2020) |
|  | piperchabamide D |  | -7.66 | (Torequl et al., 2020) |
|  | Troglitazone |  | -8.62 | (Torequl et al., 2020) |

References:

Amalia, S. E., & Nurhariyati, T. (2020). *Identification of phytocemical compounds in ethanol and n-hexane leaf extracts of Piper retrofractum vahl . by gas chromatography mass spectrometry*. *1*, 32–37.

Chanthasri, W., Puangkeaw, N., Kunworarath, N., Jaisamut, P., Limsuwan, S., Maneenoon, K., Choochana, P., & Chusri, S. (2018). *Antioxidant capacities and total phenolic contents of 20 polyherbal remedies used as tonics by folk healers in Phatthalung and Songkhla provinces , Thailand*. 1–11.

Farediah AHMAD, W. M. N. H. W. S. (2020). *Phytopharmacological Investigations of*. *85*(3), 193–202.

Sto, J. R., Xiao, P., & Bauer, R. (2001). *Constituents of Chinese Piper species and their inhibitory activity on prostaglandin and leukotriene biosynthesis in vitro*. *75*, 133–139.

Takahashi, M., Hirose, N., Ohno, S., & Arakaki, M. (2018). Flavor characteristics and antioxidant capacities of hihatsumodoki ( Piper retrofractum Vahl ) fresh fruit at three edible maturity stages. *Journal of Food Science and Technology*, *55*(4), 1295–1305. https://doi.org/10.1007/s13197-018-3040-2

Takahashi, M., Oe, M., Arakaki, M., & Wada, K. (2020). Effect of leaf growth on the taste and aroma functions and antioxidant characteristics of hihatsumodoki ( Piper retrofractum Vhal ) leaf. *Journal of Food Measurement and Characterization*, *14*(2), 1002–1011. https://doi.org/10.1007/s11694-019-00349-4

Torequl, M., Hasan, J., Hossain, H. M. S., Ali, E. S., Sharifi-rad, J., Martorell, M., & Mubarak, M. S. (2020). Chemical profile , traditional uses , and biological activities of Piper chaba Hunter : A review. *Journal of Ethnopharmacology*, *257*(April), 112853. https://doi.org/10.1016/j.jep.2020.112853

Vlad, S., Minceva, M., Gertsch, J., & Skalicka-wo, K. (2021). *LC-HRMS / MS-based phytochemical profiling of Piper spices : Global association of piperamides with endocannabinoid system modulation*. *141*(July 2020). https://doi.org/10.1016/j.foodres.2021.110123

Wang, J., Fan, R., Zhong, Y., Luo, H., & Hao, C. (2022). *Effects of Cabya ( Piper retrofractum Vahl.) Fruit Developmental Stage on VOCs*. 1–11.
