# Supplementary material for: Identification of novel amides and alkaloids as putative inhibitors of dopamine transporter for schizophrenia using computer-aided virtual screening
Source: Front Pharmacol. 2025 Apr 8;16:1509263. doi: 10.3389/fphar.2025.1509263 (PMC12039762; doi:10.3389/fphar.2025.1509263)
Supplement: Supplementary file 4 [file Table8.docx]

**Table S8.** Evaluation of physicochemical properties of the potential hits.

| **Metabolite**  **No.** | **MW** | **Log P** | **Rot. B** | **HBA** | **HBD** | **TPSA** |
| --- | --- | --- | --- | --- | --- | --- |
| * | 393.5 | 3.92 | 5 | 3 | 0 | 26.79 |
|  | 606.66 | 5.36 | 10 | 7 | 3 | 117.56 |
|  | 570.68 | 5.37 | 8 | 6 | 0 | 77.54 |
|  | 544.64 | 4.83 | 7 | 6 | 0 | 77.54 |
|  | 542.62 | 4.60 | 7 | 6 | 0 | 77.54 |
|  | 570.69 | 5.38 | 8 | 6 | 0 | 77.54 |
|  | 532.62 | 3.07 | 7 | 9 | 1 | 128.73 |
|  | 516.85 | 5.55 | 7 | 4 | 0 | 59.08 |
|  | 491.53 | 4.05 | 10 | 7 | 4 | 117.48 |
|  | 570.68 | 5.38 | 7 | 6 | 0 | 77.54 |
|  | 570.68 | 5.38 | 8 | 6 | 0 | 77.54 |

*(MW: Molecular Weight, Rot. B: Rotatable Bond, HBA: Hydrogen Bond Donor, HBD: Hydrogen Bond Acceptor, TPSA: Topological Polar Surface Area, 1: Chenoalbicin, 2: Dipiperamide G, 3: Nigramide R, 4: Chabamide G, 5: Dipiperamide F, 6: 3,12-di-O-acetyl-8-O-tigloylingol, 7: 2,4-Imidazolidinedione,5-[3,4-bis[(trimethylsily)oxy]phenyl]-3-methyl-5-phenyl-1-(trimethylsilyl), 8: Lyciumamide C 9: Chabamide, 10: Dipiperamide E and Asterisk (*): Standard)*
