# Supplementary material for: Identification of novel amides and alkaloids as putative inhibitors of dopamine transporter for schizophrenia using computer-aided virtual screening
Source: Front Pharmacol. 2025 Apr 8;16:1509263. doi: 10.3389/fphar.2025.1509263 (PMC12039762; doi:10.3389/fphar.2025.1509263)
Supplement: Supplementary file 8 [file Table7.docx]

**Table S7.** Docking score and interaction profile of the potential hits from the MOE.

| **Metabolite**  **No.** | **Metabolite**  **Name** | **Docking Score**  **(Kcal/mol)** | **Amino Acids** | **Bond**  **type** | **Bond energy**  **(Kcal/mol)** | **Bond length**  **(Å)** | **Hydrophobic**  **interactions** |
| --- | --- | --- | --- | --- | --- | --- | --- |
|  | Chenoalbicin | -10.45 | ASP475  ARG476 | H-donor  H-acceptor | -2.0  -1.1 | 3.02  3.19 | Lue538 Thr315 Phe319 Tyr124 Asp46 Gly425 Ser422 Phe43 Ser421 Ala117 Val120 Ala479 Gly480 Tyr123 Tyr542 Tyr481 Asp121 Ile483 Ile116 Gly441 |
|  | Dipiperamides G | -10.31 | ASP475  PHE325 | H-donor  pi-pi | -0.7  -0.0 | 3.23  3.67 | Ile483 Ala117 Val120 Trp51 Ala479 Gly480 Ile116 Arg52 Asp46 Ile127 Phe319 Ala48 Gln316 Tyr124 Thr315 Tyr123 Arg476 Leu538 Gly541 |
|  | Nigramide R | -10.18 | ARG476  PHE325 | pi-H  pi-pi | -0.7  -0.0 | 4.17  3.99 | Asp46 Arg52 Thr315 Ala48 Gln316 Asp475 Phe319 Leu538 Tyr481 Ile116 Ala479 Tyr123 Gly480 Val120 Ile483 Ala117 Tyr124 Trp51 Ile127 |
|  | Chabamide G | -10.17 | Nil |  |  |  | Ala48 Tyr124 Ser421 Phe43 Ser422 Gly425 Ser426 Gly418 Tyr123 Asn125 Asp121 Phe325 Phe319 Val120 Ile116 Ile483 Gly480 Ala479 Trp51 Asp46 Asp475 |
|  | Dipiperamide F | -10.14 | Nil |  |  |  | Asp46 Ala479 Tyr123 Ile116 Gly480 Asp475 Arg476 Leu538 Thr315 Phe319 Arg52 Trp51 tyr124 Ser421 Phe43 Val120 Val113 Ala117 Ile483 Phe325 |
|  | 3,12-di-O-acetyl-8-O-tigloylingol | -10.08 | ASP46 | H-donor | -0.7 | 3.44 | Leu47 Phe319 Asp475 Arg476 Leu538 Tyr123 Ile483 Gly480 Phe325Ala479 Ile116 Gly425 Phe43 Ala117 Val120 Ile127 Trp51 Tyr124 Ala48 |
|  | 2,4-Imidazolidinedione,5-[3,4-bis[(trimethylsily)oxy]phenyl]-3-methyl-5-phenyl-1-(trimethylsilyl | -10.04 | Nil |  |  |  | Asp46 Asp475 Ser320 Phe325 Tyr123 Phe43 Tyr124 Ser421 Ile127 Arg52 Leu47 Ala48 Phe319 Trp51 Thr315 Gly480 |
|  | Lyciumamide C | -10.03 | ASP46  PHE319 | H-donor  H-pi | -2.3  -1.2 | 3.22  3.90 | Val120 Ser320 Tyr123 Ala48 Asp475 Tyr124 Ile116 Ala44 Trp51 Ser421 Phe43 Ser422 Gly425 Ala479 Phe325 Ala117 Gly480 Val113 Ile483 Leu321 |
|  | Chabamide | -10.02 | PHE319 | H-pi | -0.8 | 3.83 | Trp51 Gly385 Glu384 Pro386 Arg52 Val120 Ala48 Asp46 Phe43Tyr123 Ser421 Asp475 Tyr124Phe325 Ile483 Arg476 Ala479 Tyr481 Leu538 |
|  | Dipiperamides E | -9.79 | VAL120 | pi-H | -0.6 | 4.32 | Phe325 Phe319 Ala479 Gly480 Lue538 Trp51 Arg52 Asp475 Asp46 Ser421 Tyr123 Tyr124 Arg476 Gly425 Ile116 Phe43 Val327 Ala117 Gly424 |
| * | Lumateperone | -7.57 | PHE319 | pi-pi | -0.0 | 4.00 | Gly480 Ala479 Val120 Phe325 Phe43 Gly425 Tyr124 Ser422 Asp121 Ser421 Asn125 Tyr123 Asp46 Asp475 |
