# Supplementary material for: Identification of novel amides and alkaloids as putative inhibitors of dopamine transporter for schizophrenia using computer-aided virtual screening
Source: Front Pharmacol. 2025 Apr 8;16:1509263. doi: 10.3389/fphar.2025.1509263 (PMC12039762; doi:10.3389/fphar.2025.1509263)
Supplement: Supplementary file 11 [file Table9.docx]

**Table S9.** Evaluation of bioactivity score of the potential hits.

| **Metabolite**  **No.** | **GPCR ligand** | **Ion channel modulator** | **Kinase inhibitor** | **Nuclear receptor ligand** | **Enzyme inhibitor** |
| --- | --- | --- | --- | --- | --- |
| * | 0.29 | 0.08 | -0.00 | 0.03 | 0.04 |
| 1 | -0.07 | -0.76 | -0.57 | -0.48 | -0.38 |
| 2 | 0.05 | -0.46 | -0.25 | -0.27 | -0.13 |
| 3 | 0.07 | -0.33 | -0.20 | -0.12 | -0.06 |
| 4 | 0.15 | -0.37 | -0.26 | -0.08 | -0.06 |
| 5 | 0.06 | -0.48 | -0.29 | -0.18 | -0.20 |
| 6 | -0.08 | -0.08 | -0.34 | 0.31 | 0.26 |
| 7 | 0.26 | 0.12 | -0.09 | -0.09 | 0.62 |
| 8 | 0.09 | -0.07 | -0.14 | 0.10 | 0.02 |
| 9 | 0.08 | -0.53 | -0.38 | -0.26 | -0.14 |
| 10 | 0.06 | -0.48 | -0.29 | -0.18 | -0.20 |

*(1: Chenoalbicin, 2: Dipiperamide G, 3: Nigramide R, 4: Chabamide G, 5: Dipiperamide F, 6: 3,12-di-O-acetyl-8-O-tigloylingol, 7: 2,4-Imidazolidinedione,5-[3,4-bis[(trimethylsily)oxy]phenyl]-3-methyl-5-phenyl-1-(trimethylsilyl), 8: Lyciumamide C 9: Chabamide, 10: Dipiperamide E and Asterisk (*): Standard)*
