# Supplementary material for: Identification of novel amides and alkaloids as putative inhibitors of dopamine transporter for schizophrenia using computer-aided virtual screening
Source: Front Pharmacol. 2025 Apr 8;16:1509263. doi: 10.3389/fphar.2025.1509263 (PMC12039762; doi:10.3389/fphar.2025.1509263)
Supplement: Supplementary file 12 [file Table2.docx]

**Table S2.** Library of the secondary metabolites of *Euphorbia neriifolia L.*

| **Sr.**  **No.** | **Compound** | **Structure** | **Docking value**  **(Kcal/mol)** | **References** |
| --- | --- | --- | --- | --- |
|  | 2-(3,4-dihydroxy-5-methoxy  phenyl)- 3,5-dihydroxy-6,7-dimethoxychromen-4-one |  | -7.44 | (Sharma & Janmeda, 2017) |
|  | 3-Methyl-agallochaol C |  | -7.41 | (Technologyn, 2010) |
|  | Ent-3-beta, (13S)-dihydroxyatis-16-en-14-one |  | -6.48 | (Technologyn, 2010) |
|  | Ent-16-alpha, 17-dihydroxyatisan-3-one |  | -6.84 | (Technologyn, 2010) |
|  | Ent-atisane-3-beta, 16-alpha,17-triol |  | Nil | (Technologyn, 2010) |
|  | 4, 13-beta-dihydroxy-14-oxo-3, 4-secoatis-16-en-3-oic acid methyl ester |  | -7.88 | (Technologyn, 2010) |
|  | 13-beta, 19-dihydroxy-3,15-dioxoatis-16-ene |  | -6.94 | (Technologyn, 2010) |
|  | 13-beta-hydroxy-3, 15-dioxoatis-16-ene |  | -6.40 | (Technologyn, 2010) |
|  | Ent-16-alpha, 17-dihydroxykauran-3-one |  | -6.51 | (Technologyn, 2010) |
|  | 3-acetoxymethyl-5-[(E)-3-acetoxy-propen-1-yl)]-2-(4-hydroxy-3-methoxyphenyl)-7-methoxy-2,3-dihydro benzofuran |  | -8.83 | (Technologyn, 2010) |
|  | Taraxerol |  | -8.04 | (Technologyn, 2010) |
|  | 9-beta, 19-cyclolanostan-3-beta-ol |  | Nil | (Technologyn, 2010) |
|  | 6,7,8-trimethoxyl-coumarin |  | -6.42 | (Technologyn, 2010) |
|  | 3,3’-di-O-methylellagic acid |  | -6.94 | (Technologyn, 2010) |
|  | 9,19-cyclolanost-22 (22’),24-diene-3-beta-ol (Neriifoliene) |  | Nil | (Mali & Panchal, 2017) |
|  | Euphol |  | -8.67 | (Mali & Panchal, 2017) |
|  | Euphorbol hexacosanoate |  | -12.99 | (Mali & Panchal, 2017) |
|  | Neriifolione |  | -8.33 | (Mali & Panchal, 2017) |
|  | Cycloartenol |  | -8.29 | (Mali & Panchal, 2017) |
|  | Neriifoliol |  | -7.65 | (Mali & Panchal, 2017) |
|  | 3-O-acetyl-8-O-tigloylingol |  | -9.42 | (Mali & Panchal, 2017) |
|  | 3,12-di-O-acetyl-8-O-tigloylingol |  | -10.08 | (Mali & Panchal, 2017) |
|  | (24R)-cycloartane-  3-beta-,24,25-triol |  | -8.68 | (Mali & Panchal, 2017) |
|  | 5,4’-dihydroxy-3,7,3’,5’-tetramethoxyflavone |  | -7.35 | (Mali & Panchal, 2017) |
|  | pachypodol (5,40-dihydroxy-3,7,30-trimethoxyflavone) |  | -7.99 | (Mali & Panchal, 2017) |
|  | Combretol |  | -7.84 | (Mali & Panchal, 2017) |
|  | Friedelan 3-alpha- ol |  | -7.25 | (Mali & Panchal, 2017) |
|  | Friedelan 3-beta-ol |  | Nil | (Mali & Panchal, 2017) |
|  | Glut-5 (10)-en-1-one |  | -7.63 | (Mali & Panchal, 2017) |
|  | Quercetin |  | -6.23 | (Mali & Panchal, 2017) |
|  | n-hexacosanol |  | -9.23 | (Mali & Panchal, 2017) |
|  | Antiquorin |  | -6.76 | (Mali & Panchal, 2017) |
|  | delphinidin-3,5-diglucoside |  | -10.24 | (Ahmed & Fatima, 2018) |
|  | Euphorbol |  | -9.02 | (Ahmed & Fatima, 2018) |
|  | Hexacosanoate |  | -9.38 | (Ahmed & Fatima, 2018) |
|  | pelargonin-3,5- diglucoside |  | -9.93 | (Ahmed & Fatima, 2018) |
|  | Nerifoliol |  | -8.54 | (Ahmed & Fatima, 2018) |
|  | Neriifolin A |  | Nil | (Choodej & Pudhom, 2020) |
|  | Neriifolin B |  | -7.35 | (Choodej & Pudhom, 2020) |
|  | Neriifolin C |  | Nil | (Choodej & Pudhom, 2020) |
|  | 24-methylenecycloartane |  | Nil | (Choodej & Pudhom, 2020) |
|  | *Eupneria J* |  | -6.48 | (Li et al., 2020) |
|  | *Eupneria K* |  | -6.58 | (Li et al., 2020) |
|  | *Eupneria L* |  | -6.43 | (Li et al., 2020) |
|  | *Eupneria M* |  | -6.64 | (Li et al., 2020) |
|  | *Eupneria N* |  | -6.77 | (Li et al., 2020) |
|  | *Eupneria O* |  | -7.09 | (Li et al., 2020) |
|  | *Eupneria P* |  | -6.75 | (Li et al., 2020) |
|  | oryzalexin F |  | Nil | (Li et al., 2020) |
|  | *ent*-isopimara-8(14),15-dien-3*β*,12*β*-diol |  | -6.49 | (Li et al., 2020) |
|  | 3*α*,12*α*-dihydroxy-*ent*-8(14),15-isopimaradien-18-  Al |  | Nil | (Li et al., 2020) |
|  | Euphnerin A |  | -6.56 | (Du et al., 2020) |
|  | euphnerin B |  | -6.47 | (Du et al., 2020) |
|  | euphominoid E  (ent-rosane diterpenoid) |  | -6.99 | (Du et al., 2020) |
|  | 2-Methylthiolane,  S,S-dioxide |  | -4.74 | (Kumar et al., 2021) |
|  | Cyclohexene,  1-methyl-4-(1-  methylethenyl)-,  (s)- |  | -5.50 | (Kumar et al., 2021) |
|  | Vitamin A Aldehyde |  | -7.24 | (Kumar et al., 2021) |
|  | Dodecane,  1-Fluoro- |  | -6.89 | (Kumar et al., 2021) |
|  | Octadecane,  1-chloro- |  | -8.06 | (Kumar et al., 2021) |
|  | 3-beta-friedelanol |  | Nil | (Sultana et al., 2022) |
|  | 3-beta-acetoxy  Friedelane |  | Nil | (Sultana et al., 2022) |
|  | Friedelin |  | -6.99 | (Sultana et al., 2022) |
|  | Lupenone |  | -8.24 | (Sultana et al., 2022) |
|  | Epitaraxerol |  | -7.87 | (Sultana et al., 2022) |
|  | Epitaraxeryl acetate |  | Nil | (Sultana et al., 2022) |
|  | Taraxeryl acetate |  | -8.91 | (Sultana et al., 2022) |
|  | beta-amyrin |  | -7.74 | (Sultana et al., 2022) |
|  | 3-beta-simiarenol |  | -8.01 | (Sultana et al., 2022) |
|  | Cycloeucalenol |  | -7.92 | (Sultana et al., 2022) |
|  | Afzelin |  | -8.38 | (Sultana et al., 2022) |
|  | Euphonerin A |  | -8.68 | (Sultana et al., 2022) |
|  | Euphonerin B |  | -8.28 | (Sultana et al., 2022) |
|  | Euphonerin C |  | Nil | (Sultana et al., 2022) |
|  | Euphonerin D |  | -9.38 | (Sultana et al., 2022) |
|  | 24-  Methylenecycloarenol |  | -8.34 | (Sultana et al., 2022) |
|  | 12-Deoxyphorbol-  13,20-diacetate |  | -8.49 | (Sultana et al., 2022) |
|  | Ingenol triacetate |  | -7.48 | (Sultana et al., 2022) |
|  | Tulipanin |  | -10.22 | (Sultana et al., 2022) |
|  | Euphonerin E |  | -9.04 | (Sultana et al., 2022) |
|  | Euphonerin F |  | -8.49 | (Sultana et al., 2022) |
|  | Euphonerin G |  | -9.60 | (Sultana et al., 2022) |
|  | Glutinone |  | -7.12 | (Sultana et al., 2022) |
|  | Glutinol acetate |  | -7.68 | (Sultana et al., 2022) |
|  | Glutin-5-en-3-beta-ol |  | -7.59 | (Sultana et al., 2022) |
|  | Dammarenediol II acetate |  | -9.43 | (Sultana et al., 2022) |
|  | Beta-amyrin acetate |  | -8.19 | (Sultana et al., 2022) |
|  | Cabraleadiol monoacetate |  | -9.09 | (Sultana et al., 2022) |
|  | Simiarenone |  | -8.08 | (Sultana et al., 2022) |
|  | Cycloartanol |  | -7.98 | (Sultana et al., 2022) |
|  | 29-norcycloartanol |  | -8.83 | (Sultana et al., 2022) |
|  | (23Z) cycloart-23-ene-3I,25-diol |  | Nil | (Sultana et al., 2022) |
|  | 24-oxocycloart-25-en-3-ol |  | Nil | (Sultana et al., 2022) |
|  | Phorneroid A |  | -7.04 | (Gao et al., 2022) |
|  | Phorneroid B |  | Nil | (Gao et al., 2022) |
|  | phorneroid C |  | -7.62 | (Gao et al., 2022) |
|  | Phorneroid D |  | -6.75 | (Gao et al., 2022) |
|  | Phorneroid E |  | -5.97 | (Gao et al., 2022) |
|  | Phorneroid F |  | -6.57 | (Gao et al., 2022) |
|  | phorneroid G |  | Nil | (Gao et al., 2022) |
|  | Phorneroid H |  | Nil | (Gao et al., 2022) |
|  | Phorneroid I |  | -6.39 | (Gao et al., 2022) |
|  | Phorneroid J |  | Nil | (Gao et al., 2022) |
|  | phorneroid K |  | -7.83 | (Gao et al., 2022) |
|  | Phorneroid L |  | -6.98 | (Gao et al., 2022) |
|  | Phorneroid M |  | -6.88 | (Gao et al., 2022) |
|  | 17-hydroxyjolkinolide B |  | Nil | (Gao et al., 2022) |
|  | Euphorantin S |  | -8.06 | (Yan et al., 2018) |
|  | Euphorantin T |  | Nil | (Yan et al., 2018) |
|  | Euphorneroid A |  | -7.58 | (Yan et al., 2018) |
|  | euphorneroid B |  | Nil | (Yan et al., 2018) |
|  | Euphorneroid C |  | Nil | (Yan et al., 2018) |
|  | Euphorneroid D |  | -7.09 | (Yan et al., 2018) |
|  | 12-O-acetyl-8-O-methylingol |  | -8.59 | (Yan et al., 2018) |
|  | ent-3-oxoatisan-16-alpha,17-  acetonide |  | -7.36 | (Yan et al., 2018) |
|  | ent-18-dihydroxyatis-16-ene-  3,14-dione |  | Nil | (Yan et al., 2018) |
|  | ent-3-beta,13R-dihydroxyatis-16-en-14-  one |  | -6.30 | (Yan et al., 2018) |
|  | ent-3-alpha-hydroxyatis-16-en-14-one |  | Nil | (Yan et al., 2018) |
|  | 3,8,12-O-triacetylingol-  7-benzoate |  | -10.03 | (Yan et al., 2018) |
|  | ingol-3,8,12-O-triacetyl-  7-tiglate |  | -9.45 | (Yan et al., 2018) |
|  | antiquorine A | V | -6.33 | (Yan et al., 2018) |
|  | *Eupneria A* |  | -6.82 | (Li et al., 2019) |
|  | *Eupneria E* |  | -6.53 | (Li et al., 2019) |
|  | *Eupneria B* |  | -7.07 | (Li et al., 2019) |
|  | *Eupneria C* |  | -6.72 | (Li et al., 2019) |
|  | *Eupneria D* |  | Nil | (Li et al., 2019) |
|  | *Eupneria F* |  | -6.82 | (Li et al., 2019) |
|  | 1*α*,3*α*-dihydroxy-*ent-*abieta-  8(14),13(15)-dien-16,12-olide |  | -7.14 | (Li et al., 2019) |
|  | Eurifoloid A |  | -10.53 | (J. X. Zhao et al., 2014) |
|  | Eurifoloid B |  | -8.32 | (J. X. Zhao et al., 2014) |
|  | Eurifoloid C |  | Nil | (J. X. Zhao et al., 2014) |
|  | Eurifoloid D |  | Nil | (J. X. Zhao et al., 2014) |
|  | Eurifoloid E |  | -7.29 | (J. X. Zhao et al., 2014) |
|  | Eurifoloid F |  | -6.76 | (J. X. Zhao et al., 2014) |
|  | Eurifoloid G |  | Nil | (J. X. Zhao et al., 2014) |
|  | Eurifoloid H |  | -7.52 | (J. X. Zhao et al., 2014) |
|  | Eurifoloid I |  | -6.53 | (J. X. Zhao et al., 2014) |
|  | Eurifoloid J |  | -7.56 | (J. X. Zhao et al., 2014) |
|  | Eurifoloid K |  | Nil | (J. X. Zhao et al., 2014) |
|  | Eurifoloid L |  | Nil | (J. X. Zhao et al., 2014) |
|  | Eurifoloid M |  | -6.51 | (J. X. Zhao et al., 2014) |
|  | Eurifoloid N |  | Nil | (J. X. Zhao et al., 2014) |
|  | Eurifoloid O |  | -6.37 | (J. X. Zhao et al., 2014) |
|  | Eurifoloid P |  | Nil | (J. X. Zhao et al., 2014) |
|  | Eurifoloid Q |  | -6.66 | (J. X. Zhao et al., 2014) |
|  | Eurifoloid R |  | -7.53 | (J. X. Zhao et al., 2014) |
|  | Eupneria G |  | -6.45 | (H. Zhao et al., 2022) |
|  | eupneria H |  | Nil | (H. Zhao et al., 2022) |
|  | Eupneria I |  | -8.04 | (H. Zhao et al., 2022) |

**References:**

Ahmed, S., & Fatima, L. (2018). *Pharmacological actions and therapeutic benefits of thuhar ( Euphorbia neriifolia ): A review*. *7*(9), 221–226.

Choodej, S., & Pudhom, K. (2020). Cycloartane triterpenoids from the leaves of Euphorbia neriifolia. *Phytochemistry Letters*, *35*(July 2019), 1–5. https://doi.org/10.1016/j.phytol.2019.10.005

Du, M., An, L., Xu, J., & Guo, Y. (2020). Euphnerins A and B, diterpenoids with a 5/6/6 rearranged spirocyclic carbon skeleton from the stems of Euphorbia neriifolia. *Journal of Natural Products*, *83*(9), 2592–2596. https://doi.org/10.1021/acs.jnatprod.0c00249

Gao, Y., Zhou, J. S., Liu, H. C., Zhang, Y., Yin, W. H., Liu, Q. F., Wang, G. W., Zhao, J. X., & Yue, J. M. (2022). Phorneroids A–M, diverse types of diterpenoids from Euphorbia neriifolia. *Phytochemistry*, *198*(November 2021). https://doi.org/10.1016/j.phytochem.2022.113142

Kumar, A., Mahanty, B., Goswami, R. C. D., Barooah, P. K., & Choudhury, B. (2021). In vitro antidiabetic, antioxidant activities and GC–MS analysis of Rhynchostylis Retusa and Euphorbia Neriifolia leaf extracts. *3 Biotech*, *11*(7), 1–10. https://doi.org/10.1007/s13205-021-02869-7

Li, J. C., Dai, W. F., Liu, D., Jiang, M. Y., Zhang, Z. J., Chen, X. Q., Chen, C. H., Li, R. T., & Li, H. M. (2020). Bioactive ent-isopimarane diterpenoids from Euphorbia neriifolia. *Phytochemistry*, *175*(April). https://doi.org/10.1016/j.phytochem.2020.112373

Li, J. C., Zhang, Z. J., Yang, T., Jiang, M. Y., Liu, D., Li, H. M., & Li, R. T. (2019). Six new ent-abietane-type diterpenoids from the stem bark of Euphorbia neriifolia. *Phytochemistry Letters*, *34*(August), 13–17. https://doi.org/10.1016/j.phytol.2019.09.003

Mali, P. Y., & Panchal, S. S. (2017). Euphorbia neriifolia L.: Review on botany, ethnomedicinal uses, phytochemistry and biological activities. *Asian Pacific Journal of Tropical Medicine*, *10*(5), 430–438. https://doi.org/10.1016/j.apjtm.2017.05.003

Sharma, V., & Janmeda, P. (2017). Extraction, isolation and identification of flavonoid from Euphorbia neriifolia leaves. *Arabian Journal of Chemistry*, *10*(4), 509–514. https://doi.org/10.1016/j.arabjc.2014.08.019

Sultana, A., Hossain, M. J., Kuddus, M. R., Rashid, M. A., Zahan, M. S., Mitra, S., Roy, A., Alam, S., Sarker, M. M. R., & Mohamed, I. N. (2022). Ethnobotanical Uses, Phytochemistry, Toxicology, and Pharmacological Properties of Euphorbia neriifolia Linn. against Infectious Diseases: A Comprehensive Review. *Molecules*, *27*(14). https://doi.org/10.3390/molecules27144374

Technologyn, I. (2010). *J 9] [10].* 183–188.

Yan, S. L., Li, Y. H., Chen, X. Q., Liu, D., Chen, C. H., & Li, R. T. (2018). Diterpenes from the stem bark of Euphorbia neriifolia and their in vitro anti-HIV activity. *Phytochemistry*, *145*, 40–47. https://doi.org/10.1016/j.phytochem.2017.10.006

Zhao, H., Sun, L., Kong, C. H., Mei, W. L., Dai, H. F., Xu, F. Q., & Huang, S. Z. (2022). Phytochemical and pharmacological review of diterpenoids from the genus Euphorbia Linn (2012–2021). *Journal of Ethnopharmacology*, *298*(May). https://doi.org/10.1016/j.jep.2022.115574

Zhao, J. X., Liu, C. P., Qi, W. Y., Han, M. L., Han, Y. S., Wainberg, M. A., & Yue, J. M. (2014). Eurifoloids A-R, structurally diverse diterpenoids from euphorbia neriifolia. *Journal of Natural Products*, *77*(10), 2224–2233. https://doi.org/10.1021/np5004752
