# Supplementary material for: Identification of novel amides and alkaloids as putative inhibitors of dopamine transporter for schizophrenia using computer-aided virtual screening
Source: Front Pharmacol. 2025 Apr 8;16:1509263. doi: 10.3389/fphar.2025.1509263 (PMC12039762; doi:10.3389/fphar.2025.1509263)
Supplement: Supplementary file 13 [file Table5.docx]

**Table S5.** Library of the secondary metabolites of *Vitex negundo* L.

| **Sr.**  **No.** | **Compound** | **Structure** | **Docking value**  **(Kcal/mol)** | **References** |
| --- | --- | --- | --- | --- |
|  | D-Mannose |  | -4.65 | (Kumar et al., 2010) |
|  | Butane, 1,1-diethoxy-3-methyl |  | -4.81 | (Kumar et al., 2010) |
|  | Hexanoic acid, ethyl ester |  | -5.36 | (Kumar et al., 2010) |
|  | Propane, 1,1,3-triethoxy |  | -5.31 | (Kumar et al., 2010) |
|  | 2,3-Dihydrothiophene 1,1-dioxide |  | -4.28 | (Kumar et al., 2010) |
|  | 4H-Pyran-4-one, 2,3-dihydro-3,5-dihydroxy-6-methyl |  | -4.62 | (Kumar et al., 2010) |
|  | 2,4-Pentadien-1-ol, 3-propyl-, (2Z)- |  | -4.59 | (Kumar et al., 2010) |
|  | D-Glucose, 6-O-_-D-galactopyranosyl- |  | -6.72 | (Kumar et al., 2010) |
|  | Ascaridole epoxide |  | Nil | (Kumar et al., 2010) |
|  | 4,9-Decadienoic acid, 2-nitro-, ethyl ester |  | -6.47 | (Kumar et al., 2010) |
|  | Hexadecanoic acid, ethyl ester |  | -7.51 | (Kumar et al., 2010) |
|  | 10, 13-Octadecadiynoic acid, methyl ester |  | -7.73 | (Kumar et al., 2010) |
|  | 4-Decynoic acid, methyl ester |  | -5.78 | (Kumar et al., 2010) |
|  | Azulene, 1,4-dimethyl-7-(1-methylethyl)- |  | -6.19 | (Kumar et al., 2010) |
|  | Ethanol, 2-(9-octadecenyloxy)-, (Z)- |  | -8.15 | (Kumar et al., 2010) |
|  | Aucubin |  | -7.16 | (Kumar et al., 2010) |
|  | Aromadendrene oxide-(1) |  | -5.76 | (Kumar et al., 2010) |
|  | Phytol |  | -7.52 | (Kumar et al., 2010) |
|  | Ethanol, 2-(9,12-octadecadienyloxy)-, (Z,Z)- |  | -8.31 | (Kumar et al., 2010) |
|  | 12-Bromo-13-hydroxy-2,5,9,13 tetramethyltetradeca-4,8-dienoic acid, methyl ester |  | -7.00 | (Kumar et al., 2010) |
|  | 6,9,12,15-  Docosatetraenoic acid,  methyl ester |  | -8.61 | (Kumar et al., 2010) |
|  | Plumbagin |  | -5.51 | (Meena et al., 2022) |
|  | Apocynin |  | -5.21 | (Meena et al., 2022) |
|  | β-Asarone |  | -6.36 | (Meena et al., 2022) |
|  | Imperatorin |  | -6.16 | (Meena et al., 2022) |
|  | Coumarin |  | -4.62 | (Meena et al., 2022) |
|  | Luteolin-3′ ,7-Diglucoside |  | -9.19 | (Meena et al., 2022) |
|  | 4,5-Dicaffeoylquinic acid |  | -9.09 | (Meena et al., 2022) |
|  | Isoquercetin |  | -7.98 | (Meena et al., 2022) |
|  | 7-Hydroxycoumarine |  | -5.17 | (Meena et al., 2022) |
|  | Quercitrin |  | -8.67 | (Meena et al., 2022) |
|  | 4-Methylumbelliferone |  | -5.22 | (Meena et al., 2022) |
|  | (− )-alpha-Santonin |  | -6.28 | (Meena et al., 2022) |
|  | (E)− 4-Methoxycinnamic acid |  | -10.93 | (Meena et al., 2022) |
|  | (-)-Lupinine |  | -5.02 | (Meena et al., 2022) |
|  | Apigenin triacetate |  | -7.56 | (Meena et al., 2022) |
|  | α-Lapachone |  | -5.93 | (Meena et al., 2022) |
|  | Thymol |  | -5.11 | (Meena et al., 2022) |
|  | Retusin (flavonol) |  | -8.04 | (Meena et al., 2022) |
|  | Asiatic acid |  | -8.03 | (Meena et al., 2022) |
|  | (+/-)-Eucalyptol |  | -4.45 | (Meena et al., 2022) |
|  | (+)-[6]-Gingerol |  | -7.65 | (Meena et al., 2022) |
|  | Diosgenin |  | -7.72 | (Meena et al., 2022) |
|  | Oleic acid |  | -7.46 | (Meena et al., 2022) |
|  | Betulin |  | -7.880 | (Meena et al., 2022) |
|  | Erucamide |  | -8.29 | (Meena et al., 2022) |
|  | Stigmasterol acetate |  | -8.67 | (Meena et al., 2022) |
|  | 1,30-Triacontanediol |  | -9.63 | (Meena et al., 2022) |
|  | Agmatine |  | -4.36 | (Meena et al., 2022) |
|  | L-(-)-Asparagine |  | -6.04 | (Meena et al., 2022) |
|  | 4-Nitroaniline |  | -4.62 | (Meena et al., 2022) |
|  | 4-Aminobenzoic acid |  | -4.99 | (Meena et al., 2022) |
|  | Abietin |  | -7.14 | (Meena et al., 2022) |
|  | Quinine |  | -6.51 | (Meena et al., 2022) |
|  | Kaempferol-7-O-glucoside |  | -8.23 | (Meena et al., 2022) |
|  | Ambrosic acid |  | -6.01 | (Meena et al., 2022) |
|  | Lapachol |  | -5.87 | (Meena et al., 2022) |
|  | 1-Adamantano |  | Nil | (Meena et al., 2022) |
|  | 10-Gingerol |  | -7.92 | (Meena et al., 2022) |
|  | 4-Hydroxycoumarin |  | -5.13 | (Meena et al., 2022) |
|  | (E)-parinaric acid |  | -7.34 | (Meena et al., 2022) |
|  | Lup-20(29)-en-28-al, 3beta‑hydroxy- |  | -7.47 | (Meena et al., 2022) |
|  | Lupa-13(18),20(29)‑dien-3-yl acetate |  | -8.93 | (Meena et al., 2022) |
|  | (22E)-Stigmasta-3,5,22-triene |  | -8.31 | (Meena et al., 2022) |
|  | Lupa-12,20(29)‑dien-3-one |  | -7.49 | (Meena et al., 2022) |
|  | (22E)-Stigmasta-5,22‑dien-3-ol |  | -9.51 | (Meena et al., 2022) |
|  | Pentafluoropropionic acid, octadecyl ester |  | Nil | (Meena et al., 2022) |
|  | Sulfurous acid, dodecyl 2-propyl ester |  | -7.40 | (Meena et al., 2022) |
|  | Nonadecane, 2-methyl |  | -7.25 | (Meena et al., 2022) |
|  | Dotriacontane |  | -10.14 | (Meena et al., 2022) |
|  | 1-Octanol, 2-Butyl |  | -6.02 | (Meena et al., 2022) |
|  | Sulfurous acid, pentadecyl 2-propyl ester |  | -8.09 | (Meena et al., 2022) |
|  | 4-Tetradecanol |  | -6.18 | (Meena et al., 2022) |
|  | Trichloromethane |  | -3.48 | (Meena et al., 2022) |
|  | Sulfurous acid, 2-propyl tetradecyl ester |  | -8.04 | (Meena et al., 2022) |
|  | Sulfurous acid, 2-propyl tridecyl ester |  | -7.77 | (Meena et al., 2022) |
|  | Eicosane, 9-Octyl |  | -9.86 | (Meena et al., 2022) |
|  | Di-n-Decylsulfone |  | -8.32 | (Meena et al., 2022) |
|  | 5-Hydroxy-7, 4′-dimethoxy flavones |  | -6.61 | (Meena et al., 2022) |
|  | 5,7-Dihydroxy-6,4′-dimethoxy flavones |  | -6.69 | (Meena et al., 2022) |
|  | Luteolin-7-O-β-D-glucoside |  | -8.21 | (Meena et al., 2022) |
|  | 7,8-Di methyl herbacetin-3-rhamnoside |  | -8.64 | (Meena et al., 2022) |
|  | Vitegnoside |  | -8.11 | (Meena et al., 2022) |
|  | Iso-orientin |  | -7.91 | (Meena et al., 2022) |
|  | Chrysoplenetin |  | -7.18 | (Meena et al., 2022) |
|  | Chrysosplenol D |  | -7.17 | (Meena et al., 2022) |
|  | 4′,5-Dihydroxy-3, 6, 7-trimethoxyflavone |  | -10.05 | (Meena et al., 2022) |
|  | 5, 3′-Hydroxy-6, 7, 4′-trimethoxy flavone |  | -7.25 | (Meena et al., 2022) |
|  | 5, 7, 3′-Trihydroxy-6, 8, 4′-trimethoxy  Flavone |  | -7.40 | (Meena et al., 2022) |
|  | Acerosin-5-O-glucoside |  | -8.99 | (Meena et al., 2022) |
|  | Corymbosin |  | -6.99 | (Meena et al., 2022) |
|  | 5-Hydroxy-6, 7, 8, 3′,  4′-pentamethoxyflavone |  | -6.66 | (Meena et al., 2022) |
|  | 5, 6, 7, 8, 3′, 4′, 5′-Heptamethoxy  Flavone |  | -11.70 | (Meena et al., 2022) |
|  | Casticin |  | -7.36 | (Meena et al., 2022) |
|  | 5, 3′-Hydroxy-7, 8,  4′-trimethoxyflavone |  | -7.30 | (Meena et al., 2022) |
|  | 3,6,7,3′,4′-Pentamethoxyflavone-  5-O-glucopyransylrhamnoside |  | -9.73 | (Meena et al., 2022) |
|  | Vitexincafeate |  | -10.096 | (Meena et al., 2022) |
|  | 5-Hydroxy-3,6,7,3′,4′-pentamethoxy  Flavone |  | -7.86 | (Meena et al., 2022) |
|  | Vitexicarpin |  | -7.44 | (Meena et al., 2022) |
|  | 4′-O-methylmyricetin-3-O-[4′′-O-β-Dgalactosyl]-  β-D-galactopyranoside |  | -9.63 | (Meena et al., 2022) |
|  | 3,4,5,7,3′,4′,5′-Hexahydroxy-6,8-  Dimethoxyflavone |  | -7.23 | (Meena et al., 2022) |
|  | 4,5-Dihydroxy-3′,4′-dimethoxyflavone-  6-O-rhamnoglucoside |  | -7.42 | (Meena et al., 2022) |
|  | 5, 7-Dihydroxychromone |  | -4.95 | (Meena et al., 2022) |
|  | Coniferyl aldehyde |  | -4.63 | (Meena et al., 2022) |
|  | Trans-3,5-dimethoxy-4-hydroxycinnamic  Aldehyde |  | -5.48 | (Meena et al., 2022) |
|  | 2-Methoxy-4-(3-methoxy-1-propenyl)-phenol |  | -6.12 | (Meena et al., 2022) |
|  | Matairesionl |  | -7.50 | (Meena et al., 2022) |
|  | Vitrofolal E |  | -6.79 | (Meena et al., 2022) |
|  | Vitexdoin B |  | -6.55 | (Meena et al., 2022) |
|  | Vitrofolal F |  | -6.76 | (Meena et al., 2022) |
|  | Vitexdoin E |  | -7.30 | (Meena et al., 2022) |
|  | Vitexdoin C |  | -7.33 | (Meena et al., 2022) |
|  | Vitexdoin D |  | -7.45 | (Meena et al., 2022) |
|  | Negundin A |  | -7.64 | (Meena et al., 2022) |
|  | Detetrahydroconidendrin |  | -7.37 | (Meena et al., 2022) |
|  | Vitedoamine A |  | -7.25 | (Meena et al., 2022) |
|  | Vitedoamine B |  | -7.26 | (Meena et al., 2022) |
|  | Vitexdoin I |  | -7.21 | (Meena et al., 2022) |
|  | Negundin B |  | -7.54 | (Meena et al., 2022) |
|  | Vitedoin A |  | -6.97 | (Meena et al., 2022) |
|  | 6-Hydroxy-4-(4-hydroxy-3-methoxyphenyl)-  3-hydroxymethyl-7-methoxy-  3,4-dihydro-2-naphthaldehyde |  | -7.22 | (Meena et al., 2022) |
|  | Vitexdoin A |  | -7.66 | (Meena et al., 2022) |
|  | Vitexdoin G |  | -9.79 | (Meena et al., 2022) |
|  | Vitexdoin H |  | -10.90 | (Meena et al., 2022) |
|  | (+)-Lyoniresinol |  | -7.32 | (Meena et al., 2022) |
|  | (+)-Lyoniresinol-3α-O-β-D-glucoside |  | -7.68 | (Meena et al., 2022) |
|  | Vitexdoin F |  | -6.76 | (Meena et al., 2022) |
|  | 2α,3β-7-O-methylcedrusin |  | -7.64 | (Meena et al., 2022) |
|  | Vitelignin A |  | -8.40 | (Meena et al., 2022) |
|  | 4-Oxosesamin |  | -7.19 | (Meena et al., 2022) |
|  | (+)-Sesamin |  | -7.09 | (Meena et al., 2022) |
|  | (+)-Paulownin |  | -6.99 | (Meena et al., 2022) |
|  | 4-Hydroxysesamin |  | -7.57 | (Meena et al., 2022) |
|  | 4,8-Dihydroxysesamin |  | -7.72 | (Meena et al., 2022) |
|  | 4-Oxopaulownin |  | -9.28 | (Meena et al., 2022) |
|  | (+)-2-(3-Methoxy-4-hydroxyphenyl)-  6-(3,4-methylenedioxy)phenyl-3,7-  dioxabicyclo[3.3.0]octane |  | -7.82 | (Meena et al., 2022) |
|  | (+)-Diasyringaresinol |  | -7.14 | (Meena et al., 2022) |
|  | Negunfurol |  | -6.92 | (Meena et al., 2022) |
|  | 4, 6-Dimethyl-11-formyl-1-oxo-4H,2,  3-dihydronaphthofuran |  | -6.73 | (Meena et al., 2022) |
|  | 4,6-Dimethyl-11-dimethoxymethyl-  1-oxo-4H,2,3-dihydronaphthofuran |  | -7.28 | (Meena et al., 2022) |
|  | 1,6-Dioxo-2(3),9(10)-  Dehydrofuranoeremophilane |  | -5.88 | (Meena et al., 2022) |
|  | Negundion F |  | -7.03 | (Meena et al., 2022) |
|  | Negundoal |  | NIL | (Meena et al., 2022) |
|  | Vitedoin B |  | -6.90 | (Meena et al., 2022) |
|  | Negundoin A |  | -7.26 | (Meena et al., 2022) |
|  | Negundoin B |  | -7.39 | (Meena et al., 2022) |
|  | Negundoin C |  | -6.97 | (Meena et al., 2022) |
|  | Negundoin G |  | -6.35 | (Meena et al., 2022) |
|  | 3β-Hydroxy-abieta-8,11,13-trien-one |  | NIL | (Meena et al., 2022) |
|  | Negundoin E |  | -7.62 | (Meena et al., 2022) |
|  | Negundol |  | -7.58 | (Meena et al., 2022) |
|  | (rel5S,6R, 8R, 9R, 10S, 13S, 16S)-  6-Acetoxy-9, 13-epoxy-16-methoxylabdan-  15, 16-olide |  | -7.29 | (Meena et al., 2022) |
|  | Negundion D |  | -7.74 | (Meena et al., 2022) |
|  | Betulinic acid |  | -8.01 | (Meena et al., 2022) |
|  | Negundonorin A |  | -8.26 | (Meena et al., 2022) |
|  | Ursolic acid |  | -7.49 | (Meena et al., 2022) |
|  | Negundonorin B |  | -8.09 | (Meena et al., 2022) |
|  | 3-epi-Corosolic acid |  | -7.45 | (Meena et al., 2022) |
|  | 3β-Acetoxyolean-12-en-27-oic acid |  | -8.36 | (Meena et al., 2022) |
|  | 2α,3α-Dihydroxyoleana-5,  12-dien-28-oic acid |  | -7.92 | (Meena et al., 2022) |
|  | 2β,3α-Diacetoxyoleana-5,  12-dien-28-oic acid |  | NIL | (Meena et al., 2022) |
|  | 2α,3β-Diacetoxy-18-hydroxyoleana-5,12-dien-28-oic acid |  | -8.83 | (Meena et al., 2022) |
|  | 3-Acetyloxy-11-en-28-oic acid prophyl ester |  | -8.64 | (Meena et al., 2022) |
|  | 3-Acetyloxy-11-oxo-olean-12-en-28-oic acid butyl ester |  | -8.64 | (Meena et al., 2022) |
|  | Oleanolic acid |  | -7.73 | (Meena et al., 2022) |
|  | 2α,3α, 23-Trihydroxyolean-12-en-28-oicacid methyl ester |  | -7.91 | (Meena et al., 2022) |
|  | 2α, 3α-Dihydroxyolean-12-en-28-oic-acid |  | -7.61 | (Meena et al., 2022) |
|  | 2α,3α,23-Trihydroxyolean-12-en-28-oic acid |  | -8.00 | (Meena et al., 2022) |
|  | Agnuside |  | -7.76 | (Meena et al., 2022) |
|  | Negundoside |  | -8.32 | (Meena et al., 2022) |
|  | 1,2-Di-substituted idopyranose |  | -8.39 | (Meena et al., 2022) |
|  | Nishindaside |  | -8.87 | (Meena et al., 2022) |
|  | 6′-p-Hydroxybenzoylmussaenosidic acid |  | -9.41 | (Meena et al., 2022) |
|  | β-Sitosterone |  | -8.16 | (Meena et al., 2022) |
|  | β-Sitosterone acetate |  | -8.79 | (Meena et al., 2022) |
|  | Stigmasterone |  | -8.45 | (Meena et al., 2022) |
|  | 24ζ -Methyl-5α-lanosta-25-one |  | -7.98 | (Meena et al., 2022) |
|  | 7-Oxositosterol |  | -7.74 | (Meena et al., 2022) |
|  | Lanostan-8, 25-dien-3β-ol |  | -7.41 | (Meena et al., 2022) |
|  | Stigmast-4-en-6β-ol-3-one |  | NIL | (Meena et al., 2022) |
|  | Ergosterol peroxide |  | -8.27 | (Meena et al., 2022) |
|  | 22,23-Dihydro-α-spinasterol-β-D-glucoside |  | -8.29 | (Meena et al., 2022) |
|  | Iso-fraxidin |  | -5.09 | (Meena et al., 2022) |
|  | Xanthotoxin |  | -5.20 | (Meena et al., 2022) |
|  | 5, 8-Dimethoxypsoralen |  | -5.66 | (Meena et al., 2022) |
|  | Tris (2,4-ditert-butylphenyl)Phosphite |  | -9.26 | (Meena et al., 2022) |
|  | n-Hentriacontanol |  | -9.58 | (Meena et al., 2022) |
|  | Salicylic acid |  | -4.35 | (Meena et al., 2022) |
|  | Betaine |  | -4.20 | (Meena et al., 2022) |
|  | Arecoline |  | -5.25 | (Meena et al., 2022) |
|  | Hippuric acid |  | -5.06 | (Meena et al., 2022) |
|  | D-(-)-Quinic acid |  | -4.78 | (Meena et al., 2022) |
|  | Nicotinic acid |  | -4.69 | (Meena et al., 2022) |
|  | Quinolinic acid |  | -4.37 | (Meena et al., 2022) |
|  | Adipic acid |  | -5.12 | (Meena et al., 2022) |
|  | Ephedrine |  | -4.67 | (Meena et al., 2022) |
|  | Vigabatrin |  | -4.14 | (Meena et al., 2022) |
|  | 4-Piperidone |  | -4.09 | (Meena et al., 2022) |
|  | 8-Hydroxyquinoline |  | -4.84 | (Meena et al., 2022) |
|  | Isovanillic acid |  | -4.88 | (Meena et al., 2022) |

**References:**

Kumar, P. P., Kumaravel, S., & Lalitha, C. (2010). African journal of biochemistry research. *African Journal of Biochemistry Research* , *4*(7), 191–195.

Meena, A. K., Perumal, A., Kumar, N., Singh, R., Ilavarasan, R., Srikanth, N., & Dhiman, K. S. (2022). Studies on physicochemical, phytochemicals, chromatographic profiling & estimation and in-silico study of Negundoside in roots & small branches of Vitex Negundo plant. *Phytomedicine Plus*, *2*(1), 100205. https://doi.org/10.1016/j.phyplu.2021.100205
