# Supplementary material for: Identification of novel amides and alkaloids as putative inhibitors of dopamine transporter for schizophrenia using computer-aided virtual screening
Source: Front Pharmacol. 2025 Apr 8;16:1509263. doi: 10.3389/fphar.2025.1509263 (PMC12039762; doi:10.3389/fphar.2025.1509263)
Supplement: Supplementary file 14 [file Table3.docx]

**Table S3.** Library of the secondary metabolites of *Thevetia peruviana* (Pers.) K. Schum.

| **Sr.**  **No.** | **Compound** | **Structure** | **Docking value**  **(Kcal/mol)** | **References** |
| --- | --- | --- | --- | --- |
|  | Kaempferol 3-O-[2""-O-sinapoyl-beta-D-glucopyranosyl]  (1$\to$4)[6"-O-sinapoyl-beta-D-glucopyranosyl](1  1$\to$2)-beta-D-galactopyranoside |  | -12.12 | (Abe et al., 1995) |
|  | Theviridoside |  | -7.51 | (Tewtrakul et al., 2002) |
|  | Theveside |  | -7.18 | (Tewtrakul et al., 2002) |
|  | pervianoside I |  | -8.70 | (Tewtrakul et al., 2002) |
|  | Peruvianoside II |  | -8.02 | (Tewtrakul et al., 2002) |
|  | Quercetin 3-*O*-[b -D-glucopyranosyl-(12)-b -D-galactopyranoside] |  | -9.25 | (Tewtrakul et al., 2002) |
|  | kaempferol 3-*O*-[b -D-glucopyranosyl-(12)-b -  D-galactopyranoside] |  | -8.69 | (Tewtrakul et al., 2002) |
|  | quercetin 3-*O*-[(6-*O*-sinapoyl)-b -  D-glucopyranosyl-(12)-b -D-galactopyranoside] |  | -11.65 | (Tewtrakul et al., 2002) |
|  | kaempferol 3-*O*-[(6-*O*-sinapoyl)-b -D-glucopyranosyl-(12)-  b -D-galactopyranoside] |  | -10.896 | (Tewtrakul et al., 2002) |
|  | quercetin 3-*O*-[(6-*O*-feruloyl)-  b -D-glucopyranosyl-(12)-b -D-galactopyranoside] |  | -9.98 | (Tewtrakul et al., 2002) |
|  | kaempferol 3-*O*-[(6-*O*-feruloyl)-b -D-glucopyranosyl-(12)-  b -D-galactopyranoside] |  | -11.46 | (Tewtrakul et al., 2002) |
|  | quercetin 3-*O*-[b -D-glucopyranosyl-(  12)-b -D-glucopyranoside] |  | -10.15 | (Tewtrakul et al., 2002) |
|  | kaempferol 3-  *O*-[b -D-glucopyranosyl-(12)-b -D-glucopyranoside] |  | -9.76 | (Tewtrakul et al., 2002) |
|  | peruvianoside III |  | -10.59 | (Tewtrakul et al., 2002) |
|  | kaempferol 3-*O*-{b -D-glucopyranosyl-(12)-[a -L-rhamonopyranosyl-(  16)]-b -D-galactopyranoside} |  | -10.55 | (Tewtrakul et al., 2002) |
|  | Cannogenol  3-O-beta-D-glucopyranosyl-  (1 6)-O-beta-D-glucopyranosyl-1 4)- alpha-L-rhamnopyranoside |  | -11.09 | (Tatsuno et al., 2021) |
|  | digitoxigenin 3-O-alpha-L-thevetopyranoside (neriifolin) |  | -8.85 | (Tatsuno et al., 2021) |
|  | thevefolin (uzarigenin 3-O-a-L-thevetopyranoside) |  | -8.84 | (Tatsuno et al., 2021) |
|  | Cerberin (digitoxigenin 3-O-(2-O-acetyl-a-L-thevetopyranoside) |  | -9.46 | (Tatsuno et al., 2021) |
|  | digitoxigenin 3-O-beta-D-glucopyranosyl-(1 4)-alpha-L-thevetopyranoside |  | -10.07 | (Tatsuno et al., 2021) |
|  | uzarigenin 3-O-beta-D-glucopyranosyl-(1 4 )-alpha-L-thevetopyranoside |  | -9.499 | (Tatsuno et al., 2021) |
|  | cannogenin 3-O-beta-D-glucopyranosyl-(1 4)-a-L-thevetopyranoside |  | -10.89 | (Tatsuno et al., 2021) |
|  | cannogenol 3-O-b-D-glucopyranosyl-(1!4)-a-L-thevetopyranoside |  | -10.51 | (Tatsuno et al., 2021) |
|  | digitoxigenin 3-O-b-D-glucopyranosyl-(1!6)-O-b-D-glucopyranosyl-a-L-acofrioside |  | -11.31 | (Tatsuno et al., 2021) |
|  | uzarigenin 3-O-b-D-glucopyranosyl-(1!6)-O-b-D-glucopyranosyl-  (1!4)-a-L-thevetopyranoside |  | -11.78 | (Tatsuno et al., 2021) |
|  | uzarigenin 3-O-b-D-glucopyranosyl-  (1!6)-O-b-D-glucopyranosyl-a-L-acofrioside |  | -10.85 | (Tatsuno et al., 2021) |
|  | cannogenol 3-O-b-D-glucopyranosyl-(1!6)-O-b-D-glucopyranosyl-(  1!4)-a-L-thevetopyranoside |  | -11.89 | (Tatsuno et al., 2021) |
|  | 19-nor-10-hydroxydigitoxigenin  3-O-b-D-glucopyranosyl-(1!6)-O-b-D-glucopyranosyl-(1!4)-a-L-thevetopyranoside |  | -11.74 | (Tatsuno et al., 2021) |
|  | (20R,S)-18,20-epoxydigitoxigenin 3-O-a-L-thevetopyranoside |  | -8.61 | (Tatsuno et al., 2021) |
|  | (20R,S)-18,20-epoxydigitoxigenin 3-O-(2-O-acetyl-a-L-thevetopyranoside) |  | Nill | (Tatsuno et al., 2021) |
|  | thevetioside A |  | -9.01 | (Tatsuno et al., 2021) |
|  | thevetioside C |  | -10.396 | (Tatsuno et al., 2021) |
|  | thevetioside F |  | -10.62 | (Tatsuno et al., 2021) |
|  | thevetin B |  | -10.78 | (Kohls et al., 2015) |
|  | thevetin A |  | -11.27 | (Kohls et al., 2015) |
|  | acetylthevetin B |  | -11.26 | (Kohls et al., 2015) |
|  | acetylthevetin A |  | -13.05 | (Kohls et al., 2015) |
|  | acetylthevetin C |  | -11.89 | (Kohls et al., 2015) |
|  | thevetin C |  | -11.15 | (Kohls et al., 2015) |
|  | Gallic acid |  | -5.16 | (El-Sawi et al., 2020) |
|  | Protocatechuic acid |  | -4.28 | (El-Sawi et al., 2020) |
|  | Chlorogenic acid |  | -7.39 | (El-Sawi et al., 2020) |
|  | Caffeic acid |  | -4.58 | (El-Sawi et al., 2020) |
|  | Vanillic acid |  | -4.57 | (El-Sawi et al., 2020) |
|  | Ferulic acid |  | -5.19 | (El-Sawi et al., 2020) |
|  | Rutin |  | -9.55 | (El-Sawi et al., 2020) |
|  | Apigenin-7-glucoside |  | -7.43 | (El-Sawi et al., 2020) |
|  | Rosmarinic acid |  | -7.27 | (El-Sawi et al., 2020) |
|  | Cinnamic acid |  | -4.83 | (El-Sawi et al., 2020) |
|  | Decane |  | -5.24 | (El-Sawi et al., 2020) |
|  | 4-Methyldecane |  | -5.32 | (El-Sawi et al., 2020) |
|  | 2-Methyldecane |  | -5.28 | (El-Sawi et al., 2020) |
|  | 3-Methyldecane |  | -5.25 | (El-Sawi et al., 2020) |
|  | 1-Methyl-4-(1-methylethyl)benzene |  | -4.71 | (El-Sawi et al., 2020) |
|  | Pentylcyclohexane |  | -4.83 | (El-Sawi et al., 2020) |
|  | Tetramethyl benzene |  | -4.77 | (El-Sawi et al., 2020) |
|  | 2-Methylundecane |  | -5.50 | (El-Sawi et al., 2020) |
|  | 3-Methylundecane |  | -5.48 | (El-Sawi et al., 2020) |
|  | Dodecane |  | -5.48 | (El-Sawi et al., 2020) |
|  | 2,6-Dimethylundecane |  | -5.57 | (El-Sawi et al., 2020) |
|  | 2-Tetradecene |  | -6.57 | (El-Sawi et al., 2020) |
|  | 2,6-Di(t-butyl)-4-hydroxy-4-methyl-2,5-  cyclohexadiene-1-one |  | -5.84 | (El-Sawi et al., 2020) |
|  | Butylated hydroxytoulene |  | -6.30 | (El-Sawi et al., 2020) |
|  | 1-Hexadecene |  | -6.54 | (El-Sawi et al., 2020) |
|  | 1-Butylheptyl- benzene |  | -7.49 | (El-Sawi et al., 2020) |
|  | 1-Pentylheptyl- benzene |  | -7.02 | (El-Sawi et al., 2020) |
|  | 1-Propylnonyl- benzene |  | -7.15 | (El-Sawi et al., 2020) |
|  | Trimethyl, 2-pentadecanone |  | -7.25 | (El-Sawi et al., 2020) |
|  | Benzenedicarboxylic acid dibutyl ester |  | -7.46 | (El-Sawi et al., 2020) |
|  | 3-Eicosene |  | -7.57 | (El-Sawi et al., 2020) |
|  | 5-Eicosene |  | -7.72 | (El-Sawi et al., 2020) |
|  | Tricosane |  | -8.49 | (El-Sawi et al., 2020) |
|  | Tetramethyl-heptadecane |  | -7.49 | (El-Sawi et al., 2020) |
|  | 9-Tricosene |  | -8.44 | (El-Sawi et al., 2020) |
|  | Cyclotetracosane |  | -7.74 | (El-Sawi et al., 2020) |
|  | Pentacosane |  | -9.03 | (El-Sawi et al., 2020) |
|  | Hexacosane |  | -9.96 | (El-Sawi et al., 2020) |
|  | Octacosane |  | -9.77 | (El-Sawi et al., 2020) |
|  | Nonacosane |  | -9.63 | (El-Sawi et al., 2020) |
|  | Triacontane |  | -9.15 | (El-Sawi et al., 2020) |
|  | Hentriacontane |  | -10.47 | (El-Sawi et al., 2020) |
|  | Cholestrol |  | -8.28 | (El-Sawi et al., 2020) |
|  | Tritriacontane |  | -9.86 | (El-Sawi et al., 2020) |
|  | Gamma-Sitosterol |  | -8.48 | (El-Sawi et al., 2020) |
|  | Pentatriacontane |  | -9.83 | (El-Sawi et al., 2020) |
|  | Heptatriacontane |  | -11.05 | (El-Sawi et al., 2020) |
|  | Dodecanoic acid methyl ester |  | -7.46 | (El-Sawi et al., 2020) |
|  | Tetradecanoic acid methyl ester |  | -7.01 | (El-Sawi et al., 2020) |
|  | Pentadecenoic acid methyl ester |  | -7.13 | (El-Sawi et al., 2020) |
|  | Pentadecanoic acid methyl ester |  | -7.64 | (El-Sawi et al., 2020) |
|  | 9-Hexadecenoic acid methyl ester |  | -7.11 | (El-Sawi et al., 2020) |
|  | Heptadecanoic acid methyl ester |  | -7.68 | (El-Sawi et al., 2020) |
|  | Hexadecanoic acid 3-hydroxy  methyl ester |  | -7.86 | (El-Sawi et al., 2020) |
|  | Nonadecanoic acid methyl ester |  | -8.04 | (El-Sawi et al., 2020) |
|  | Eicosenoic acid methyl ester |  | -8.56 | (El-Sawi et al., 2020) |
|  | Eicosanoic acid methyl ester |  | -8.28 | (El-Sawi et al., 2020) |
|  | Tetramethylheptadecan-4-olide |  | -7.83 | (El-Sawi et al., 2020) |
|  | Heneicosanoic acid methyl ester |  | -8.67 | (El-Sawi et al., 2020) |
|  | Docosanoic acid methyl ester |  | -8.65 | (El-Sawi et al., 2020) |
|  | Tricosanoic acid methyl ester |  | -8.56 | (El-Sawi et al., 2020) |
|  | Tetracosanoic acid methyl ester |  | -9.50 | (El-Sawi et al., 2020) |
|  | Pentacosanoic acid methyl ester |  | -8.49 | (El-Sawi et al., 2020) |
|  | Hexacosanoic acid methyl ester |  | -9.59 | (El-Sawi et al., 2020) |
|  | Heptacosanoic acid methyl ester |  | -9.16 | (El-Sawi et al., 2020) |
|  | Octacosanoic acid methyl ester |  | -9.51 | (El-Sawi et al., 2020) |
|  | Nonacosanoic acid methyl ester |  | -9.83 | (El-Sawi et al., 2020) |
|  | Triacontanoic acid methyl ester |  | -9.74 | (El-Sawi et al., 2020) |

**References:**

Abe, F., Iwase, Y., Yamauchi, T., Yahara, S., & Nohara, T. (1995). Flavonol sinapoyl glycosides from leaves of Thevetia peruviana. *Phytochemistry*, *40*(2), 577–581. https://doi.org/10.1016/0031-9422(95)00316-Y

El-Sawi, S. A., Maamoun, A. A., Salama, A. H., & Farghaly, A. A. (2020). Chemical profiling of Thevetia peruviana leaves cytotoxic active extracts enhanced by microemulsion formulation. *Bulletin of the National Research Centre*, *44*(1). https://doi.org/10.1186/s42269-020-00339-3

Kohls, S., Scholz-Böttcher, B. M., Teske, J., & Rullkötter, J. (2015). Isolation and quantification of six cardiac glycosides from the seeds of Thevetia peruviana provide a basis for toxological survey. *Indian Journal of Chemistry - Section B Organic and Medicinal Chemistry*, *54B*(12), 1502–1510.

Tatsuno, S., Iguchi, T., Kuroda, M., Ishihara, M., Sakagami, H., & Mimaki, Y. (2021). A new and 23 known cardenolide glycosides from Thevetia neriifolia seeds and their cytotoxic activities against human oral carcinoma cell lines. *Natural Product Research*, *35*(22), 4388–4393. https://doi.org/10.1080/14786419.2020.1716352

Tewtrakul, S., Nakamura, N., Hattori, M., Fujiwara, T., & Supavita, T. (2002). Flavanone and flavonol glycosides from the leaves of Thevetia peruviana and their HIV-1 reverse transcriptase and HIV-1 integrase inhibitory activities. *Chemical and Pharmaceutical Bulletin*, *50*(5), 630–635. https://doi.org/10.1248/cpb.50.630
