# Supplementary material for: Identification of novel amides and alkaloids as putative inhibitors of dopamine transporter for schizophrenia using computer-aided virtual screening
Source: Front Pharmacol. 2025 Apr 8;16:1509263. doi: 10.3389/fphar.2025.1509263 (PMC12039762; doi:10.3389/fphar.2025.1509263)
Supplement: Supplementary file 15 [file Table4.docx]

**Table S4. Library of the secondary metabolites of *Ficus hirta vahl.***

| **Sr.**  **No.** | **Compounds** | **Structure** | **Docking value**  **(Kcal/mol)** | **References** |
| --- | --- | --- | --- | --- |
|  | Psoralen |  | -5.45 | (Lansky et al., 2008) |
|  | Apigenin |  | -5.88 | (Lansky et al., 2008) |
|  | Hesperidin |  | -9.17 | (Lansky et al., 2008) |
|  | 5-Hydroxy-4,6,7,8-tetramethoxyflavone |  | -6.82 | (Lansky et al., 2008) |
|  | 4,5,6,7,8-Pentamethoxyflavone |  | -6.93 | (Lansky et al., 2008) |
|  | 3beta-Acetoxy-alpha-amyrin |  | -8.56 | (Lansky et al., 2008) |
|  | 3beta-Acetoxy-beta-amyrin |  | Nil | (Lansky et al., 2008) |
|  | 3beta-Hydroxy-stigmast-5-en-7-one |  | -7.99 | (Lansky et al., 2008) |
|  | (2R) methyl 2-O-β-Dglucopyranosyl-  2-phenyl acetate |  | -6.72 | (Wan et al., 2016) |
|  | (2S) 2-O-benzoyl-butanedioic  acid-4-methyl ester. |  | -5.56 | (Wan et al., 2016) |
|  | 4-O-benzoyl-quinic acid. |  | -6.66 | (Wan et al., 2016) |
|  | 4-O-benzoyl-quinic acid methyl ester |  | -6.57 | (Wan et al., 2016) |
|  | 3-O-benzoyl-quinic acid |  | -6.910 | (Wan et al., 2016) |
|  | 2-phenylethyl-O-β-D-glucoside |  | -6.65 | (Wan et al., 2016) |
|  | 2-phenylethyl-O-β-vicianoside |  | -8.18 | (Wan et al., 2016) |
|  | benzyl-β-D-glucopyranoside |  | -6.88 | (Wan et al., 2016) |
|  | 1-O-transcinnamoyl-β-D-glucopyranosyl-(1→6)-β-D-glucopyranoside |  | -8.19 | (Wan et al., 2016) |
|  | (1′S)-methoxy-4-(1-propionyloxy-5-methoxycarboxyl-pentyloxy)-(E)-formylvinyl |  | -7.86 | (Cheng, Yi, Chen, et al., 2017) |
|  | (8R)-4,5′-dihydroxy-8-hydroxymehtyl-3′-methoxydeoxybenzoin |  | -7.01 | (Cheng, Yi, Chen, et al., 2017) |
|  | (2′S)-3-[2,3-dihydro-6-hydroxy-2-(1-hydroxy-1-methylethyl)-5-benzofuranyl] methyl propionate |  | -6.55 | (Cheng, Yi, Chen, et al., 2017) |
|  | 3-[6-(5-O-β-D-glucopyranosyl)benzofuranyl] methyl propionate |  | -8.72 | (Cheng, Yi, Chen, et al., 2017) |
|  | Methylcnidioside A |  | Nil | (Cheng, Yi, Chen, et al., 2017) |
|  | (E)-3-[5-(6-hydroxy) benzofuranyl] propenoic acid |  | -6.17 | (Cheng, Yi, Chen, et al., 2017) |
|  | Ficuscarpanoside A |  | -7.73 | (Cheng, Yi, Chen, et al., 2017) |
|  | syringaresinol |  | -8.55 | (Cheng, Yi, Chen, et al., 2017) |
|  | (7R,8S)-ficusal |  | -7.60 | (Cheng, Yi, Chen, et al., 2017) |
|  | trans-p-hydroxycinnamic acid |  | -5.03 | (Cheng, Yi, Chen, et al., 2017) |
|  | 1′-O-β-D-glucopyranosyl(2R,3S)-3-hydroxynodakenetin |  | -7.66 | (Cheng, Yi, Chen, et al., 2017) |
|  | p-hydroxybenzoic acid |  | -4.67 | (Cheng, Yi, Chen, et al., 2017) |
|  | syringic acid |  | -5.45 | (Cheng, Yi, Chen, et al., 2017) |
|  | ficuglucoside |  | -6.72 | (Cheng, Yi, Chen, et al., 2017) |
|  | 1-methyl-1,2,3,4-tetrahydro-β-carboline-3-carboxylic acid |  | -5.80 | (Wan et al., 2017) |
|  | methyl 1-methyl-1,2,3,4-tetrahydro-β-carboline-3-carboxylate |  | -6.38 | (Wan et al., 2017) |
|  | vomifoliol |  | -5.62 | (Wan et al., 2017) |
|  | dehydrovomifoliol |  | -5.84 | (Wan et al., 2017) |
|  | Icariside B2 |  | -7.71 | (Wan et al., 2017) |
|  | dihydrophaseic acid |  | -6.14 | (Wan et al., 2017) |
|  | Pubinernoid A |  | -5.15 | (Wan et al., 2017) |
|  | Pinocembrin-7-O-β-D-glucoside |  | -7.83 | (Wan et al., 2017) |
|  | Naringenin-7-O-β-D-glucoside |  | -8.58 | (Wan et al., 2017) |
|  | Eriodictyol-7-O-β-D-glucoside |  | -7.89 | (Wan et al., 2017) |
|  | 1-phenylpropane-1,2-diol |  | -4.86 | (Wan et al., 2017) |
|  | Umbelliferon |  | -5.17 | (Cheng, Yi, Wang, et al., 2017) |
|  | 7-(20,30-dihydroxy-30-methylbutoxy)-coumarin |  | -6.35 | (Cheng, Yi, Wang, et al., 2017) |
|  | Nodakenetin |  | -6.31 | (Cheng, Yi, Wang, et al., 2017) |
|  | (1R, 2R, 5R, 6S)-6-(4-hydroxy-3, 5-dimethoxyphenyl)-3, 7-  dioxabicyclo[3, 3, 0] octan-2-ol |  | -6.84 | (Cheng, Yi, Wang, et al., 2017) |
|  | (+)-(7R, 8R)-4-hydroxy-3,30,50-trimethoxy-80,90-dinor-8,40-o  xyneoligna-7,9-diol-70-aldehyde |  | -7.48 | (Cheng, Yi, Wang, et al., 2017) |
|  | (_)-(7S,8R)-4-hydroxy-3,30,50-trimethoxy-80,90-dinor-8,40-oxyneo  ligna -7,9-diol-70-aldehyde |  | -7.38 | (Cheng, Yi, Wang, et al., 2017) |
|  | (1R, 2R, 5R, 6S)-6-(4-  hydroxy-3-methoxyphenyl)-3,7-dioxabicyclo [3,3,0] octan-2-ol |  | -6.41 | (Cheng, Yi, Wang, et al., 2017) |
|  | (_)-pinoresinol |  | -7.75 | (Cheng, Yi, Wang, et al., 2017) |
|  | 2-[4-(3-hydroxy  propyl)-2-methoxyphenoxy] propane-1, 3-diol |  | -6.098 | (Cheng, Yi, Wang, et al., 2017) |
|  | Vanillin |  | -4.11 | (Cheng, Yi, Wang, et al., 2017) |
|  | 3-hydroxy-4-methoxy-trans- cinnamaldehyde |  | -4.85 | (Cheng, Yi, Wang, et al., 2017) |
|  | vanillin acid |  | -4.38 | (Cheng, Yi, Wang, et al., 2017) |
|  | Beta-hydroxypropiovanillone |  | -4.999 | (Cheng, Yi, Wang, et al., 2017) |
|  | 7-O-ethylguaiacylglycerol |  | -6.01 | (Cheng, Yi, Wang, et al., 2017) |
|  | Evofolin-B |  | -6.59 | (Cheng, Yi, Wang, et al., 2017) |
|  | (E)-3-[5-(6-methoxy) benzofuranyl]  propenoic acid |  | -5.25 | (Cheng, Yi, Wang, et al., 2017) |
|  | (E)-isopsoralic acid 1 6-O-beta-D-glucopyranoside |  | -7.39 | (Cheng, Yi, Wang, et al., 2017) |
|  | (Z)-isopsoralic acid 1 6-O-beta-D glucopyranoside |  | -6.97 | (Cheng, Yi, Wang, et al., 2017) |
|  | Phenyl beta-D-glucopyranoside |  | -5.72 | (Cheng, Yi, Wang, et al., 2017) |
|  | 2,3-dihydroxy-1-(4-hydroxy-3-methoxyphenyl)-  propan-1-one |  | -5.42 | (Cheng, Yi, Wang, et al., 2017) |
|  | 3,4,5-trimethoxybenzyl beta-D-glucopyranoside |  | -7.11 | (Cheng, Yi, Wang, et al., 2017) |
|  | 3,4-dimethoxyphenyl-1-O-beta-D-glucopyranoside |  | -6.22 | (Cheng, Yi, Wang, et al., 2017) |
|  | 3,4,5-trimethoxy phenoltetraacetyl-beta-D- glucopyranoside |  | -6.87 | (Cheng, Yi, Wang, et al., 2017) |
|  | 1,3,5-trimethoxybenzene |  | -4.90 | (Cheng, Yi, Wang, et al., 2017) |
|  | (Z)-3-[5-(6-O-b-D-glucopyranosyl) benzofuranyl] methyl propenoate |  | -7.29 | (Cheng, Yi, Wang, et al., 2017) |
|  | (Z)-3-[5-(6-methoxy) benzofuranyl] propenoic acid |  | -5.36 | (Cheng, Yi, Wang, et al., 2017) |
|  | (10S)-6-(20-hydroxy-10-O-beta-D-glucopyranoside)-7-hydroxycoumarin |  | -6.96 | (Cheng, Yi, Wang, et al., 2017) |
|  | (2S)-1-O-beta-D-glucopyranosyl-2-O-(2-methoxy-4-phenylaldehyde) propane-3-ol |  | -7.57 | (Cheng, Yi, Wang, et al., 2017) |
|  | Ficuside A |  | -9.21 | (Ye et al., 2020) |
|  | Ficuside B |  | -8.95 | (Ye et al., 2020) |
|  | Ficuside C |  | Nil | (Ye et al., 2020) |
|  | Ficuside D |  | Nil | (Ye et al., 2020) |
|  | Ficuside E |  | Nil | (Ye et al., 2020) |
|  | Ficuside F |  | -8.68 | (Ye et al., 2020) |
|  | Ficuside G |  | -8.896 | (Ye et al., 2020) |
|  | 3,4-dimethoxyphenyl-1-O-β-D-apiofuranosyl-(1→ 2)-β-D-glucopyranoside |  | -9.06 | (Ye et al., 2020) |
|  | khaephuoside A |  | -9.34 | (Ye et al., 2020) |
|  | 2-methoxyphenol-O-β-D-apiofuranosyl-(1→2)-β-D-glucopyranoside |  | Nil | (Ye et al., 2020) |
|  | methyl 2-hydroxybenzoate-2-O-β-D-apiofuranosyl-(1→ 2)-O-β-D-glucopyranoside |  | Nil | (Ye et al., 2020) |
|  | markhamioside F |  | -8.64 | (Ye et al., 2020) |
|  | benzyl-O-β-D-apiofuranosyl-(1→2)-β-D glucopyranoside |  | -7.69 | (Ye et al., 2020) |
|  | 3,4,5-trimethoxyphenyl-1-O-β-apiofuranosyl-(1″→6′)-β-glucopyranoside |  | -8.76 | (Ye et al., 2020) |
|  | di-O-methylcrenatin |  | -7.16 | (Ye et al., 2020) |
|  | 3,4-dimethoxyphenyl-β-D-glucopyranoside |  | -6.95 | (Ye et al., 2020) |
|  | phenyl β-D-glucopyranoside |  | -6.51 | (Ye et al., 2020) |
|  | 2,4,6-trimethoxy-1-O-β-D-glycoside |  | -7.18 | (Ye et al., 2020) |
|  | 2,6-dimethoxy-4-hydroxyphenol-1-O-β-D-glucopyranoside |  | -7.63 | (Ye et al., 2020) |
|  | methyl 2-O-β-D-glucopyranosylbenzoate |  | -7.34 | (Ye et al., 2020) |
|  | glucosyringic acid |  | -7.37 | (Ye et al., 2020) |
|  | 4-(β-D-glucopyranosyloxy)benzoic acid |  | -6.69 | (Ye et al., 2020) |
|  | vanillic acid 4-O-β-D-glucopyranoside |  | -6.79 | (Ye et al., 2020) |
|  | (1′R)-1′-(4-hydroxy-3,5-dimethoxyphenyl)propan-1′-ol-4-O-β-D-glucopyranoside |  | -8.14 | (Ye et al., 2020) |
|  | nikoenoside |  | -7.85 | (Ye et al., 2020) |
|  | 4-(3′-hydroxypropyl)-2,6-dimethoxyphnol-3′-O-β-D-glcoside |  | -7.96 | (Ye et al., 2020) |
|  | gentisic acid 5-O-β-D-xylo-side |  | -6.87 | (Ye et al., 2020) |
|  | luteolin |  | -6.22 | (Cruz, Corrêa, Lamarão, Kinupp, et al., 2022) |
|  | Arbutin |  | -6.71 | (Cruz, Corrêa, Lamarão, Sanches, et al., 2022) |

**References:**

Cheng, J., Yi, X., Chen, H., Wang, Y., & He, X. (2017). Anti-inflammatory phenylpropanoids and phenolics from Ficus hirta Vahl. *Fitoterapia*, *121*(August), 229–234. https://doi.org/10.1016/j.fitote.2017.07.018

Cheng, J., Yi, X., Wang, Y., Huang, X., & He, X. (2017). Phenolics from the roots of hairy fig (Ficus hirta Vahl.) exert prominent anti-inflammatory activity. *Journal of Functional Foods*, *31*, 79–88. https://doi.org/10.1016/j.jff.2017.01.035

Cruz, J. M. dos A., Corrêa, R. F., Lamarão, C. V., Kinupp, V. F., Sanches, E. A., Campelo, P. H., & Bezerra, J. de A. (2022). Ficus spp. fruits: Bioactive compounds and chemical, biological and pharmacological properties. *Food Research International*, *152*(December 2021). https://doi.org/10.1016/j.foodres.2021.110928

Cruz, J. M. dos A., Corrêa, R. F., Lamarão, C. V., Sanches, E. A., Campelo, P. H., & Bezerra, J. de A. (2022). Ficus spp.: Phytochemical composition and medicinal potential. *Research, Society and Development*, *11*(12), e265111234135. https://doi.org/10.33448/rsd-v11i12.34135

Lansky, E. P., Paavilainen, H. M., Pawlus, A. D., & Newman, R. A. (2008). Ficus spp. (fig): Ethnobotany and potential as anticancer and anti-inflammatory agents. *Journal of Ethnopharmacology*, *119*(2), 195–213. https://doi.org/10.1016/j.jep.2008.06.025

Wan, C., Chen, C., Li, M., Yang, Y., Chen, M., & Chen, J. (2017). Chemical constituents and antifungal activity of Ficus hirta vahl. fruits. *Plants*, *6*(4), 1–9. https://doi.org/10.3390/plants6040044

Wan, C., Han, J., Chen, C., Yao, L., Chen, J., & Yuan, T. (2016). Monosubstituted Benzene Derivatives from Fruits of Ficus hirta and Their Antifungal Activity against Phytopathogen Penicillium italicum. *Journal of Agricultural and Food Chemistry*, *64*(28), 5621–5624. https://doi.org/10.1021/acs.jafc.6b02176

Ye, X., Tian, W., Wang, G., Zhang, X., Zhou, M., Zeng, D., Liu, X., Yao, X., Zhang, Y., & Chen, H. (2020). Phenolic Glycosides from the Roots of Ficus hirta Vahl. And Their Antineuroinflammatory Activities. *Journal of Agricultural and Food Chemistry*, *68*(14), 4196–4204. https://doi.org/10.1021/acs.jafc.9b07876
