# Supplementary material for: Identification of novel amides and alkaloids as putative inhibitors of dopamine transporter for schizophrenia using computer-aided virtual screening
Source: Front Pharmacol. 2025 Apr 8;16:1509263. doi: 10.3389/fphar.2025.1509263 (PMC12039762; doi:10.3389/fphar.2025.1509263)
Supplement: Supplementary file 16 [file Table6.docx]

**Table S6.** Library of the secondary metabolites of *Datura metel* L.

| **Sr.**  **No.** | **Compound** | **Structure** | **Docking value**  **(Kcal/mol)** | **References** |
| --- | --- | --- | --- | --- |
|  | 1-Pentadecanol |  | -7.66 | (Jabeen et al., 2022) |
|  | 1-Heptadecene |  | -6.98 | (Jabeen et al., 2022) |
|  | 1-Octadecanol |  | -7.67 | (Jabeen et al., 2022) |
|  | 1,2-Benzenedicarboxylic  acid, bis (2-methylpropyl)  ester |  | -7.19 | (Jabeen et al., 2022) |
|  | 1-Pentadecene |  | -7.04 | (Jabeen et al., 2022) |
|  | 1-Tetradecanol |  | -6.52 | (Jabeen et al., 2022) |
|  | 1,3(15),10-Bisabolatriene |  | -6.96 | (Jabeen et al., 2022) |
|  | 1-Eicosanol |  | -7.96 | (Jabeen et al., 2022) |
|  | Myristic acid, methyl ester |  | -7.02 | (Cholilalah, Rois Arifin, 1967) |
|  | 7,10,13-Hexadecatrienoic  acid, methyl ester |  | -7.62 | (Cholilalah, Rois Arifin, 1967) |
|  | Linoleic acid, methyl ester |  | -8.15 | (Cholilalah, Rois Arifin, 1967) |
|  | Linolenic acid, methyl ester |  | -8.05 | (Cholilalah, Rois Arifin, 1967) |
|  | Stearic acid, methyl ester |  | -8.44 | (Cholilalah, Rois Arifin, 1967) |
|  | Arachidic acid methyl ester |  | -8.48 | (Cholilalah, Rois Arifin, 1967) |
|  | Behenic acid, methyl ester |  | -8.97 | (Cholilalah, Rois Arifin, 1967) |
|  | Hexatriacontane |  | -9.51 | (Cholilalah, Rois Arifin, 1967) |
|  | Octadecanoic acid, 17-  methyl-, methyl ester |  | -8.72 | (Cholilalah, Rois Arifin, 1967) |
|  | 10,13-Eicosadienoic acid,  methyl ester |  | -8.24 | (Cholilalah, Rois Arifin, 1967) |
|  | Tetratriacontane |  | -9.94 | (Cholilalah, Rois Arifin, 1967) |
|  | Fumaric acid |  | -4.46 | (Cholilalah, Rois Arifin, 1967) |
|  | Naringin |  | -9.16 | (Cholilalah, Rois Arifin, 1967) |
|  | Diosmin |  | -10.32 | (Cholilalah, Rois Arifin, 1967) |
|  | Gentisic acid |  | -4.68 | (Cholilalah, Rois Arifin, 1967) |
|  | 16ß,17-dihydroxy-ent-kauran-19-oic acid, |  | Nil | (Cholilalah, Rois Arifin, 1967) |
|  | paniculoside-IV |  | -8.19 | (Cholilalah, Rois Arifin, 1967) |
|  | Daturmetelide A |  | -8.15 | (Tan et al., 2020) |
|  | Daturmetelide B |  | -8.52 | (Tan et al., 2020) |
|  | Daturmetelide C |  | -8.17 | (Tan et al., 2020) |
|  | Daturmetelide D |  | -8.07 | (Tan et al., 2020) |
|  | Daturmetelide E |  | Nil | (Tan et al., 2020) |
|  | Daturmetelide F |  | -8.17 | (Tan et al., 2020) |
|  | Daturmetelide G |  | -8.62 | (Tan et al., 2020) |
|  | Daturmetelide H |  | -8.39 | (Tan et al., 2020) |
|  | Daturmetelide I |  | -8.47 | (Tan et al., 2020) |
|  | Daturmetelide J |  | Nil | (Tan et al., 2020) |
|  | Daturmetelide K |  | Nil | (Tan et al., 2020) |
|  | Daturmetelide L |  | -8.26 | (Tan et al., 2020) |
|  | Daturmetelide M |  | Nil | (Tan et al., 2020) |
|  | Daturmetelide N |  | -9.87 | (Tan et al., 2020) |
|  | Daturmetelide O |  | -9.60 | (Tan et al., 2020) |
|  | Daturmetelide P |  | -8.88 | (Tan et al., 2020) |
|  | Daturmetelide Q |  | Nil | (Tan et al., 2020) |
|  | Daturmetelide R |  | -9.21 | (Tan et al., 2020) |
|  | Daturmetelide S |  | -9.28 | (Tan et al., 2020) |
|  | Daturmetelide T |  | -9.36 | (Tan et al., 2020) |
|  | Daturmetelide U |  | -9.61 | (Tan et al., 2020) |
|  | Daturmetelide V |  | -9.79 | (Tan et al., 2020) |
|  | Daturmetelide W |  | -9.67 | (Tan et al., 2020) |
|  | *Pregnane A* |  | -8.23 | (Liu et al., 2020) |
|  | *Daturafoliside X* |  | -11.18 | (Liu et al., 2020) |
|  | *Daturafoliside Y* |  | -9.79 | (Liu et al., 2020) |
|  | daturafoliside E |  | -10.32 | (Liu et al., 2020) |
|  | Daturataurin B |  | -10.14 | (Liu et al., 2020) |
|  | baimantuluodine K |  | -8.61 | (Liu et al., 2020) |
|  | 1β,5α,7β-guaiane-4β,10α,11-triol |  | -6.66 | (Mai et al., 2017) |
|  | 1α,5α,7α-11- guaiene-2α,3β,4α,10α,13-pentaol |  | -6.40 | (Mai et al., 2017) |
|  | pterodontriol B |  | -6.34 | (Mai et al., 2017) |
|  | Disciferitrio |  | -6.21 | (Mai et al., 2017) |
|  | 7β,16α,17-trihydroxy-ent-kauran-19-oic acid 19-O-β-D-glucopyranoside  Ester |  | Nil | (Yang et al., 2018) |
|  | 16α,17-dihydroxy-ent-kauran-19-oic acid 19-O-β-Dglucopyranosy-(  6′ → 1′′′) -β-D-glucopyranosy-(2′ → 1′′) -β-Dglucopyranoside |  | -9.11 | (Yang et al., 2018) |
|  | Daturaterpenoid a |  | -6.09 | (Liu et al., 2019) |
|  | Daturaterpenoid B |  | -6.22 | (Liu et al., 2019) |
|  | Daturaterpenoid C |  | -6.42 | (Liu et al., 2019) |
|  | Daturaterpenoid D |  | -7.95 | (Liu et al., 2019) |
|  | (6R,9R)-3-oxo-α-ionol-9-O-β-D-glucopyranoside |  | -8.12 | (Liu et al., 2019) |
|  | Solavetivone-14-O-β-D-glucopyranoside |  | -7.82 | (Liu et al., 2019) |
|  | 5/, 7 *l*, dimethyl 6 *l-*hydroxyl 3*l* phenyl 3-alpha-amine beta-yne sitosterol |  | -9.92 | (Okwu & Igara, 2009) |
|  | 2-Propanone, 1-[4-(1-methyl-2- pyrrolidinyl)-1-methyl-2-pyrrolidinyl]- |  | -6.61 | (Lawal et al., 2023) |
|  | 9,12,15-Octadecatrienoic acid, methyl ester |  | -7.54 | (Lawal et al., 2023) |
|  | 8-Azabicyclo [3.2.1]octan-3-ol |  | -3.99 | (Lawal et al., 2023) |
|  | Isophytol |  | -7.84 | (Lawal et al., 2023) |
|  | l-(+)-Ascorbic acid 2,6-dihexadecanoate |  | -10.29 | (Lawal et al., 2023) |
|  | 2-Butenoic acid, 2-methyl-,8-methyl-6- (1-oxopropoxy)-8-azabicyclo [3.2.1]oct- 3-yl ester, [1R-[1.alpha.,3.beta.(E),5. alpha.,6.alpha.]]- |  | -7.45 | (Lawal et al., 2023) |
|  | Citronellyl isobutyrate |  | -6.89 | (Lawal et al., 2023) |
|  | Phytol acetate |  | -8.02 | (Lawal et al., 2023) |
|  | Atropine |  | Nil | (Lawal et al., 2023) |
|  | 8-Azabicyclo [3.2.1]octane-3,6-diol, diacetate (ester) |  | -5.78 | (Lawal et al., 2023) |
|  | Glycerol 1-palmitate |  | -9.02 | (Lawal et al., 2023) |
|  | Butyl 9,12,15-octadecatrienoate |  | -8.08 | (Lawal et al., 2023) |
|  | Bacteriochlorophyll-c-stearyl |  | Nil | (Lawal et al., 2023) |
|  | 3-(Methoxymethoxy)cholest-4-ene |  | -8.33 | (Lawal et al., 2023) |
|  | Campesterol |  | -7.68 | (Lawal et al., 2023) |
|  | (-)-Loliolide |  | -5.17 | (Prasathkumar et al., 2022) |
|  | 9,12,15-octadecatrienoic acid, (Z,Z,Z)- |  | -8.09 | (Prasathkumar et al., 2022) |
|  | Heptasiloxane, hexadecamethyl- |  | -10.51 | (Prasathkumar et al., 2022) |
|  | 13-docosenamide, (Z)- |  | -7.70 | (Prasathkumar et al., 2022) |
|  | Daturabietatriene |  | -6.53 | (SHUAIB, 1996) |
|  | Daturasterol |  | -7.62 | (SHUAIB, 1996) |
|  | Daturametelin C |  | -8.88 | (Takeshi, 1977) |
|  | Daturametelin D |  | -8.51 | (Takeshi, 1977) |
|  | Daturametelin G-Ac |  | -9.89 | (Takeshi, 1977) |
|  | Daturametelin F |  | -8.58 | (Takeshi, 1977) |
|  | Daturametelin E |  | -9.19 | (Takeshi, 1977) |
|  | Daturaolone (3-oxo-6*β*-hydroxy-*β*-amyrin) |  | -7.77 | (Bawazeer et al., 2020) |
|  | Adenosine,4ꞌde (hydroxymethyl)-4ꞌ-(N-ethylaminoformyl |  | -8.35 | (S.C. et al., 2022) |
|  | Chlorozotocin |  | -7.15 | (S.C. et al., 2022) |
|  | 2-methyl-3-thiosemicarbazide |  | -4.10 | (S.C. et al., 2022) |
|  | Hexestrol,pentafluoropropionyl- |  | -7.43 | (S.C. et al., 2022) |
|  | (1,2-Diethylethylene)bis(phenylene) diacetate |  | -7.10 | (S.C. et al., 2022) |
|  | 3-Methylbenzylalcohol,trifluoroacetate |  | -5.57 | (S.C. et al., 2022) |
|  | 4-Methylbenzyl alcohol, trifluoroacetate |  | -5.74 | (S.C. et al., 2022) |
|  | Trisiloxane ,1,1,1,5,5,5-hexamethyl-3-〔(trimethylsily)oxy〕 |  | -8.06 | (S.C. et al., 2022) |
|  | Cyclotetrasiloxane,octamethyl- |  | -7.13 | (S.C. et al., 2022) |
|  | Estragole |  | -5.27 | (S.C. et al., 2022) |
|  | Anethole |  | -5.56 | (S.C. et al., 2022) |
|  | (-)-Epigallocatechin |  | -6.58 | (S.C. et al., 2022) |
|  | Catechin |  | -6.48 | (S.C. et al., 2022) |
|  | Ellagic acid |  | -6.48 | (S.C. et al., 2022) |
|  | 1,1ꞌ-Biphenyl〕-3,3ꞌ-dicarboxaldehyde,6,6ꞌ-dihydroxy-5,5ꞌ-dimethoxyl |  | -7.37 | (S.C. et al., 2022) |
|  | 3-Pyridinecarboxylicacid,2,7,10-tris(acetyloxy)-1,1a,2,3,4,6,7,10,11,11a-decahydro-1,1,3,6,9-pentamethyl-4-oxo-4a,7a-epoxy-5H-cyclopenta〔a〕cyclopropa〔f〕cycloundecen-11-yl ester,〔1Ar-1aR*,2R*,3S*,4aR*,6S*,7S*,7aS*,8E,10R*,11R*,11As*) |  | -9.88 | (S.C. et al., 2022) |
|  | Hexadecanoic 1a,2,5,5a,6,9,10,10a-octahydro-5,5a-dihydroxy-4-(hydroxymethyl)-1,1,7,9-tetramethyl-11-oxo-1H-2,8a-methanocyclopenta〔a〕cyclopropa〔e〕cyclodecen-6-yl ester, 〔1aR-(1a,9a,10a)〕 |  | -9.83 | (S.C. et al., 2022) |
|  | Lorazepam |  | -7.25 | (S.C. et al., 2022) |
|  | Quinazoline-2-carboxaldehyde,6-chloro-4-(2-chlorophenyl)- |  | -6.97 | (S.C. et al., 2022) |
|  | 2,4-Imidazolidinedione,5-[3,4-bis[(trimethylsily)oxy]phenyl]-3-methyl-5-phenyl-1-(trimethylsilyl |  | -10.04 | (S.C. et al., 2022) |
|  | Benzofuran-2-Carboxylic acid |  | -4.96 | (S.C. et al., 2022) |
|  | Benzofuran-5-carboxylic acid |  | -4.95 | (S.C. et al., 2022) |
|  | PGF2ᾰ |  | -8.17 | (S.C. et al., 2022) |
|  | ᾰ-Tocopheryl acetate |  | -10.11 | (S.C. et al., 2022) |
|  | hexadecanoicacid,3,7,11,15-tetramethyl-2-hexadecenylester,〔R-〔R,R-E〕 |  | -11.16 | (S.C. et al., 2022) |
|  | Ethyl iso-alocholate |  | -8.67 | (S.C. et al., 2022) |
|  | 1-Heptatriacotanol |  | -10.26 | (S.C. et al., 2022) |
|  | Linoelaidic acid |  | -7.36 | (S.C. et al., 2022) |
|  | (Z)-18-Octadec-9-enolide |  | -7.80 | (S.C. et al., 2022) |
|  | 4-Androstene-3,17-dione17-  mono(O-methyloxime |  | -6.91 | (S.C. et al., 2022) |
|  | 5,19-cyclo-5ß-androst-6-ene-3,17-  dione |  | -7.04 | (S.C. et al., 2022) |
|  | 3ꞌ,8,8ꞌ-Trimethoxy-3-piperidyl-  2,2ꞌ-binaphthalene-1,1ꞌ,4,4ꞌ-tetrone |  | -8.06 | (S.C. et al., 2022) |
|  | 6,19-Cycloandrostane-3,7-diol,3ß-  methoxyl |  | -6.66 | (S.C. et al., 2022) |
|  | Oxirane(chloromethyl) |  | -3.69 | (S.C. et al., 2022) |
|  | 9,10-Anthracenedione |  | -5.92 | (S.C. et al., 2022) |
|  | 9,10-Phenanthrenedione |  | -5.54 | (S.C. et al., 2022) |
|  | Amodiaquine |  | -7.46 | (S.C. et al., 2022) |
|  | Amopyroquine |  | -7.60 | (S.C. et al., 2022) |
|  | ß-Tocopherol,o-methyl |  | -9.08 | (S.C. et al., 2022) |
|  | 7H-Pyranol〔2,3-c〕acridin-7-one,  3,12-dihydro-6,11-dihydroxy-3,3,12-  Trimethyl-5-(3-methyl-2-butenyl) |  | -7.92 | (S.C. et al., 2022) |
|  | 1-Azaspiro〔4.5〕dec-3-ene,2-(diphenylmethylene)-4-methyl-1-  phenyl |  | -8.31 | (S.C. et al., 2022) |
|  | Thiophene,2,3-dihydro |  | -4.13 | (S.C. et al., 2022) |
|  | Thiophene,2,5-dihydro |  | -4.11 | (S.C. et al., 2022) |
|  | Bis (2-ethylhexyl) phytalate |  | -8.96 | (S.C. et al., 2022) |
|  | Diisooctyl phythalate |  | -8.89 | (S.C. et al., 2022) |
|  | Phytylhexadecanoate |  | -10.58 | (S.C. et al., 2022) |
|  | (2,5-dioxo-4-imidazolidinyl)carbamic acid |  | -4.96 | (Hui et al., 2020) |
|  | *N*-benzoyl-L-phenylalaninol |  | -7.07 | (Hui et al., 2020) |
|  | 1H-pyrido[3,4-*b*]indol-1-one, 2,3,4,9-tetrahydro-7-hydroxy |  | -5.62 | (Hui et al., 2020) |
|  | Deoxythymidine |  | -6.27 | (Hui et al., 2020) |
|  | (S)-methyl-2-amino-2-phenylacetate |  | -5.84 | (Hui et al., 2020) |
|  | *N*-(2-hydroxy)benzamide |  | -6.14 | (Hui et al., 2020) |
|  | cyclo (PheTyr) |  | -6.56 | (Hui et al., 2020) |
|  | Anisodine |  | Nil | (Hui et al., 2020) |
|  | Hyoscine |  | Nil | (Hui et al., 2020) |
|  | 9-hydroxycanthin-6-one |  | -6.09 | (Hui et al., 2020) |
|  | Anisodamine |  | -7.31 | (Hui et al., 2020) |
|  | 1-ribityl-2,3-diketo-1,2,3,4-tetrahydro-6,7-dimethylquinoxaline |  | Nil | (Hui et al., 2020) |
|  | *N-trans*-ferulyltryptamine |  | -7.42 | (Hui et al., 2020) |
|  | Littorine |  | Nil | (Hui et al., 2020) |
|  | *trans-*feruloyltyramine-4-O-β-D-glucopyranoside |  | -8.49 | (Hui et al., 2020) |
|  | *N-trans-p-*coumaroyloctopamine |  | -6.50 | (Hui et al., 2020) |
|  | *N-cis-p-*coumaroyloctopamine |  | -6.79 | (Hui et al., 2020) |
|  | *N-trans-*feruloyloctopamine |  | -7.13 | (Hui et al., 2020) |
|  | *N-cis-*feruloyloctopamine |  | -7.16 | (Hui et al., 2020) |
|  | *N-trans*-feruloyl-3',4'-dihydroxyphenylethylamine |  | -7.03 | (Hui et al., 2020) |
|  | *N-cis*-coumaroyltyramine |  | -6.83 | (Hui et al., 2020) |
|  | *N-trans*-coumaroyltyramine |  | -6.31 | (Hui et al., 2020) |
|  | canabisine H |  | -9.65 | (Hui et al., 2020) |
|  | Apoatropine |  | Nil | (Hui et al., 2020) |
|  | *N-cis*-feruloyltyramine |  | -7.15 | (Hui et al., 2020) |
|  | *N-trans*-feruloyltyramine |  | -7.33 | (Hui et al., 2020) |
|  | hibiscuwanin B |  | -8.67 | (Hui et al., 2020) |
|  | meteloside F |  | -10.20 | (Hui et al., 2020) |
|  | 3α,6β-ditigloyloxytropan-7β-ol |  | Nil | (Hui et al., 2020) |
|  | 3-(4-hydroxy-3-methoxyphenyl)-*N*-[2-(4-hydroxyphenyl)-2-methoxy- ethyl]acrylamide |  | -7.29 | (Hui et al., 2020) |
|  | *N-cis*-Feruloyl-3'-methoxytyramine |  | -7.45 | (Hui et al., 2020) |
|  | cannabisin E |  | -10.01 | (Hui et al., 2020) |
|  | meteloside B |  | -14.11 | (Hui et al., 2020) |
|  | lyciumamide K |  | -11.198 | (Hui et al., 2020) |
|  | meteloside A |  | -12.23 | (Hui et al., 2020) |
|  | grossamide K |  | -9.00 | (Hui et al., 2020) |
|  | *N-trans*-cinnamoyltyramine |  | -6.47 | (Hui et al., 2020) |
|  | lyciumamide C |  | -10.03 | (Hui et al., 2020) |
|  | cannabisin F |  | -10.69 | (Hui et al., 2020) |
|  | lyciumamide B |  | **-9.51** | (Hui et al., 2020) |
|  | (2a*S*,3a*S*) lyciumamide D |  | -10.59 | (Hui et al., 2020) |
|  | Grossamide |  | -10.49 | (Hui et al., 2020) |
|  | tribulusamide A |  | -10.46 | (Hui et al., 2020) |
|  | cannabisin G |  | -10.64 | (Hui et al., 2020) |
|  | cannabisin D |  | -10.87 | (Hui et al., 2020) |
|  | meteloside G |  | -12.71 | (Hui et al., 2020) |
|  | Benzhydryl [2R-[1(*Z*),2α,3α]]-α-(1-methoxyethylidene)-4-oxo-3-[(phenoxyacetyl)amino]-2-[[(p-tolyl)sulphonyl]thio]azetidine-1-acetate |  | -10.77 | (Hui et al., 2020) |
|  | thoreliamide C |  | -11.86 | (Hui et al., 2020) |
|  | melongenaminde B |  | -10.91 | (Hui et al., 2020) |
|  | daturametelindole B |  | -6.23 | (Hui et al., 2020) |
|  | baimantuoluoamide B |  | Nil | (Hui et al., 2020) |
|  | baimantuoluoamide A |  | -7.11 | (Hui et al., 2020) |
|  | cannabisin L |  | -10.83 | (Hui et al., 2020) |
|  | *N*-[2-(3,4-dihydroxyphenyl)-2-hydroxyethyl]-3-(4-methoxyphenyl)- prop-2-enamide |  | -7.28 | (Hui et al., 2020) |
|  | daturametelindole C |  | -7.13 | (Hui et al., 2020) |
|  | Aurantiamide |  | -7.89 | (Hui et al., 2020) |
|  | Chenoalbicin |  | -10.45 | (Hui et al., 2020) |

**References:**

Bawazeer, S., Rauf, A., & Bawazeer, S. (2020). Potent in Vitro -Glucosidase and β -Secretase Inhibition of Amyrin-Type Triterpenoid Isolated from Datura metel Linnaeus (Angel’s Trumpet) Fruits. In *BioMed Research International* (Vol. 2020). https://doi.org/10.1155/2020/8530165

Cholilalah, Rois Arifin, A. I. H. (1967). Determination Of Antiproliferative Components Of Datura Metel L. Extracts By Gc-Ms And Hplc-Tof/Ms Analysis. *Angewandte Chemie International Edition, 6(11), 951–952.*, 82–95.

Hui, Y. S., Yan, L., Qi, W., Ping, S. Y., Wei, G., Yuan, L., You, Y. B., & Xue, K. H. (2020). Identification and quantification of alkaloid compounds from different parts and production areas of Datura metel L. *Heterocycles*, *100*(4), 568–584. https://doi.org/10.3987/COM-20-14228

Jabeen, N., Javaid, A., & Ahmed, E. (2022). Antifungal Activity and Phytochemical Profile of Chloroformsoluble Fraction of Datura Metel Fruit. *Journal of Animal and Plant Sciences*, *32*(4), 1085–1091. https://doi.org/10.36899/JAPS.2022.4.0512

Lawal, B. A., Ayipo, Y. O., Adekunle, A. O., Amali, M. O., Badeggi, U. M., Alananzeh, W. A., & Mordi, M. N. (2023). Phytoconstituents of Datura metel extract improved motor coordination in haloperidol-induced cataleptic mice: Dual-target molecular docking and behavioural studies. *Journal of Ethnopharmacology*, *300*(September 2022), 115753. https://doi.org/10.1016/j.jep.2022.115753

Liu, Y., Guan, W., Lu, Z. kun, Guo, R., Xia, Y. G., Lv, S. W., Yang, B. Y., & Kuang, H. X. (2019). New sesquiterpenoids from the stems of Datura metel L. *Fitoterapia*, *134*(February), 417–421. https://doi.org/10.1016/j.fitote.2019.02.029

Liu, Y., Guan, W., Yang, C. L., Luo, Y. M., Liu, Y., Zhou, Y. Y., Liu, L. N., Yang, B. Y., & Kuang, H. X. (2020). Steroids with potential anti-inflammatory activity from the roots of datura metel l. *Canadian Journal of Chemistry*, *98*(2), 74–78. https://doi.org/10.1139/cjc-2019-0282

Mai, N. T., Cuc, N. T., Anh, H. L. T., Nhiem, N. X., Tai, B. H., Yen, P. H., Quang, T. H., Minh, C. Van, Kim, K. W., Kim, Y. C., Oh, H., & Kiem, P. Van. (2017). Two new guaiane sesquiterpenes from Datura metel L. with anti-inflammatory activity. *Phytochemistry Letters*, *19*, 231–236. https://doi.org/10.1016/j.phytol.2017.01.011

Okwu, D. E., & Igara, E. C. (2009). Isolation, characterization and antibacterial activity of alkaloid from Datura metel Linn leaves. *African Journal of Pharmacy and Pharmacology*, *3*(5), 277–281.

Prasathkumar, M., Anisha, S., Khusro, A., Mohamed Essa, M., Babu Chidambaram, S., Walid Qoronfleh, M., Sadhasivam, S., Umar Khayam Sahibzada, M., Alghamdi, S., Almehmadi, M., Abdulaziz, O., Khandaker, M. U., Faruque, M. R. I., & Emran, T. Bin. (2022). Anti-pathogenic, anti-diabetic, anti-inflammatory, antioxidant, and wound healing efficacy of Datura metel L. leaves. *Arabian Journal of Chemistry*, *15*(9), 104112. https://doi.org/10.1016/j.arabjc.2022.104112

S.C., C., C.O., S., H.O., C., L.O., O., B.O., C., & N.U., F. (2022). Phytochemical Evaluation and Functional Group Detection of Ethanolic Leaf and Root Extracts of datura metel. *African Journal of Biology and Medical Research*, *5*(1), 30–52. https://doi.org/10.52589/ajbmr-96zvvkmm

SHUAIB, M. A. & M. (1996). *characterization-of-the-chemical-constituents-of-datura-metel-linn.pdf*.

Takeshi, N. (1977). NII-Electronic Library Service. *Chemical Pharmaceutical Bulletin*, *57*(534), 364–370.

Tan, J., Liu, Y., Cheng, Y., Sun, Y., Pan, J., Guan, W., Li, X., Huang, J., Jiang, P., Guo, S., Kuang, H., & Yang, B. (2020). New withanolides with anti-inflammatory activity from the leaves of Datura metel L. *Bioorganic Chemistry*, *95*(December 2019), 103541. https://doi.org/10.1016/j.bioorg.2019.103541

Yang, B. you, Zhou, Y. qiang, Liu, Y., Lu, Z. kun, & Kuang, H. xue. (2018). Ent-kaurane diterpenoids from the pericarps of Datura metel L. acted on the vascular endothelial cells via TRPC6 and NF-κB protein. *Medicinal Chemistry Research*, *27*(1), 115–121. https://doi.org/10.1007/s00044-017-2046-z
